# Painlevé Kernels and Surface Defects at Strong Coupling

**DOI:** 10.1007/s00023-024-01469-4

**Published:** 2024-07-14

**Authors:** Matijn François, Alba Grassi

**Affiliations:** 1 Section de Mathématiques, Université de Genève, 1211, Genève 4, Switzerland; 2 Theoretical Physics Department, CERN, 1211, Geneva 23, Switzerland

## Abstract

It is well established that the spectral analysis of canonically quantized four-dimensional Seiberg–Witten curves can be systematically studied via the Nekrasov–Shatashvili functions. In this paper, we explore another aspect of the relation between ${\mathcal {N}}=2$ supersymmetric gauge theories in four dimensions and operator theory. Specifically, we study an example of an integral operator associated with Painlevé equations and whose spectral traces are related to correlation functions of the 2d Ising model. This operator does not correspond to a canonically quantized Seiberg–Witten curve, but its kernel can nevertheless be interpreted as the density matrix of an ideal Fermi gas. Adopting the approach of Tracy and Widom, we provide an explicit expression for its eigenfunctions via an ${{\,\mathrm{O(2)}\,}}$ matrix model. We then show that these eigenfunctions are computed by surface defects in ${{\,\mathrm{SU(2)}\,}}$ super Yang–Mills in the self-dual phase of the $\Omega$-background. Our result also yields a strong coupling expression for such defects which resums the instanton expansion. Even though we focus on one concrete example, we expect these results to hold for a larger class of operators arising in the context of isomonodromic deformation equations.

## Introduction

Building upon the work of Seiberg and Witten [1, 2], important results have been obtained for ${\mathcal {N}}=2$ supersymmetric gauge theories in four dimensions. One remarkable achievement is the exact evaluation of the path integral, made possible thanks to localization techniques and the introduction of the $\Omega$-background [3–6]. This led to the discovery of a new class of special functions, the so-called Nekrasov functions [5, 6], which today have found a wide range of applications in various fields of mathematics and theoretical physics. Examples of such functions are given in (3.10) and (6.1). Despite the exceptional control Nekrasov functions grant us over the weak gauge coupling regime, a strong coupling expansion requires alternative methods. This is one of the motivations behind the present work. In addition, this simultaneously explores a particular extension of the correspondence relating ${\mathcal {N}}=2$ supersymmetric gauge theories in four dimensions to the spectral theory of quantum mechanical operators on the space of square integrable functions $L^2({{\mathbb {R}}})$.

The first concrete example of such correspondence was obtained in [7], where the authors showed how to use ${\mathcal {N}}=2$ supersymmetric gauge theories in the four-dimensional Nekrasov–Shatashvili (NS) phase of the $\Omega$-background $(\epsilon _2=0)$ to solve the spectral theory of a certain class of ordinary differential equations. For example, the quantization condition for the operator spectrum corresponds to the quantization of the twisted superpotential [7–11], while the eigenfunctions are computed from the surface defect partition functions [12–25]. More recently, explicit expressions for the Fredholm determinants [26, 27] and the connection coefficients [22] of such differential equations have been derived in closed form.^1^ See also [31–39] for related development in the context of WKB and four-dimensional gauge theories, and [40] for a different quantization scheme in higher-rank gauge theories. All the operators appearing in the above setup can be obtained via the canonical quantization of four-dimensional Seiberg–Witten (SW) curves [7, 8, 41], or equivalently, by considering the semiclassical limit of BPZ equations [42].

In this paper, we explore another facet of the interplay between spectral theory and supersymmetric gauge theories. On the gauge theory side, we focus on four-dimensional ${\mathcal {N}}=2$ gauge theories in the *self-dual* phase of the $\Omega$-background ($\epsilon _1=-\epsilon _2=\epsilon$), while on the operator theory side we study a class of operators which do *not* correspond to canonically quantized four-dimensional SW curves. These operators originally appeared in the framework of isomonodromic deformation equations [43–47]. Their relevance in the context of four-dimensional supersymmetric gauge theories and topological string theory was pointed out in [48–51], in close connection with the TS/ST duality [52–55] and the isomonodromy/CFT/gauge theory correspondence

[56–61]. Geometrically, we construct such operators from mirror curves to toric CY manifolds, after implementing a suitable canonical transformation combined with a special scaling limit [48, 49], see Sects. 3.23.4.

Here we focus on a specific example of such an operator which is associated with the Painlevé $\mathrm III_3$ equation, and whose spectral traces compute correlation functions in the 2d Ising model [43, 46, 62]. Its integral kernel $\rho (x,y)$ on ${{\mathbb {R}}}$ reads^2^

1.1 $$\begin{aligned} \rho (x,y)={\textrm{e}^{-4 t^{1/4}\cosh x}\textrm{e}^{-4 t^{1/4}\cosh y}\over \cosh \left( x-y\over 2\right) } \, , \end{aligned}$$

and the corresponding Fredholm determinant $\det \left( 1+\kappa \rho \right)$ computes the tau function of Painlevé $\mathrm III_3$ [43]. For $t>0$, the kernel (1.1) is positive and of trace class on $L^2({{\mathbb {R}}})$; hence, the corresponding operator has a discrete, positive spectrum $\left\{ E_n\right\} _{n\geqslant 0}$, with real-valued, square integrable eigenfunctions $\left\{ \varphi _n(x,t)\right\} _{n\geqslant 0}$,

1.2 $$\begin{aligned} \int \limits _{\mathbb {R}} \textrm{d}y \ \rho \left( x, y\right) \varphi _n \left( y, t \right) = E_n \varphi _n\left( x, t \right) \, . \end{aligned}$$

As we review in Sect. 3.2, the spectrum is computed by the Nekrasov function of 4d, ${\mathcal {N}}=2$, ${{\,\mathrm{SU(2)}\,}}$ super Yang–Mills (SYM) in the self-dual phase of the $\Omega$-background [48], see (3.7) and (3.8). The purpose of this paper is to study the eigenfunctions of (1.1) and relate them to surface defects in the same gauge theory. Specifically, we find that the eigenfunctions $\varphi _n\left( x, t \right)$ are the Zak transform of the sum of two partition functions of surface defects, which are simply related by a change in some parameters. See Eq. (2.6) for the explicit expression. We test this proposal numerically in Sect. 6.4. The relevance of this result is twofold. On the one hand, it provides an efficient description of eigenfunctions of (1.1) at small *t*. On the other hand, it offers a tool to resum both the instanton expansion and the $\epsilon$ expansion in the defect partition function, hence allowing to probe the gauge theory at strong coupling (large *t*). This is possible because, following [47, 63], we can provide an alternative matrix model description for such eigenfunctions (2.1), which is exact in *t* and hence exact in the gauge coupling.

## Summary

### Results

The paper can be summarized as follows. Adopting the approach of [47], which is nicely summarized in [63, sec. 2.3], we construct eigenfunctions of (1.1) from expectation values of a determinant-like expression,

2.1 $$\begin{aligned}{} & {} \Xi _{\pm }\left( x,t,\kappa \right) = \textrm{e}^{-4 t^{1/4}\cosh x} \textrm{e}^{\pm x/2} \sum _{N\geqslant 0}(\pm \kappa )^N \Psi _N \left( \textrm{e}^x , t \right) \, , \qquad \qquad x \in {{\mathbb {R}}}\, , \end{aligned}$$

2.2 $$\begin{aligned}{} & {} \begin{aligned} \Psi _N(z,t) = {1\over N! }\int \limits _{{{\mathbb {R}}}^N_{>0}}\left( \prod _{i=1}^N{\textrm{d}z_i \over z_i} {z-z_i\over z+z_i} ~ \exp \left( -{4 t^{1/4}} \left( z_i + z_i^{-1}\right) \right) \prod _{j = i+1}^N \left( { z_i-z_j \over z_i + z_j }\right) ^2 \right) \, . \end{aligned} \nonumber \\ \end{aligned}$$

More precisely, (2.1) are square integrable eigenfunctions $\varphi _n$ of (1.1) if we set $\kappa =-E_n^{-1}$, where $E_n$ is an eigenvalue of the operator (1.1),

2.3 $$\begin{aligned} \varphi _n(x,t)=\Xi _+\left( x,t,-{1\over E_n}\right) =(-1)^n \Xi _-\left( x,t,-{1\over E_n}\right) \, . \end{aligned}$$

In Sect. 6, we show that (2.1) and (2.2) are explicitly related to surface defects in 4d, ${\mathcal {N}}=2$, ${{\,\mathrm{SU(2)}\,}}$ SYM in the self-dual phase ($\epsilon _1=-\epsilon _2=\epsilon$) of the $\Omega$-background^3^. We consider the surface defect which is engineered using the open topological vertex with a D-brane on the external leg, see Appendix A for details. Using the explicit vertex expression of Appendix A, one can see that this corresponds to the special case of a 2d/4d defect called a type II defect in [17, sec. 2.3.3]^4^. Hence, we denote its partition function by $Z^{\textrm{II}}_{\textrm{tot}} (q, t, \sigma )$. The explicit expression is given in (6.1) and (6.4). In the gauge theory, we typically use

2.4 $$\begin{aligned} q = {y \over 2 \epsilon } \, , \qquad \qquad t = \left( {\Lambda \over \epsilon }\right) ^4 \, , \qquad \qquad \sigma = \textrm{i} {a\over 2 \epsilon } \, , \end{aligned}$$

where *y* is the position of the defect^5^, $\epsilon =\epsilon _1=-\epsilon _2$ is the $\Omega$-background parameter, *a* is the Coulomb branch parameter, and $\Lambda \sim \textrm{e}^{-1/g_{\textrm{YM}}^2}$ is the instanton counting parameter, with $g_{\textrm{YM}}$ the gauge coupling. The relation between the determinant-like expression (2.1) and the defect partition function $Z^{\textrm{II}}_{\textrm{tot}}$ is given in (6.7) and reads

2.5 $$\begin{aligned}{} & {} \Xi _\pm \left( x , t , \frac{\cos (2 \pi \sigma )}{ 2 \pi } \right) = { \textrm{e}^{3\zeta '(-1)}\textrm{e}^{4 \sqrt{t}}\over 2^{11/12} \pi ^{3/2} t^{3/16} }\int \limits _{{{\mathbb {R}}}} \textrm{d}q ~ \textrm{e}^{\textrm{i}{2} q x}\nonumber \\{} & {} \quad \sum _{k \in {{\mathbb {Z}}}} \left( Z^{\textrm{II}}_{\textrm{tot}}\left( \pm q, t, \sigma + k \right) + Z^{\textrm{II}}_{\textrm{tot}}\left( \mp q-{\textrm{i}\over 2}, t, \sigma + k +{1\over 2}\right) \right) \, . \end{aligned}$$

**Fig. 1:**
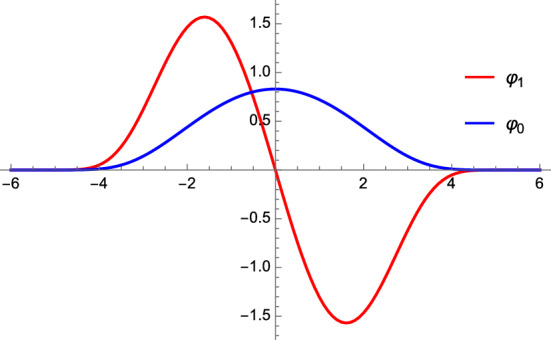
The first (blue) and second (red) eigenfunction of (1.1) computed using the surface defect expression in (2.6) for $t = 1 / 100 \pi ^8$

The quantization condition for the energy spectrum of (1.1) was derived in [48], see (3.7) and (3.8). By evaluating the defect partition function on the right-hand side of (2.5) at the corresponding quantized values of $\sigma = 1 / 2 + \textrm{i}\sigma _n$, we obtain the eigenfunctions $\varphi _n$ of (1.1),

2.6 $$\begin{aligned}{} & {} \varphi _n(x, t) = { \textrm{e}^{3\zeta '(-1)}\textrm{e}^{4 \sqrt{t}}\over 2^{11/12} \pi ^{3/2} t^{3/16} }\int \limits _{{{\mathbb {R}}}} \textrm{d}q ~ \textrm{e}^{\textrm{i}{2} q x}\nonumber \\{} & {} \quad \sum _{k \in {{\mathbb {Z}}}} \left( Z^{\textrm{II}}_{\textrm{tot}}\left( q , t, k + \frac{1}{2} + \textrm{i}\sigma _n \right) + Z^{\textrm{II}}_{\textrm{tot}}\left( - q - {\textrm{i}\over 2}, t, k + \textrm{i}\sigma _n \right) \right) , \end{aligned}$$

where $\sigma _n \in {\mathbb {R}}_{>0}$ are solutions to (3.8). The eigenfunctions $\varphi _0$ and $\varphi _1$ are shown in Fig. 1.

In Sect. 6 and Appendix B, we show that we can equivalently write (2.5) as

2.7 $$\begin{aligned}{} & {} \int \limits _{ {{\mathbb {R}}}+\textrm{i}\sigma _*}{\textrm{d}\sigma } {\tan \left( 2 \pi \sigma \right) \over \left( 2 \cos (2 \pi \sigma )\right) ^N} \left( Z^{\textrm{II}}_{\textrm{tot}} \left( q, t, \sigma \right) + Z^\textrm{II}_{\textrm{tot}}\left( - q - {\textrm{i}\over 2}, t, \sigma +{1\over 2} \right) \right) \nonumber \\{} & {} \quad = \textrm{i} {2^{11/12}\sqrt{\pi }{ t^{3/16} } \over \textrm{e}^{3\zeta '(-1)}\textrm{e}^{4 \sqrt{t}} \left( 4 \pi \right) ^N} \int \limits _{\mathbb {R}} {\textrm{d}x} ~ \textrm{e}^{-\textrm{i}{2} q x} \textrm{e}^{ - 4 t^{1/4} \cosh x} \textrm{e}^{x/2} \Psi _N \left( \textrm{e}^{{x}}, t \right) \, , \end{aligned}$$

where $\sigma _*$ is such that $0< \sigma _* < |\textrm{Re}(q)|$ if $\textrm{Re}(q) \ne 0$, and simply $\sigma _* > 0$ if $\textrm{Re}(q)=0$. This choice of $\sigma ^*$ guarantees that the integration over $\sigma$ in (2.7) avoids the poles of the integrand. Let us elaborate more on the meaning of (2.7).

- The Fourier transform on the right-hand side of (2.7) relates two types of defects [17, Sect. 2.3.3], or more precisely, two phases of the same defect [19, Sect. 4.2]^6^. In particular, while $Z^{\textrm{II}}_{\textrm{tot}}(q, t, \sigma )$ is geometrically engineered in topological string theory by inserting a brane on the external leg of the toric diagram, its Fourier transform with respect to the defect variable *q* makes contact with a brane in the inner edge of the toric diagram [19], see also [41, 73–75]^7^. Following [17, Sect. 2.3.3], we refer to the inverse Fourier transform of a type II defect as a type I defect^8^. Via the AGT correspondence [76], the latter is realized in Liouville CFT by considering the five-point function of four primaries with one degenerate field, the so-called $\Phi _{2,1}$ field [24, 25, 68, 77–79]. One can equivalently realize this defect by coupling the four-dimensional theory to a two-dimensional theory, see, for instance, [15–20, 71, 80–82] and references therein. This also means that we could get rid of the inverse Fourier transform on the right-hand side of (2.5) by replacing the partition function of the type II defect $Z^{\textrm{II}}$ with the partition function of the type I defect $Z^{\textrm{I}}$. The instanton counting-like expression of type I defect can be found, for instance, in [15]; however, we will not use such expression here as we will mainly focus on type II defects. The reason for this is that, in the latter case, the gauge theoretic expression is represented by a convergent series in *t*, whose coefficients are exact rational functions of $\sigma$ and *q*. Conversely, for the type I defect, the gauge theoretic expression is more cumbersome, involving double series expansions in both *t* and in *q*.
- The integral over $\sigma$ on the left-hand side of (2.7) is responsible for the change of frame: it brings us from the weakly coupled electric frame, where $Z^\textrm{II}_{\textrm{tot}}$ is defined, to the strongly coupled magnetic frame, which is the suitable frame to describe the magnetic monopole point of SYM, see Sect. 5.

In summary, (2.7) means that the matrix model average (2.2) computes the type I surface defect partition function of 4d, ${\mathcal {N}}=2$, ${{\,\mathrm{SU(2)}\,}}$ SYM, in the *self-dual phase* of the $\Omega$-background ($\epsilon _1=-\epsilon _2=\epsilon$), and in the *magnetic frame*. In this identification, $z = \exp \left( x\right)$ is related to the position of the defect and the ’t Hooft parameter of the matrix model is identified with the dual period, $N \epsilon = a_D$.

Note also that (2.2) is exact both in $\Lambda$ and in $\epsilon$; it resums the instanton expansion of the defect partition function and provides an explicit interpolation from the weak to the strong coupling region. The $1/\Lambda$ expansion can be obtained straightforwardly from (2.2) since it corresponds to expanding the matrix model around its Gaussian point, see [51, Sect. 5] and references therein.

### Derivation

Let us briefly comment on the derivation of Eqs. (2.5), (2.6), and (2.7). Firstly, we obtained these results by analyzing the large *N* expansion of the matrix models (2.2) and then extrapolating to finite *N*. Secondly, part of the idea also comes from the open version of the TS/ST correspondence [63, 83], see Sects. 3.4 and 7. By combining these two approaches we obtained (2.5–2.7), which we further tested numerically. However, we do not have a rigorous mathematical proof of these results.

This paper is structured as follows. In Sect. 3, we give an overview of the well-established relationship between the modified Mathieu operator and the four-dimensional, ${\mathcal {N}}=2$, ${{\,\mathrm{SU(2)}\,}}$ SYM in the NS phase of the $\Omega$-background. We then present the connection between the operator (1.1) and the same gauge theory, but in the self-dual phase of the $\Omega$-background. In Sect. 4, we compute the planar resolvent of (2.2) as well as the planar two-point function and show how the Seiberg–Witten geometry emerges from it. In Sect. 5, we show that the ’t Hooft expansion of (2.2) reproduces the $\epsilon$ expansion of the type I self-dual surface defect in the magnetic frame. To establish this connection, we rely on two crucial findings. Firstly, according to the results presented in [68], the $\epsilon$ expansion of the self-dual type I surface defect in the electric frame is determined by topological recursion [84]. Secondly, the self-dual surface defect (or, more generally, the open topological string partition function) behaves as a wave function under a change of frame [85]. In Sect. 6, we test (2.7) numerically for finite *N* and analytically in a 1/*N* expansion, and we verify (2.6) numerically.

## Preparation: Spectral Theory and 4d, ${\mathcal {N}}=2$ Gauge Theory

### Known: Differential Operators and the NS Phase of the $\Omega$-Background

Let us start by reviewing the correspondence relating ordinary differential equations to four-dimensional ${\mathcal {N}} = 2$ gauge theories in the *NS phase* of the $\Omega$-background, where $\epsilon _2 = 0$ and $\epsilon _1 = \epsilon \ne 0$ [7]. In this paper, we focus on ${{\,\mathrm{SU(2)}\,}}$ SYM.

Consider the so-called modified Mathieu operator $\textrm{O}_{\textrm{Ma}}$ acting as

3.1 $$\begin{aligned} \textrm{O}_{\textrm{Ma}}~ \varphi (x,t)=\left( -\partial _x^2+\sqrt{t} \cosh \left( x \right) \right) \varphi (x,t)~. \end{aligned}$$

If $t > 0$, the operator (3.1) has a positive discrete spectrum with square integrable eigenfunctions. One can make the connection to gauge theory by noting that the modified Mathieu operator corresponds to the canonically quantized SW curve of ${{\,\mathrm{SU(2)}\,}}$ SYM, if we set $t = \left( \Lambda / \epsilon \right) ^4$ as in (2.4). This relationship was exploited in [7], showing how gauge theory can be efficiently used to find the spectrum of (3.1). The first ingredient in this relation is the quantization condition of the twisted superpotential which reads

3.2 $$\begin{aligned} \partial _\sigma F^{\textrm{NS}}(t, \sigma ) = \textrm{i} 2 \pi n \, , \qquad \qquad \qquad n \in {\mathbb {N}} \, , \end{aligned}$$

where $F^{\textrm{NS}}$ is the NS free energy and $\sigma = \textrm{i} a / 2 \epsilon$ as in (2.4). The small *t* or weak coupling expansion is given by

3.3 $$\begin{aligned} F^{\textrm{NS}}(t, \sigma ){} & {} = - \psi ^{(-2)}(1-2 \sigma ) - \psi ^{(-2)}(1+2 \sigma ) + \sigma ^2 \log (t) \nonumber \\{} & {} \quad + \frac{2 t}{ 4\sigma ^2 - 1} + \frac{\left( 20 \sigma ^2 + 7 \right) t^2}{\left( 4 \sigma ^2 - 1 \right) ^3 \left( 4 \sigma ^2 - 4 \right) } + {\mathcal {O}}\left( t^3\right) \, , \end{aligned}$$

where $\psi$ is the polygamma function of order $-2$. Higher-order terms in *t* in (3.3) can be computed by using combinatorics of Young diagrams [86–88], see [89] for a review and further list of references. The resulting expansion in (3.3) is convergent^9^ when $2\sigma \notin {{\mathbb {Z}}}$. Equation (3.2) has then a discrete set of solutions $\left\{ \sigma _n\right\} _{n\geqslant 0}$ for a fixed value of $t > 0$. The quantum Matone relation [92, 93],

3.4 $$\begin{aligned} E = -t \partial _t F^{\textrm{NS}}\left( t, \sigma \right) \, . \end{aligned}$$

gives at last the connection to the spectrum $\left\{ E_n\right\} _{n\geqslant 0}$ of the modified Mathieu operator (3.1).

There is a parallel development for the eigenfunctions, but one has to consider the four-dimensional partition function with the insertion of a type I defect^10^ in the NS phase of the $\Omega$-background, see [12–19] and references there.

### New: Painlevé Kernels and the Self-Dual Phase of the $\Omega$-Background

In this work, we consider another class of operators whose spectral properties are encoded in the gauge theory partition functions in the *self-dual phase* of the $\Omega$-background, where $\epsilon _1+\epsilon _2=0$. We focus on the four-dimensional, ${\mathcal {N}}=2$, ${{\,\mathrm{SU(2)}\,}}$ SYM. In this case, the relevant operator $\rho$ has the integral kernel $\rho (x,y)$ given by [48]

3.5 $$\begin{aligned} \mathrm{\rho }(x,y) = {\sqrt{v(x)}\sqrt{v(y)}\over \cosh \left( {x-y\over 2}\right) } \, , \qquad v(x)=\textrm{e}^{-{8 t^{1/4}} \cosh (\mathrm x)} \, . \end{aligned}$$

This operator $\rho$ can be seen as the density matrix of an ideal Fermi gas in an external potential $- \log \left[ v\left( x\right) \right]$ [62]. We therefore refer to (3.5) as a Fermi gas operator. Unlike the example of the modified Mathieu operator, the relation of this operator to the quantization of the four-dimensional ${{\,\mathrm{SU(2)}\,}}$ Seiberg–Witten curves is far from obvious. This connection was originally found in [48] as a prediction of the TS/ST correspondence [52]. It can be understood as a consequence of the fact that the operator (3.5) can be constructed starting from the quantum mirror curve to local ${{\mathbb {F}}}_0$, and implementing a particular scaling limit, as we review in Sect. 3.4. However, we do not yet have a direct method to derive (3.5) using any quantization scheme that starts from the four-dimensional SW curve. We have to rely on the TS/ST correspondence. See also the discussion at the end of Sect. 3.4.

For $t>0$ (3.5) is a trace class operator on $L^2({{\mathbb {R}}})$ with a positive discrete spectrum $\left\{ E_n\right\} _{n \geqslant 0}$,

3.6 $$\begin{aligned} \int \limits _{\mathbb {R}} \textrm{d}y \ \rho \left( x, y\right) \varphi _n \left( y, t \right) = E_n \varphi _n\left( x, t \right) \, . \end{aligned}$$

It was shown in [48] that the spectrum is given by

3.7 $$\begin{aligned} E_n^{-1}=-{1\over 2\pi }\cos \left( 2 \pi \left( {1\over 2}+\textrm{i}{\sigma _n}\right) \right) \, , \end{aligned}$$

where $\sigma _n \in {{\mathbb {R}}}_{>0}$ are solutions to

3.8 $$\begin{aligned} \sum _{k \in {{\mathbb {Z}}}} Z^{\textrm{Nek}}\left( t, {1\over 2} + \textrm{i}\sigma _n + k \right) =0 \, . \end{aligned}$$

This result follows from the identity [48, eqs 3.26, 3.49, 3.55] [49, eq. 2.19]

3.9 $$\begin{aligned} \det \left( 1+ {\cos (2\pi \sigma )\over 2\pi }\rho \right) =2^{1/12}\textrm{e}^{3\zeta '(-1)}t^{-1/16}\textrm{e}^{4\sqrt{t}}\sum _{k \in {{\mathbb {Z}}}} Z^{\textrm{Nek}}(t, \sigma + k ) \, , \end{aligned}$$

which was proved using the theory of Painlevé equations. The function $Z^{\textrm{Nek}}(t, \sigma )$ in (3.8) and (3.9) is the Nekrasov partition function in the self-dual phase of the $\Omega$-background,

3.10 $$\begin{aligned} Z^{\textrm{Nek}}(t, \sigma ) ={t^{\sigma ^2} \over G(1-2\sigma )G(1+2\sigma )} \left( 1+\frac{t}{2 \sigma ^2}+ \frac{\left( 8 \sigma ^2+1\right) t^2}{4 \sigma ^2 \left( 4 \sigma ^2-1\right) ^2}+{\mathcal {O}}(t^3)\right) \, , \nonumber \\ \end{aligned}$$

with *G* the Barnes G-function. Higher-order terms in *t* are defined systematically by using combinatorics of Young diagrams, see [90, eqs. 3.4, 3.6] for the precise definition. The convergence of this series is shown in [90] for any $t>0$ and fixed $2\sigma \notin {{\mathbb {Z}}}$. Even though $Z^{\textrm{Nek}}(t, \sigma )$ has poles when $2\sigma \in {{\mathbb {Z}}}$, the sum on the right-hand side of (3.9) removes these poles, and the resulting expression is well defined for any complex value of $\sigma$ [90, 94]. Let us also note that the Nekrasov function is often expressed using $\Lambda$, *a* and $\epsilon$, which are related to *t* and $\sigma$ via (2.4)

3.11 $$\begin{aligned} t = \left( \frac{\Lambda }{\epsilon }\right) ^4 \, , \qquad \qquad \qquad \sigma ={\textrm{i}}\frac{a}{2 \epsilon } \, . \qquad \end{aligned}$$

It is useful to write the Fredholm determinant on the left-hand side of (3.9) by using the spectral traces,

3.12 $$\begin{aligned}{} & {} \det \left( 1+ \kappa \rho \right) =\sum _{N\geqslant 0} \kappa ^N Z(t, N)~, \end{aligned}$$

3.13 $$\begin{aligned}{} & {} Z( t, N)= {1\over N!}\sum _{s\in S_N}(-1)^{\textrm{sgn }(s)}\int \limits _{{{\mathbb {R}}}} \textrm{d}^N x\prod _{i=1}^N\rho (x_i, x_{s(i)})~, \end{aligned}$$

where $S_N$ is the permutation group of *N* elements. The Cauchy identity allows us to write (3.13) as [62]

3.14 $$\begin{aligned} Z(t, N) = {1\over N! }\int \limits _{{{\mathbb {R}}}^N_{>0}}\left( \prod _{i=1}^N{\textrm{d}z_i \over z_i} \textrm{e}^-{4 t^{1/4}} \left( z_i+z_i^{-1}\right) \prod _{j = i+1}^N \left( { z_i-z_j \over z_i + z_j }\right) ^2 \right) \, , \end{aligned}$$

which can be analyzed in the regime $t \rightarrow + \infty$, since this corresponds to expanding the potential around its Gaussian point. It was found in [48] that the matrix model (3.14) computes the partition function of ${\mathcal {N}}=2$, ${{\,\mathrm{SU(2)}\,}}$ SYM in the self-dual phase of the $\Omega$-background and in the magnetic frame^11^. The relation to the Nekrasov function (3.10) can be obtained from (3.9) and reads [49, eq. 2.28]

3.15 $$\begin{aligned} Z(t, N) = - \textrm{i} (4 \pi )^N 2^{1/12} \textrm{e}^{3\zeta '(-1)} t^{-1/16}\textrm{e}^{4 \sqrt{t}} \int \limits _{ {{\mathbb {R}}}+\textrm{i}\sigma _*}{\textrm{d}\sigma } \frac{\tan \left( 2 \pi \sigma \right) }{\left( 2 \cos (2 \pi \sigma )\right) ^N} Z^{\textrm{Nek}}\left( t, \sigma \right) , \nonumber \\ \end{aligned}$$

where^12^$\sigma _* > 0$. Therefore, the matrix model (3.14) provides a resummation of the instanton expansion in the Nekrasov function (3.10), which is an expansion around $t=0$. In this context, we identify the ’t Hooft parameter of the matrix model, $N \epsilon$, with the dual or magnetic period in SW theory^13^

3.16 $$\begin{aligned} a_D=N \epsilon ~. \end{aligned}$$

The equality (3.15) was demonstrated in [48, 49]. Finally, we emphasize that (3.14) is exact both in the instanton counting parameter $\Lambda$ and in the $\Omega$-background parameter $\epsilon$. When we expand (3.14) at large $\Lambda$ while keeping $\epsilon$ and $a_D$ fixed, we obtain an analogous expansion to that found when performing a large-time expansion in isomonodromic deformation equations [51, 90, 96–100]. On the matrix model side, this is an expansion around the Gaussian point. Similarly, if we expand at small $\epsilon$ while keeping $\Lambda$ and $a_D$ fixed, we recover the expansion resulting from the holomorphic anomaly algorithm [101, 102].

The goal of this work is to extend these results to the eigenfunctions of (3.5), which on the gauge theory side corresponds to inserting surface defects. As a first observation, we note that the kernel (3.5) falls in the class of operators studied in [47], and more recently in [63, Sect. 2]. In particular, following [47, 63] we can construct eigenfunctions of (3.5) using the matrix model (3.14). Let us define

3.17 $$\begin{aligned} \Xi _{\pm }\left( x,t, \kappa \right)= & {} \textrm{e}^{-{4 t^{1/4} } \cosh ( x)} \textrm{e}^{\pm x/2}\sum _{N\geqslant 0}(\pm \kappa )^N \Psi _N \left( \textrm{e}^x , t \right) , \qquad \qquad x\in {{\mathbb {R}}}~, \nonumber \\ \end{aligned}$$

3.18 $$\begin{aligned} \Psi _N(z,t)= & {} {1\over N! }\int \limits _{{{\mathbb {R}}}^N_{>0}}\left( \prod _{i=1}^N{\textrm{d}z_i \over z_i} {z-z_i\over z+z_i} ~ \exp \left( -{4 t^{1/4}} \left( z_i + z_i^{-1}\right) \right) \right. \nonumber \\{} & {} \left. \prod _{j = i+1}^N \left( { z_i-z_j \over z_i + z_j }\right) ^2 \right) . \end{aligned}$$

The $\Xi _{\pm }(x,t,\kappa )$ in (3.17) are then square integrable eigenfunctions $\varphi _n(x, t)$ of (3.5) if we evaluate them at $\kappa =-E_n^{-1}$,

3.19 $$\begin{aligned} \varphi _n(x, t) = \Xi _{+}\left( x,t,-{1\over E_n}\right) = \left( -1\right) ^n\Xi _{-}\left( x,t,-{1\over E_n}\right) . \end{aligned}$$

This can be verified by using [63, eqs. 2.46, 2.59] and $\varphi _n(x, t)=(-1)^n \varphi _n(-x, t)$. We will argue in the forthcoming sections that the matrix model with insertion $\Psi _N(z,t)$ corresponds to a surface defect in four-dimensional, ${\mathcal {N}}=2$, ${{\,\mathrm{SU(2)}\,}}$ SYM in the *self-dual phase* of the $\Omega$-background and in the *magnetic frame*.

### Comment on Blowup Equations

It was first pointed out in [103] that the five-dimensional NS and self-dual partition functions are closely connected, which was subsequently demonstrated using Nakajima–Yoshioka blowup equations in [104]. The interplay between these two phases of the $\Omega$-background was extended to surface defects in four dimensions in [105, 106]. Applications in the context of Painlevé equations are discussed in [100, 105–109]. The relevance of blowup equations in the context of resurgence was also recently investigated in [110].

Given such results, it is natural to wonder whether blowup equations can be used to relate the spectrum and eigenfunctions of (3.1) and (3.5). Regarding the spectrum, the blowup formula presented in [108, eq. 5.7] reveals a one-to-one correspondence between the solutions $\{\sigma _n\}_{n\geqslant 0}$ of (3.2) and the solutions $\{\sigma _n\}_{n\geqslant 0}$ of (3.8). However, to obtain the spectrum we further need the quantum Matone relation (3.4) on the Mathieu side and the relation (3.7) on the Fermi gas side. These two relations are very different, and therefore, the spectrum of (3.1) and (3.5) is related in a highly non-trivial way. It would be interesting to see if blowup equations in the presence of defects [105, 106] could be used to establish a map between the eigenfunctions of these two operators.

### Two Limits of Quantum Mirror Curves

Both operators (3.1) and (3.5) can be obtained as different limits of the quantized mirror curve corresponding to the toric Calabi–Yau (CY) manifold known as local ${\mathbb {F}}_0$, which we review here briefly.

It is well known that four-dimensional ${\mathcal {N}}=2$ supersymmetric theories can be engineered by using topological string theory on toric CY manifolds [111–113]. The partition function of refined topological string theory is then identified with the partition function of a five-dimensional ${\mathcal {N}}=1$ theory on ${{\mathbb {R}}}^4\times S^1$ [114, 115]. If we shrink the $S^1$ circle we get the 4d theory we are interested in, we refer to [89] for a review and more references. For ${\mathcal {N}}=2$, ${{\,\mathrm{SU(2)}\,}}$ SYM the relevant setup is topological string theory on local ${{\mathbb {F}}}_0$. The mirror curve of local ${{\mathbb {F}}}_0$ is

3.20 $$\begin{aligned} \textrm{e}^x+\textrm{e}^{-x}+ {1\over m_{{{\mathbb {F}}}_0}}\textrm{e}^{y}+\textrm{e}^{-y} + {\widehat{\kappa }} = 0 \, , \end{aligned}$$

where ${\widehat{\kappa }}$ and $m_{{{\mathbb {F}}}_0}$ are the complex moduli. The quantization of this curve [41, 116] leads to the operator

3.21 $$\begin{aligned} \textrm{O}_{{{\mathbb {F}}}_0}=\textrm{e}^\textrm{x}+\textrm{e}^{-\textrm{x}}+ {1\over m_{{{\mathbb {F}}}_0}}\textrm{e}^{\textrm{y}}+\textrm{e}^{-\textrm{y}} \, , \qquad \qquad [\textrm{x},\textrm{y}]=\textrm{i}\hbar \, . \end{aligned}$$

If $\hbar , m_{{{\mathbb {F}}}_0} > 0$ the inverse operator

3.22 $$\begin{aligned} \rho _{{{\mathbb {F}}}_0}=\textrm{O}_{{{\mathbb {F}}}_0}^{-1} \end{aligned}$$

is of trace class with a positive discrete spectrum^14^ [52, 54, 119]. Hence, a natural object to consider is its Fredholm determinant

3.23 $$\begin{aligned} \det \left( 1 + {\widehat{\kappa }} \rho _{{{\mathbb {F}}}_0} \right) \, . \end{aligned}$$

The operator (3.1) can be obtained from (3.21) by implementing the usual geometric engineering limit [111, 113] where we scale

3.24 $$\begin{aligned} m_{{{\mathbb {F}}}_0} = \frac{\beta ^4 t}{4} \, , \qquad \qquad {\widehat{\kappa }} = - \frac{4}{\beta ^2 \sqrt{t}} + \frac{2 E}{\sqrt{t}} \, , \qquad \qquad \hbar = \beta \, , \end{aligned}$$

and take $\beta \rightarrow 0$. In this limit, we obtain the modified Mathieu operator (3.1),

3.25 $$\begin{aligned} \left( \textrm{O}_{{{\mathbb {F}}}_0}+{\widehat{\kappa }}\right) \psi (x)=0~\rightarrow ~ \left( \mathrm{{O}_{\textrm{Ma}}}+E\right) \psi (x) = 0 \, . \end{aligned}$$

Likewise, the Fredholm determinant becomes

3.26 $$\begin{aligned} \det \left( 1 + {\widehat{\kappa }} \rho _{{{\mathbb {F}}}_0} \right) \rightarrow \det \left( 1 + E \, \textrm{O}_{\textrm{Ma}}^{-1} \right) , \end{aligned}$$

and we have an explicit expression for this determinant via the NS functions [26, Sect. 5]

3.27 $$\begin{aligned} \det \left( 1 + E \, \textrm{O}_{\textrm{Ma}}^{-1} \right) = C(t){\sinh \left( \partial _{\sigma }F^{\textrm{NS}}(t, \sigma )\right) \over \sin \left( 2 \pi \sigma \right) }~, \end{aligned}$$

where *C*(*t*) is a normalization constant and the relation $\sigma \equiv \sigma (t, E)$ is obtained from (3.4).

The Fermi gas operator (3.5) on the other hand can be obtained from $\rho _{{{\mathbb {F}}}_0}$ by implementing a rescaled limit [48],

3.28 $$\begin{aligned} \log \left( m_{{{\mathbb {F}}}_0} \right) = 4 \pi \textrm{i} \sigma - \frac{2 \pi }{\beta } \log (\beta ^4 t) \, , {\widehat{\kappa }} = 2 \cos \left( 2 \pi \sigma \right) \, , \hbar = \frac{\left( 2 \pi \right) ^2}{\beta } \, , \end{aligned}$$

and $\beta \rightarrow 0$. This is called “the dual 4d limit” in [48]. The scaling (3.28) may seem strange at first sight, but it is a natural limit from the point of view of the TS/ST correspondence [52]. In the dual 4d limit, we have

3.29 $$\begin{aligned} \det \left( 1+{\widehat{\kappa }} \rho _{{{\mathbb {F}}}_0} \right) \rightarrow \det \left( 1+\left( {\cos (2\pi \sigma )\over 2 \pi }\right) \rho \right) , \end{aligned}$$

where $\rho$ is the operator (3.5). The determinant at the right-hand side can also be written as the Zak transform of the self-dual Nekrasov function (3.9).

Let us conclude this section by emphasizing that (3.1) has a natural interpretation directly within the four-dimensional theory, independently of the five-dimensional quantum curve. In particular, (3.1) is the canonical quantization of the four-dimensional SW curve of ${{\,\mathrm{SU(2)}\,}}$ SYM, which is related to the semiclassical limit of BPZ equations via the AGT correspondence. On the other hand for the Fermi gas operator (3.5), we do not have a parallel interpretation at the moment. It may be possible to relate this operator to some other quantization scheme of the four-dimensional SW curve. Probably a scheme similar to the one used in the context of topological recursion [120–123]^15^.

## The Seiberg–Witten Geometry from the Matrix Model

In this section, we study the ’t Hooft expansion of the matrix model (3.18) and show how the Seiberg–Witten geometry emerges from it. For this purpose it is useful to parameterize $t = \left( \Lambda / \epsilon \right) ^4$ as before in (3.11) and to introduce the potential^16^*V* such that

4.1 $$\begin{aligned} v\left( \log \left( z\right) \right) = \exp \left( - \frac{V\left( z \right) }{\epsilon }\right) \, , \qquad V \left( z \right) = 4 \Lambda \left( z + \frac{1}{z} \right) \, , \end{aligned}$$

and we take $\Lambda , \epsilon > 0$ for convenience. The matrix models (3.14) and (3.18) can then be studied in a ’t Hooft limit where

4.2 $$\begin{aligned} N \rightarrow + \infty \, , \qquad \epsilon \rightarrow 0 \, , \qquad \lambda = N \epsilon \, , \end{aligned}$$

with the defect insertion parameter *z*, the instanton counting parameter $\Lambda$, and the ’t Hooft parameter $\lambda$ all kept fixed.

This limit was implemented on the matrix model without insertions (3.14) in [48, 62]. In particular, in this limit the eigenvalues of the matrix model distribute along

4.3 $$\begin{aligned} \left[ \texttt {g}, \texttt {g}^{-1}\right] \subset {{\mathbb {R}}}_{>0} \, , \qquad 0< \texttt {g}< 1 \, , \end{aligned}$$

and the ’t Hooft parameter $\lambda$ is given by

4.4 $$\begin{aligned} \lambda =2\Lambda \frac{\textrm{K}\left( 1-\texttt {g}^4\right) \left[ 2 \textrm{E}\left( \texttt {g}^4\right) - \left( 1 - \texttt {g}^4\right) \textrm{K}\left( \texttt {g}^4\right) \right] -\pi }{ \pi \texttt {g}\textrm{K}\left( \texttt {g}^4\right) } \, , \end{aligned}$$

where $\textrm{K}$ and $\textrm{E}$ are the complete elliptic integrals of the first (C.1) and second (C.2) kind, respectively^17^. Later we will use the inversion of this relation for small $\lambda$,

4.5 $$\begin{aligned} \texttt {g}= 1-\frac{\sqrt{\lambda \over \Lambda }}{\sqrt{2}}+\frac{\lambda }{4 \Lambda }-\frac{\lambda ^{3/2}}{32 \sqrt{2}\Lambda ^{3/2}}-\frac{\lambda ^2}{64 \Lambda ^{2}}+ {\mathcal {O}}\left( \lambda ^{5/2}\right) \, . \end{aligned}$$

In the ’t Hooft limit (4.2), we have the following behavior

4.6 $$\begin{aligned} \log Z( t, N)\simeq \sum _{g\geqslant 0}\epsilon ^{2g-2} F_g\left( \Lambda , \lambda \right) \, , \end{aligned}$$

where $\simeq$ stands for asymptotic equality. The first two terms read

4.7 $$\begin{aligned}{} & {} {\textrm{d}^2 \over \textrm{d}\lambda ^2} F_0 = -2 \pi { \textrm{K}(\texttt {g}^{4})\over \textrm{K}(1-{ \texttt {g}^{4}})} \, , \end{aligned}$$

4.8 $$\begin{aligned}{} & {} F_1 =-\frac{1}{4} \log \left( \textrm{K}\left( 1-\frac{1}{\texttt {g}^4}\right) \textrm{K}\left( 1-\texttt {g}^4\right) \right) -\frac{1}{6} \log \left( \frac{1}{\texttt {g}^2}-\texttt {g}^2\right) +\textrm{constant} \, ,\nonumber \\ \end{aligned}$$

and higher-order terms can also be computed systematically [48].

Let us now consider the model with insertions (3.18). In the ’t Hooft limit (4.2), we have the following behavior [124–129]

4.9 $$\begin{aligned} \log \left( { {\sqrt{v(z)}} \Psi _N(z,t)\over Z( t, N )} \right) \simeq \sum _{n \geqslant 0} \epsilon ^{n-1} {\mathcal T}_n \left( z \right) \, . \end{aligned}$$

The leading-order term ${\mathcal T}_0$ is related to the even part of the planar resolvent^18^$\omega _+^0$ [63, eq. 3.35],

4.10 $$\begin{aligned} {\mathcal {T}}_0 \left( z \right) = - 2 \Lambda \left( z + \frac{1}{z}\right) +2 \lambda \int \limits _{\infty }^z \textrm{d}z_1 ~\omega _+^0\left( z_1 \right) \, , \end{aligned}$$

and the subleading-order term ${\mathcal T}_1$ is given by [63, 128, 129]

4.11 $$\begin{aligned} {\mathcal T}_1\left( z \right) = {2} \int \limits ^{{z}}_{\infty }\int \limits ^{{z}}_{\infty } \textrm{d}z_1 \textrm{d}z_2 \ W_{++}^0\left( z_1 , z_2 \right) \, , \end{aligned}$$

where $W_{++}^0$ is the even part of the planar two-point correlator. It can be expressed explicitly in terms of $\texttt {g}$ as [129, 63, eq. 3.43]

4.12 $$\begin{aligned}{} & {} W_{++}^0\left( z_1 , z_2 \right) \nonumber \\{} & {} \quad = \frac{ \texttt {g}^2 + \texttt {g}^{-2} - 2 \texttt {g}^{-2} \frac{\textrm{E}\left( 1-\texttt {g}^4\right) }{\textrm{K}\left( 1-\texttt {g}^4\right) } -\left( z_1^2+z_2^2\right) \left( 1-\frac{\left( \sqrt{\left( z_1^2-\texttt {g}^{-2}\right) \left( z_1^2-\texttt {g}^2\right) }-\sqrt{\left( z_2^2-\texttt {g}^{-2}\right) \left( z_2^2-\texttt {g}^2\right) }\right) ^2}{\left( z_1^2-z_2^2\right) ^2}\right) }{8 \sqrt{\left( z_1^2-\texttt {g}^{-2}\right) \left( z_1^2-\texttt {g}^2\right) } \sqrt{\left( z_2^2-\texttt {g}^{-2}\right) \left( z_2^2-\texttt {g}^2\right) }} \, . \nonumber \\ \end{aligned}$$

### The Planar Resolvent

The planar resolvent is

4.13 $$\begin{aligned} \omega ^0(z) = \lim _{N \rightarrow + \infty } {1\over N} \left\langle \sum _{n = 1}^N \left( \frac{1}{z - z_n} \right) \right\rangle \, , \end{aligned}$$

where the normalized expectation value is with respect to the matrix model without insertions $Z \left( t, N \right)$ (3.14) and the $z_n$ are the eigenvalues over which one integrates in (3.14). At large *z*, one finds

4.14 $$\begin{aligned} \omega ^0(z) = \frac{1}{z} + \frac{\langle W \rangle }{z^2} + {\mathcal {O}}\left( \frac{1}{z^3} \right) \, , \qquad \langle W\rangle = \lim _{N \rightarrow + \infty } \frac{1}{N} \left\langle \sum _{n = 1}^N z_n \right\rangle \, , \end{aligned}$$

and we refer to $\langle W\rangle$ as a Wilson loop by analogy with [130]. It is useful to split the planar resolvent in an even and an odd part,

4.15 $$\begin{aligned} \omega ^0(z)=\omega _+^0(z) +{z}\omega _-^0(z) \, , \end{aligned}$$

where $\omega _\pm ^0 \left( z \right)$ are both even in *z*.

The even part of the planar resolvent $\omega _+^0$ for the model (3.18) has the following integral form [129, eq. 4.16],

4.16 $$\begin{aligned} \lambda \omega _+^0(z) = - \frac{\textrm{i}}{2} \oint _{\mathcal {C}} \frac{\textrm{d} y }{2 \pi \textrm{i}} \left( \frac{V'(y) y}{z^2 - y^2}\right) \frac{\sqrt{ z^2 - \texttt {g}^{-2} }\sqrt{ z^2 - \texttt {g}^2 }}{\sqrt{\texttt {g}^{-2} - y^2 }\sqrt{ y^2 - \texttt {g}^2}} \, , \end{aligned}$$

where ${\mathcal {C}}$ is an anticlockwise contour around the branch cut from $\texttt {g}$ to $\texttt {g}^{-1}$, which does not include the two poles at $y = \pm z$. In the matrix model $\Psi _N \left( z , t \right)$ (3.18), we naturally have $z > 0$. However, it is useful to consider more generally $z \in {\mathbb {C}}$ from now on, and (4.16) makes indeed sense for complex values of *z* as well [129].

If $z^2 \in {\mathbb {C}} \setminus [\texttt {g}^2, \texttt {g}^{-2}]$, we can write write (4.16) as

4.17 $$\begin{aligned} \lambda \omega _+^0(z) = \left( \frac{2 \Lambda }{\pi } \right) \sqrt{ z^2 - \texttt {g}^{-2} }\sqrt{ z^2 - \texttt {g}^2 } \int \limits _\texttt {g}^{\texttt {g}^{-1}}\textrm{d} y \, \left( \frac{y - 1 / y}{z^2 - y^2} \right) \frac{1}{\sqrt{\left( \texttt {g}^{-2} - y^2 \right) \left( y^2 - \texttt {g}^2\right) }} \, . \nonumber \\ \end{aligned}$$

where we used the form of the potential given in (4.1). The integrand in (4.17) can be decomposed in partial fractions,

4.18 $$\begin{aligned}{} & {} \int \limits _b^a \textrm{d} y \left( y - \frac{1}{y} \right) \left( \frac{1}{z^2 - y^2} \right) \frac{1}{\sqrt{\left( a^2 - y^2 \right) \left( y^2 - b^2\right) }} \nonumber \\{} & {} \quad =\frac{1}{2 z^2}\left[ \left( 1 - z^2 \right) ({\mathcal {I}}(z,a,b) + {\mathcal {I}}(-z,a,b)) - 2 \, {\mathcal {I}}(0,a,b) \right] \, , \end{aligned}$$

where $0< b < a$ are any positive real numbers and we defined

4.19 $$\begin{aligned} {\mathcal {I}}\left( z,a,b\right) =\int \limits _b^a \textrm{d} y \left( \frac{1}{y-z}\right) \frac{1}{\sqrt{\left( a^2 - y^2\right) \left( y^2 - b^2\right) }} \, . \end{aligned}$$

Using [131, eqs. 256.39, 257.39], one finds for $z \notin [ b, a ]$,

4.20 $$\begin{aligned} {\mathcal {I}}\left( z,a,b\right)= & {} \left( \frac{2}{a+b}\right) \left( \frac{1}{b-z}\right) \int \limits _0^{\mathrm {K(k^2)}} \textrm{d}v \left( \frac{ 1 - k \, \textrm{sn}^2\left( v | k^2 \right) }{1- k \left( \frac{z+b}{z-b}\right) \textrm{sn}^2\left( v | k^2 \right) } \right) \nonumber \\= & {} \left( \frac{2}{a+b}\right) \left( \frac{1}{a-z}\right) \int \limits _0^{\mathrm {K(k^2)}} \textrm{d}v \left( \frac{1-\left( -k\right) \, \textrm{sn}^2\left( v | k^2 \right) }{1-k \left( \frac{a+z}{a-z}\right) \textrm{sn}^2\left( v | k^2 \right) } \right) \qquad \end{aligned}$$

where *k* is the elliptic modulus given by

4.21 $$\begin{aligned} k = \frac{a - b}{a + b} \, , \end{aligned}$$

and $\textrm{sn}\left( v | k^2 \right)$ is the Jacobi elliptic function known as the sine amplitude (C.5). From [131, eq. 340.01],

4.22 $$\begin{aligned} \int \textrm{d}v \left( \frac{1 - \alpha _1^2 \textrm{sn}^2\left( v | k^2 \right) }{1 - \alpha ^2 \textrm{sn}^2\left( v | k^2 \right) }\right) = \frac{1}{\alpha ^2} \left[ \left( \alpha ^2-\alpha _1^2\right) \Pi \left( \alpha ^2, \phi | k^2\right) +\alpha _1^2 v \right] \, , v = \textrm{F}\left( \phi |k^2\right) \, , \nonumber \\ \end{aligned}$$

where $\textrm{F}$ and $\Pi$ are the incomplete elliptic integrals of the first (C.1) and third (C.3) kind, respectively. It is useful to note that $v = 0$ corresponds to $\phi = 0$ and $v = \textrm{K}\left( k^2\right)$ corresponds to $\phi = \pi / 2$. In the end, this gives

4.23 $$\begin{aligned} \begin{aligned} {\mathcal {I}}\left( z,a,b\right)&= \left( \frac{2}{a+b}\right) \left( \frac{1}{b^2-z^2}\right) \left[ 2 b \Pi \left( \left( \frac{z + b}{z - b} \right) k | k^2 \right) + (z - b) \textrm{K} \left( k^2 \right) \right] \\&= \left( \frac{2}{a+b}\right) \left( \frac{1}{a^2-z^2}\right) \left[ 2 a \Pi \left( \left( \frac{a + z}{a - z} \right) k | k^2 \right) + (z - a) \textrm{K}\left( k^2 \right) \right] , \end{aligned} \nonumber \\ \end{aligned}$$

and hence, we have in (4.18) for $z^2 \notin \left[ b^2, a^2 \right]$

4.24 $$\begin{aligned} \begin{aligned}&{\mathcal {I}} \left( z,a,b\right) + {\mathcal {I}}\left( -z,a,b\right) \\&\quad = \left( \frac{4 b}{a+b}\right) \left( \frac{1}{b^2-z^2}\right) \left[ \Pi \left( \left( \frac{z + b}{z - b} \right) k | k^2 \right) + \Pi \left( \left( \frac{z - b}{z + b} \right) k | k^2 \right) - \textrm{K} \left( k^2 \right) \right] \\&\quad = \left( \frac{4 a}{a+b}\right) \left( \frac{1}{a^2-z^2}\right) \left[ \Pi \left( \left( \frac{a + z}{a - z} \right) k | k^2 \right) + \Pi \left( \left( \frac{a - z}{a + z} \right) k | k^2 \right) - \textrm{K}\left( k^2 \right) \right] \end{aligned} \nonumber \\ \end{aligned}$$

as well as

4.25 $$\begin{aligned} \begin{aligned} {\mathcal {I}}\left( 0,a,b\right)&= \left( \frac{2}{a+b}\right) \left( \frac{1}{b}\right) \left[ 2 \Pi \left( - k | k^2 \right) - \textrm{K} \left( k^2 \right) \right] \\&= \left( \frac{2}{a+b}\right) \left( \frac{1}{a}\right) \left[ 2 \Pi \left( k | k^2 \right) - \textrm{K} \left( k^2 \right) \right] \, . \end{aligned} \end{aligned}$$

These particular combinations of elliptic integrals can be reduced to square roots by making use of the following addition formula for $0< k < 1$ [131, eq. 117.02]^19^,

4.26 $$\begin{aligned} \Pi \left( \alpha ^2 | k^2 \right) + \Pi \left( \frac{k^2}{\alpha ^2} | k^2 \right) - \textrm{K} \left( k^2 \right) = \frac{\pi }{2} \sqrt{\frac{\alpha ^2}{\left( 1 - \alpha ^2\right) \left( \alpha ^2 - k^2\right) }} \, , \quad \alpha ^2 \in {\mathbb {C}} \setminus {\mathcal {H}}_{k^2}~, \nonumber \\ \end{aligned}$$

where $${{\mathcal {H}}_{k^2}}$$ is

4.27 $$\begin{aligned} {{\mathcal {H}}_{k^2}} =\left[ 0, k^2 \right] \cup \left[ 1, \infty \right] \, . \end{aligned}$$

Combining (4.25) and (4.26) gives

4.28 $$\begin{aligned} \begin{aligned} {\mathcal {I}}\left( 0,a,b\right)&= \left( \frac{2}{a+b}\right) \left( \frac{1}{b}\right) \left( \frac{\pi }{2}\right) \left( \frac{1}{1+k}\right) = \left( \frac{\pi }{2}\right) \left( \frac{1}{a b}\right) \\&= \left( \frac{2}{a+b}\right) \left( \frac{1}{a}\right) \left( \frac{\pi }{2}\right) \left( \frac{1}{1-k}\right) = \left( \frac{\pi }{2}\right) \left( \frac{1}{a b}\right) \, , \end{aligned} \end{aligned}$$

and the combination of (4.24) and (4.26) leads to

4.29 $$\begin{aligned} \begin{aligned} {\mathcal {I}}&\left( z,a,b\right) + {\mathcal {I}}\left( -z,a,b\right) \\&\quad = \left( \frac{4 b}{a+b}\right) \left( \frac{1}{b^2-z^2}\right) \left[ \frac{\pi }{4} \left( \frac{a+b}{b}\right) \sqrt{\frac{b^2-z^2}{a^2-z^2}} \right] = -\frac{\pi }{\sqrt{z^2 - a^2}\sqrt{z^2 - b^2}} \\&\quad = \left( \frac{4 a}{a+b}\right) \left( \frac{1}{a^2-z^2}\right) \left[ \frac{\pi }{4} \left( \frac{a+b}{a}\right) \sqrt{\frac{a^2-z^2}{b^2-z^2}} \right] = -\frac{\pi }{\sqrt{z^2 - a^2}\sqrt{z^2 - b^2}} \end{aligned} \nonumber \\ \end{aligned}$$

where $$ z^2 \in {\mathbb {C}} \setminus \left[ b^2, a^2\right] $$.

Taking $a^{-1} = b = \texttt {g}$ and using everything above, we finally find for the even planar resolvent

4.30 $$\begin{aligned} \boxed { \lambda \omega _+^0(z) = \Lambda \left( \left( 1 - \frac{1}{z^2} \right) - \frac{\sqrt{z^2-\texttt {g}^{2}}\sqrt{z^2-\texttt {g}^{-2}}}{z^2} \right) \, . } \end{aligned}$$

Even though we derive (4.30) for $z^2 \in {{\mathbb {C}}}\setminus [\texttt {g}^2, \texttt {g}^{-2}]$, one can verify that (4.30) holds on the whole complex plane. As a consistency check, we compared the analytical result (4.30) against the numerical evaluation of (4.16) and found perfect agreement. One can also see that (4.30) has the correct asymptotic behavior,

4.31 $$\begin{aligned} \begin{aligned} \omega _+^0(z) =&\ {\mathcal {O}}\left( 1\right) \, , \quad{} & {} \text {for} \ z \rightarrow 0 \, , \\ \omega _+^0(z) =&\ {\mathcal {O}}\left( z^{-2} \right) \, , \quad{} & {} \text {for} \ z \rightarrow \infty \, . \end{aligned} \end{aligned}$$

In addition, from the coefficient of the $z^{-2}$-term in the $\ z \rightarrow \infty$ expansion we get a closed-form expression for the Wilson loop (4.14),

4.32 $$\begin{aligned} \langle W \rangle =\frac{\left( \texttt {g}^2-1\right) ^2 \Lambda }{2 \texttt {g}^2 \lambda } \, . \end{aligned}$$

Using (4.5), we obtain

4.33 $$\begin{aligned} \frac{\left( \texttt {g}^2-1\right) ^2 \Lambda }{2 \texttt {g}^2 \lambda }=1 +\frac{\lambda }{16 \Lambda }-\frac{\lambda ^2}{256 \Lambda ^2}+\frac{5 \lambda ^3}{8192 \Lambda ^3}-\frac{33 \lambda ^4}{262144 \Lambda ^4}+{\mathcal {O}}\left( \lambda ^{5}\right) .\nonumber \\ \end{aligned}$$

We cross-checked (4.33) by expanding the matrix model around its Gaussian point, similar to what was done in [62, app. B].

Using (4.30) gives for the leading order ${\mathcal {T}}_0$ (4.10) of the matrix model (4.9) in the ’t Hooft limit (4.2)

4.34 $$\begin{aligned} \partial _z {\mathcal {T}}_0 \left( z \right) = - 2 \Lambda \left( 1 - \frac{1}{z^2} \right) + 2 \lambda \omega _+^0(z) = - 2\Lambda \frac{\sqrt{z^2-\texttt {g}^{2}}\sqrt{z^2-\texttt {g}^{-2}}}{z^2}~.\nonumber \\ \end{aligned}$$

An important point of (4.34) is that the Seiberg–Witten curve of ${\mathcal {N}}=2$, ${{\,\mathrm{SU(2)}\,}}$ SYM,

4.35 $$\begin{aligned} y^2(z)= - 4 \Lambda ^2 \left( z^2+z^{-2}\right) + {u} \, , \end{aligned}$$

emerges in the planar limit, provided we identify the following quadratic differentials

4.36 $$\begin{aligned} \left[ \partial _z {\mathcal {T}}_0 \left( z\right) \textrm{d} z \right] ^2 = - \left[ \frac{y \left( z\right) }{z} \textrm{d} z \right] ^2, \end{aligned}$$

and at the same time relate $\texttt {g}$ to *u* by

4.37 $$\begin{aligned} \texttt {g}^2+\texttt {g}^{-2}={u\over 4 \Lambda ^2} \, , \quad \texttt {g}^{\pm 2} = \left( \frac{u}{8 \Lambda ^2}\right) \mp \sqrt{\left( \frac{u}{8 \Lambda ^2}\right) ^2 - 1} \, . \end{aligned}$$

In Eqs. (4.35) and (4.37), *u* denotes the vacuum expectation value of the scalar in the vector multiplet of ${\mathcal {N}}=2$, ${{\,\mathrm{SU(2)}\,}}$ SYM.

### The Planar Two-Point Function

In the previous section, we showed that the Seiberg–Witten curve (4.35) naturally emerges when considering the planar resolvent. Here we will see that the Bergman kernel emerges similarly when considering the even part of the planar two-point function. We will see later that this characterizes the annulus amplitude in the surface defect. The Bergman kernel is defined as [78]

4.38 $$\begin{aligned} B_{q_1, q_2, q_3}(z_1,z_2)= {1\over 2(z_1-z_2)^2} \left( {2 f(z_1, z_2) + G_{q_1, q_2, q_3}\left( k^2\right) (z_1-z_2)^2\over 2 \sqrt{\sigma (z_1) \sigma (z_2)}}+1\right) \, , \nonumber \\ \end{aligned}$$

with

4.39 $$\begin{aligned}{} & {} f(z_1, z_2)=\frac{u \left( z_1^2+4 z_2 z_1+z_2^2\right) }{24 \Lambda ^2}+\frac{1}{2} \left( -z_1-z_2\right) -\frac{1}{2} z_1 z_2 \left( z_1+z_2\right) \, , \nonumber \\ \end{aligned}$$

4.40 $$\begin{aligned}{} & {} G_{q_1, q_2, q_3}\left( k^2\right) = \left( q_1-q_3\right) \left[ \frac{ \textrm{E}\left( k^2\right) }{\textrm{K}\left( k^2\right) }\right] + q_3 - \frac{u}{12\Lambda ^2} \, , \quad k^2 = \frac{q_1-q_2}{q_1-q_3} \, , \nonumber \\ \end{aligned}$$

and where $q_i$ are the branch points of $\sigma (z)=-z \left( z^2 - \left( u/ 4 \Lambda ^2\right) z + 1 \right)$,

4.41 $$\begin{aligned} q_i \in \left\{ 0, \left( \frac{u}{8 \Lambda ^2}\right) \mp \sqrt{\left( \frac{u}{8 \Lambda ^2}\right) ^2 - 1}\right\} = \left\{ 0, \texttt {g}^2, \texttt {g}^{-2} \right\} \, . \end{aligned}$$

The choice of the order fixes the choice of frame. What we find is that the relevant order here is

4.42 $$\begin{aligned}{} & {} q_3 = 0 \, , \quad q_{2} = \left( \frac{u}{8 \Lambda ^2}\right) - \sqrt{\left( \frac{u}{8 \Lambda ^2}\right) ^2 - 1} = \texttt {g}^2 \, , \nonumber \\{} & {} \quad q_1 = \left( \frac{u}{8 \Lambda ^2}\right) + \sqrt{\left( \frac{u}{8 \Lambda ^2}\right) ^2 - 1}= \texttt {g}^{-2} \, . \end{aligned}$$

As we will discuss later this choice makes contact with the magnetic frame. One can check that the even part of the planar two-point function (4.12) is related to the Bergman kernel (4.38) by

4.43 $$\begin{aligned} W_{++}^0\left( z_1, z_2\right) = - z_1 z_2 B_{{q}_1, {q}_2, {q}_3}\left( z_1^2, z_2^2\right) - \frac{1}{4 \left( z_1 + z_2 \right) ^2} \, , \end{aligned}$$

Hence, the subleading order ${\mathcal {T}}_1$ (4.11) of the matrix model (4.9) in the ’t Hooft limit (4.2) becomes

4.44 $$\begin{aligned} \boxed {{\mathcal {T}}_1 \left( z\right) =2 \int \limits _{\infty }^z \int \limits _{\infty }^z \textrm{d}z_1 \textrm{d}z_2 \left( - z_1 z_2 B_{{q}_1, {q}_2, {q}_3}\left( z_1^2, z_2^2\right) - \frac{1}{4 \left( z_1 + z_2 \right) ^2}\right) } \; . \end{aligned}$$

## Testing the $\epsilon$ Expansion for the Type I Defect

From the perspective of the B-model, the partition functions of open and closed topological strings can be defined as objects associated with an algebraic curve, and thus, they depend on a choice of frame, namely a choice of a symplectic basis for the homology of the algebraic curve. The transformation properties of the closed string partition function under a change of frame can be derived from the observation that such a partition function behaves like a wavefunction [132]. Consequently, the genus *g* free energies behave as almost modular forms under a change of frame [133]. The wavefunction behavior was generalized to the open topological string sector in [85].

Recall that the partition functions of the four-dimensional gauge theories under consideration are derived from the topological string partition functions via the geometric engineering construction [111–113]. As a result, the same transformation properties hold.

At the level of terminology, the large radius frame in topological string theory is mapped to the electric frame in the four-dimensional theory. In this frame, the ${\mathcal {A}}$ cycle and the corresponding A period on the SW curve (4.35) are chosen to be

5.1 $$\begin{aligned} \Pi _A= {1\over 2 \pi \textrm{i}} \oint _{{\mathcal {A}}} {y(z)\over z} \textrm{d}z = {1\over \textrm{i} \pi }\int \limits _{-\textrm{i}\pi }^{\textrm{i}\pi } {y(z)\over z} \textrm{d}z = \frac{2 \sqrt{8 \Lambda ^2+u}~ {\mathrm E}\left( \frac{16 \Lambda ^2}{8 \Lambda ^2+u}\right) }{\pi } \, , \end{aligned}$$

where *y*(*z*) is given in (4.35) and $\textrm{E}$ is the complete elliptic integral of second kind (C.2). We usually denote $a \equiv \Pi _A$. Likewise the ${\mathcal {B}}$ cycle and the corresponding period are

5.2 $$\begin{aligned} \Pi _B= & {} {1\over 2 \pi \textrm{i}} \oint _{{\mathcal {B}}} {y(z)\over z} \textrm{d}z ={1\over \textrm{i}\pi }\int \limits _{\texttt {g}}^{\texttt {g}^{-1}} {y(z)\over z} \textrm{d}z \nonumber \\= & {} { \sqrt{8 \Lambda ^2+u} \over \textrm{i}\pi } \left( {\mathrm K}\left( \frac{2 u}{8 \Lambda ^2+u}-1\right) -{\mathrm E}\left( \frac{2 u}{8 \Lambda ^2+u}-1\right) \right) \end{aligned}$$

where $\textrm{K}$ is the complete elliptic integral of the first kind (C.1). The $\texttt {g}^{\pm 1}$ are roots of the SW curve, $y\left( \texttt {g}^{\pm 1}\right) = 0$, and are given in (4.37). We usually denote $a_D\equiv \textrm{i}\Pi _B$.

On the other hand, the conifold frame in topological strings corresponds to the magnetic frame in the four-dimensional theory. This frame is related to the electric frame by an S-duality which exchanges the ${\mathcal {A}}$- and ${\mathcal {B}}$-cycles. For the ${{\,\mathrm{SU(2)}\,}}$ SYM that we study in this paper, the transformation properties of the partition function under a change of frame were studied in [102]. The $\epsilon$ expansion of (3.15) leads exactly to such transformations, as we discuss below.

### The Electric Frame

We consider a type I defect in the self-dual phase of the $\Omega$-background ($\epsilon _1=-\epsilon _2=\epsilon$), and we denote the partition function of this surface defect by

5.3 $$\begin{aligned} Z^{\textrm{I}}(z, t, \sigma ) \end{aligned}$$

if we are in the electric frame. As pointed out in [68], based on [134, 135], we can compute these defects via the Eynard–Orantin topological recursion [84]. More precisely, we have

5.4 $$\begin{aligned} \log \left( Z^{\textrm{I}}(z, t, \sigma ) \right) \simeq \sum _{g\geqslant 0}\sum _{h\geqslant 1}{\epsilon ^{2g-2+h}}\int ^z_{\infty } \cdots \int ^z_{\infty } W_{g,h}(z_1, \ldots , z_h) \ \textrm{d}z_1 \cdots \textrm{d}z_h \nonumber \\ \end{aligned}$$

where $W_{g,h}(z_1,...z_h)\textrm{d}z_1\dots \textrm{d}z_h$ is an infinite sequence of meromorphic differentials constructed via topological recursion [84] and whose starting point is the underlying SW geometry (4.35). Note that we are implicitly using the dictionary (3.11) and the SW relation (5.1) to express $\sigma = \textrm{i} a / 2 \epsilon$ as a function of the SW parameter *u*.

For the so-called disk amplitude ${W}_{0,1}$, we have^20^

5.5 $$\begin{aligned} {W}_{0,1}(z) \ \textrm{d}z = - \frac{2 \Lambda }{z} \sqrt{ \left( z^2 + \frac{1}{z^{2}}\right) -{u\over 4 \Lambda ^2}} \ \textrm{d}z \, , \end{aligned}$$

and we note

5.6 $$\begin{aligned} {\mathcal {W}}_{0}^{\textrm{I}}(z, \Lambda , u )=\int ^z_{\infty } {W}_{0,1}( z_1 ) \ \textrm{d}z_1 \, . \end{aligned}$$

The annulus amplitude ${W}_{0,2}$ is given by

5.7 $$\begin{aligned} {W}_{0,2}(z_1, z_2) \ \textrm{d}z_1\textrm{d}z_2 = - 2 z_1 z_2 \left( B_{{\widetilde{q}}_1, {\widetilde{q}}_2, \widetilde{q}_3}(z_1^2,z_2^2)- {1\over (z_1^2- z_2^2)^2}\right) \ \textrm{d}z_1\textrm{d}z_2 \, , \nonumber \\ \end{aligned}$$

where $B_{{\widetilde{q}}_1, {\widetilde{q}}_2, {\widetilde{q}}_3}$ is defined as in (4.38), but the choice of $q_i$’s is different. Here we have

5.8 $$\begin{aligned}{} & {} \widetilde{q_1} = 0 \, , \quad \widetilde{q_2} = \left( \frac{u}{8 \Lambda ^2}\right) - \sqrt{\left( \frac{u}{8 \Lambda ^2}\right) ^2 - 1} = \texttt {g}^2 \, , \nonumber \\{} & {} \quad \widetilde{q_3} = \left( \frac{u}{8 \Lambda ^2}\right) + \sqrt{\left( \frac{u}{8 \Lambda ^2}\right) ^2 - 1}= \texttt {g}^{-2} \, , \end{aligned}$$

so that $\widetilde{q_1} = q_3$, $\widetilde{q_2} = q_2$ and $\widetilde{q_3} = q_1$ (4.42). We denote

5.9 $$\begin{aligned} {\mathcal {W}}_1^{\textrm{I}}(z, \Lambda , u)= \int ^z_{\infty }\int ^z_{\infty } {W}_{0,2}(z_1, z_2) \ \textrm{d}z_1\textrm{d}z_2 \, . \end{aligned}$$

Hence, to subleading order (5.4) reads

5.10 $$\begin{aligned} \log \left( Z^{\textrm{I}}(z, t, \sigma ) \right) = {{1\over \epsilon } {\mathcal {W}}_0^{\textrm{I}}(z, \Lambda , u )+{\mathcal {W}}_1^{\textrm{I}}(z, \Lambda , u)+{\mathcal {O}}(\epsilon )} \, . \end{aligned}$$

Given the spectral curve (4.35) with $W_{0,1}$ and $W_{0,2} \,$, higher-order terms in the $\epsilon$ expansion (5.4) can be computed recursively by using the topological recursion [84].

### The Magnetic Frame

We conjecture that the matrix model (3.18) computes the type I surface defect (5.3) in the magnetic frame. In this section, we test this proposal in the ’t Hooft expansion (4.2).

#### The Partition Function Without Defects

It is useful to start by reviewing the change of frame in the partition function without defect, which follows from the $\epsilon$ expansion of (3.15). Using the dictionary (3.11), the $\epsilon$ expansion of the Nekrasov function reads

5.11 $$\begin{aligned} \log \left( Z^{\textrm{Nek}}( t, \sigma )\right) \simeq \sum _{g\geqslant 0}\epsilon ^{2g-2}{\mathcal {F}}_g (\Lambda , a) \, , \end{aligned}$$

where ${\mathcal {F}}_g$ are the genus *g* free energies of ${{\,\mathrm{SU(2)}\,}}$ SYM in the electric frame. Thanks to (3.15), we can relate (5.11) to the ’t Hooft expansion of the matrix model (3.14),

5.12 $$\begin{aligned} \log \left( Z(t, N) \right) \simeq \sum _{g\geqslant 0}\epsilon ^{2g-2} F_g( \Lambda , \lambda ) \, . \end{aligned}$$

It was found in [48] that the $F_g$’s in (5.12) are the SYM free energies in the magnetic frame. More precisely,

5.13 $$\begin{aligned} \textrm{e}^{\sum _{g\geqslant 0}\epsilon ^{2g-2} F_g(\Lambda , \lambda )}\sim \int \limits _{\textrm{i}{{\mathbb {R}}}} {\textrm{d}a} \ \textrm{e}^{- \pi a N / \epsilon } \ \textrm{e}^{\sum _{g\geqslant 0}\epsilon ^{2g-2}{\mathcal {F}}_g (\Lambda , a)} \, , \end{aligned}$$

where $\sim$ indicates a proportionality between two (divergent) series^21^. The integral on the right-hand side of (5.13) should be understood as a saddle point expansion. This saddle point expansion characterizes the change of frame in SW theory and topological string [132], and it has a direct interpretation from the point of view of modular transformations [133]. It allows us to make the transition from the weak coupling electric frame, where the Nekrasov function (3.10) is defined, to the strong coupling magnetic frame, where the matrix model (3.14) naturally emerges.

By writing the saddle point expansion on the right-hand side of (5.13) explicitly, we get

5.14 $$\begin{aligned} \int \limits _{\textrm{i}{{\mathbb {R}}}} {\textrm{d}a} \ \textrm{e}^{- \pi a N / \epsilon } \ \textrm{e}^{\sum _{g\geqslant 0}\epsilon ^{2g-2}{\mathcal {F}}_g (\Lambda , a)} = \exp \left[ \frac{1}{\epsilon ^2} \left( {{\mathcal {F}}}_0 \left( \Lambda , a(\lambda ) \right) - {\pi a(\lambda ) \lambda } \right) +{\mathcal {O}}(1)\right] \, , \nonumber \\ \end{aligned}$$

where $\lambda = N \epsilon$ and $a(\lambda )$ is determined by the saddle point equation

5.15 $$\begin{aligned} \partial _a {{\mathcal {F}}}_0 \left( \Lambda , a \right) = \pi \lambda \, . \end{aligned}$$

By using (3.10) and the dictionary (3.11), we get

5.16 $$\begin{aligned} \partial _a {{\mathcal {F}}}_0( \Lambda , a )= & {} 2 a (\log (a)-\log (\Lambda )-1) +\frac{4 \Lambda ^4}{a^3}+\frac{30 \Lambda ^8}{a^7}\nonumber \\{} & {} +\frac{480 \Lambda ^{12}}{a^{11}}+\frac{10283 \Lambda ^{16}}{a^{15}}+\frac{1287648 \Lambda ^{20}}{5 a^{19}} + {\mathcal {O}} \left( \Lambda ^{24}\right) \, , \end{aligned}$$

and if we make use of (4.37) together with the classical Matone relation,

5.17 $$\begin{aligned} u= & {} -\Lambda \partial _{\Lambda } {\mathcal {F}}_0 \left( \Lambda , a \right) = a^2+\frac{8 \Lambda ^4}{a^2}+\frac{40 \Lambda ^8}{a^6}+\frac{576 \Lambda ^{12}}{a^{10}} \nonumber \\{} & {} + \frac{11 752 \Lambda ^{16}}{a^{14}} + \frac{286 144 \Lambda ^{20}}{a^{18}} +{\mathcal {O}}\left( \Lambda ^{24}\right) \, , \end{aligned}$$

we find that $\lambda$ in (5.15) agrees with (4.4) as it should. The matching between the two sides of (5.13) was discussed in [48]. We also note that the classical Matone relation (5.17) can be inverted and one finds the usual expression for the *A*-period of the SW curve (4.35) given in (5.1). Likewise $\partial _a {\mathcal {F}}_0$ is identified with the *B*-period of the SW curve^22^

5.18 $$\begin{aligned} a_D= {\pi ^{-1}} \partial _a {\mathcal {F}}_0 \left( \Lambda , a \right) , \end{aligned}$$

where $a_D$ is given in (5.2).

#### The Partition Function with Defects

We are interested in extending the analysis to the ’t Hooft expansion (4.9) of the matrix model with insertion $\Psi _N(z,t)$ (3.18). More precisely, we claim that $\Psi _N(z,t)$ gives the self-dual type I surface defect (5.3) in the magnetic frame. As we reviewed above, the change of frame for the partition function is encoded in an integral transform (5.13). As first shown in [85], this is still the case if one considers the partition function in the presence of surface defects which are engineered via the open topological string partition function, see also [80].

At the level of the $\epsilon$ expansion, our conjecture reads

5.19 $$\begin{aligned}{} & {} \exp \left[ \sum _{g\geqslant 0}\epsilon ^{2g-2} F_g( \Lambda , \lambda ) + \sum _{n \geqslant 0} \epsilon ^{n-1} {\mathcal T}_n(z) \right] \nonumber \\{} & {} \quad \sim \int \limits _{\textrm{i}{{\mathbb {R}}}} {\textrm{d}a} \ \textrm{e}^{- \pi a N / \epsilon } \ \textrm{e}^{\sum _{g\geqslant 0}\epsilon ^{2g-2}{\mathcal {F}}_g ( \Lambda , a )} \textrm{e}^{\sum _{g\geqslant 0}\sum _{h\geqslant 1}{\epsilon ^{2g-2+h}}\int ^z_\infty \cdots \int ^z_\infty W_{g,h}(z_1, \ldots , z_h)\textrm{d}z_1 \cdots \textrm{d}z_h} \, , \nonumber \\ \end{aligned}$$

where $W_{g,h}(z_1, \ldots ,z_h)\textrm{d}z_1 \cdots \textrm{d}z_h$ are the electric differentials appearing in the topological recursion setup (5.4), whereas ${\mathcal T}_n$ are the magnetic matrix model coefficients appearing in (4.9),

5.20 $$\begin{aligned} \left( { {\sqrt{v(z)}}\Psi _N(z,t)\over Z( t, N )} \right) \simeq \exp \left[ \sum _{n \geqslant 0} \epsilon ^{n-1} {\mathcal T}_n(z)\right] \, . \end{aligned}$$

Parallel to (5.13), the integral on the right-hand side of (5.19) should be understood as a saddle point expansion which characterizes the change of frame. Equation (5.19) reads to subleading order in $\epsilon$

5.21 $$\begin{aligned}{} & {} \epsilon ^{-1} {\mathcal {T}}_0(z)+{\mathcal {T}}_1(z) + {\mathcal {O}}\left( \epsilon \right) = \epsilon ^{-1} {\mathcal W}_{0}^{\textrm{I}}\left( z, a(\lambda )\right) \nonumber \\{} & {} \quad - \frac{\left[ \partial _a{{\mathcal {W}}}_{0}^{\textrm{I}}\left( z, a(\lambda )\right) \right] ^2}{2 \partial _a^2 {\mathcal {F}}_{0}\left( a(\lambda )\right) }+{{\mathcal {W}}}_{1}^{\textrm{I}}( z, a(\lambda ) ) + {\mathcal {O}}\left( \epsilon \right) \, , \end{aligned}$$

where *a* and $\lambda$ are again related by the saddle point Eq. (5.15). In (5.21), we already used (5.13) and (5.14) to get rid of terms involving only the free energies $F_g$ and ${\mathcal {F}}_g$. We show below that the equality in (5.21) indeed holds order by order in $\epsilon$.

At the leading order $\epsilon ^{-1}$, the matching on the two sides of (5.21) follows directly from (4.34) and (5.5). For the subleading order $\epsilon ^0$, we first note that the Bergman kernel entering in ${\mathcal {T}}_1$ (4.44), can be written as

5.22 $$\begin{aligned}{} & {} B_{ q_1, q_2, q_3}(z_1,z_2) \nonumber \\{} & {} \quad =B_{{\widetilde{q}}_1, {\widetilde{q}}_2, \widetilde{q}_3}(z_1,z_2)+\frac{\pi \left[ {z_1 z_2 \left( \texttt {g}^4 z_1-\texttt {g}^2 \left( z_1^2+1\right) +z_1\right) \left( \texttt {g}^4 z_2-\texttt {g}^2 \left( z_2^2+1\right) +z_2\right) } \right] ^{-1/2}}{8 \, \textrm{K}\left( \texttt {g}^4\right) \textrm{K}\left( 1-\texttt {g}^4\right) } \nonumber \\ \end{aligned}$$

where we used (4.37) and (5.8). Hence, we can rewrite (4.44),

5.23 $$\begin{aligned} {\mathcal {T}}_{1} \left( z \right)= & {} 2 \int \limits _{\infty }^{z} \int \limits _{\infty }^{z} \left( - z_{1} z_{2} B_{{q}_{1} {q}_{2} {q}_{3}}\left( z_{1}^{2}, z_{2}^{2}\right) - \frac{1}{4 \left( z_{1} + z_{2} \right) ^{2}}\right) \textrm{d}z_{1} \textrm{d}z_{2}\nonumber \\= & {} {\mathcal {W}}_1^{\textrm{I}} \left( z \right) -\int ^z_{\infty }\int ^z_{\infty }\left( \frac{\pi \texttt {g}^4}{16 \left( \texttt {g}^4-1\right) ^2 {\Lambda }^2}\frac{ \partial _{\texttt {g}}\partial _{z_{1}}{\mathcal {T}}_0(z_1)\partial _{\texttt {g}}\partial _{z_{2}}{\mathcal {T}}_{0}(z_{2})}{ \textrm{K}\left( \texttt {g}^4\right) \textrm{K}\left( 1-\texttt {g}^4\right) }\right) \textrm{d}z_{1}\textrm{d}z_{2} \, ,\nonumber \\ \end{aligned}$$

which leads then to

5.24 $$\begin{aligned} {\mathcal {T}}_1 \left( z \right)= & {} {\mathcal {W}}_1^{\textrm{I}} \left( z \right) -\frac{\pi \texttt {g}^4}{16 \left( \texttt {g}^4-1 \right) ^2 {\Lambda }^2} \frac{\left( \partial _{\texttt {g}}{\mathcal {T}}_0(z)\right) ^2}{ \textrm{K}\left( \texttt {g}^4\right) \textrm{K}\left( 1-\texttt {g}^4\right) } \nonumber \\= & {} \, {\mathcal {W}}_1^{\textrm{I}} \left( z \right) -\left( \partial _{a}{\mathcal {T}}_0(z)\right) ^2\frac{\textrm{K}\left( \frac{4 \texttt {g}^2}{\left( \texttt {g}^2+1\right) ^2}\right) ^2}{ \pi \left( \texttt {g}^2+1\right) ^2 \textrm{K}\left( \texttt {g}^4\right) \textrm{K}\left( 1-\texttt {g}^4\right) } \, , \end{aligned}$$

where we used (5.1). From (5.18), we have

5.25 $$\begin{aligned} \partial _a^2 {\mathcal {F}}_0\left( a\right) = \frac{\pi {\mathrm K}\left( \frac{2 u}{8 \Lambda ^2+u}-1\right) }{{\mathrm K}\left( \frac{16 \Lambda ^2}{8 \Lambda ^2+u}\right) } \, , \end{aligned}$$

and by combining (5.25) with the identity

5.26 $$\begin{aligned} \frac{\textrm{K}\left( \frac{4 \texttt {g}^2}{\left( \texttt {g}^2+1\right) ^2}\right) }{ \left( \texttt {g}^2+1\right) ^2 \textrm{K}\left( \texttt {g}^4\right) \textrm{K}\left( 1-\texttt {g}^4\right) }=\frac{1}{2 \textrm{K}\left( \frac{\left( \texttt {g}^2-1\right) ^2}{\left( \texttt {g}^2+1\right) ^2}\right) } \end{aligned}$$

we find

5.27 $$\begin{aligned} \boxed {{\mathcal {T}}_1 \left( z \right) = {\mathcal {W}}_1^{\textrm{I}} \left( z \right) - { \left( \partial _{a}{\mathcal {T}}_0(z)\right) ^2 \over 2 \partial _a^2 {\mathcal {F}}_0}} \, , \end{aligned}$$

which is precisely what we wanted to prove.

To summarize, we have tested (5.19) at leading and subleading order^23^ in $\epsilon$. The matching of higher orders can be inferred from the application of topological recursion. On the canonical defect side, the fact that higher orders in (5.3) satisfy the topological recursion was conjectured in [68], based on [134, 135] which was recently demonstrated in [136]. On the matrix model side instead, the inclusion of topological recursion in our matrix model can be derived from [84, 137, 138]. Our computation above shows that the initial data for such recursion are the same on both sides; therefore, matching at all orders is also expected.

## Matrix Models, Eigenfunctions, and the Type II Defect

In this section, we consider the Fourier transform of the matrix model with insertion $\Psi _N\left( \textrm{e}^x, t\right)$ (3.18). The corresponding defect in four-dimensional, ${\mathcal {N}} = 2$, ${{\,\mathrm{SU(2)}\,}}$ SYM can be geometrically engineered using the open topological string partition function of local ${{\mathbb {F}}}_0$, where we insert a D-brane on the external leg, see Appendix A. The partition function of the resulting type II defect in the self-dual phase of the $\Omega$-background is

6.1 $$\begin{aligned} \begin{aligned} Z^{\textrm{II}}(q, t, \sigma )&= \exp \left( \frac{\textrm{i}}{2} q \log \left( t \right) \right) \Gamma \left( - \textrm{i} q-\sigma +\frac{1}{2}\right) \Gamma \left( - \textrm{i} q + \sigma + \frac{1}{2}\right) Z_\textrm{inst}^{\textrm{II}}(q, t, \sigma ) \, , \\ Z_{\textrm{inst}}^\text {II}\left( q, t, \sigma \right)&= 1 - \left[ \frac{\widetilde{q}}{2 \sigma ^2 \left( \widetilde{q}^2 - \sigma ^2 \right) }\right] t \\&\quad + \left[ \frac{\widetilde{q} \left( \widetilde{q} + 1 \right) ^2 - \widetilde{q} \left( 10 \widetilde{q}^2 + 19 \widetilde{q} + 10 \right) \sigma ^2 + \left( 8 \widetilde{q}^2 + 30 \widetilde{q} + 9 \right) \sigma ^4}{4 \sigma ^4 \left( 4 \sigma ^2 - 1 \right) ^2 \left( \widetilde{q}^2 - \sigma ^2\right) \left( \left( \widetilde{q} + 1 \right) ^2 - \sigma ^2\right) } \right] t^2 +{\mathcal {O}}\left( t^3\right) \, , \end{aligned}\nonumber \\ \end{aligned}$$

where we defined for the sake of readability $\widetilde{q} = \textrm{i} q + 1 / 2$. The variables $q, t, \sigma$ can be expressed in terms of $y, \Lambda , a$ and $\epsilon$ as in (2.4). The relation between $Z^{\textrm{II}}$ and the matrix model (3.18) reads^24^

6.2 $$\begin{aligned}{} & {} - \textrm{i} \, 2^{1/12} \textrm{e}^{3\zeta '(-1)}t^{-1/16}\textrm{e}^{4 \sqrt{t}} \int \limits _{ {{\mathbb {R}}}+\textrm{i}\sigma _*}{\textrm{d}\sigma } {\tan \left( 2 \pi \sigma \right) \over \left( 2 \cos (2 \pi \sigma )\right) ^N} Z^\textrm{Nek}(t, \sigma ) \left( \sum _{s}Z^{\textrm{II}}_{s}(q, t, \sigma )\right) \nonumber \\{} & {} \quad = {2\sqrt{\pi }{ t^{1/8} } \over (4 \pi )^N}\int \limits _{\mathbb {R}} {\textrm{d}x} \, \textrm{e}^{-\textrm{i}{2} q x} \textrm{e}^{-4 t^{1/4} \cosh \left( x \right) + \frac{x}{2}} \Psi _N(\textrm{e}^{{x}}, t) \, , \end{aligned}$$

where $\sigma _*$ is chosen such that $0< \sigma _* < |\textrm{Re}(q)|$ if $\textrm{Re}\left( q \right) \ne 0$, and simply $\sigma _* > 0$ if $\textrm{Re}\left( q \right) = 0$. This guarantees that the integral on the left-hand side does not hit the poles of the integrand. The sum over *s* can be seen as a sum over saddle points of the integral over *x*. We find that

6.3 $$\begin{aligned} \left( \sum _{s}Z^{\textrm{II}}_{s}(q, t, \sigma )\right) =Z^{\textrm{II}}\left( q, t, \sigma \right) + \left( -1\right) ^N Z^{\textrm{II}}\left( -q-{\textrm{i}\over 2}, t, \sigma \right) \, . \end{aligned}$$

It is convenient to introduce the total partition function as

6.4 $$\begin{aligned} Z^{\textrm{II}}_{\textrm{tot}}( q, t, \sigma )= Z^{\textrm{Nek}}(t, \sigma )Z^{\textrm{II}}(q, t, \sigma ) \, , \end{aligned}$$

so that (6.2) can be written in a compact form as

6.5 $$\begin{aligned} \boxed {\begin{aligned}&\int \limits _{ {{\mathbb {R}}}+\textrm{i}\sigma _*}{\textrm{d}\sigma } {\tan \left( 2 \pi \sigma \right) \over \left( 2 \cos (2 \pi \sigma )\right) ^N} \left( Z^{\textrm{II}}_{\textrm{tot}}(q, t, \sigma ) + (-1)^N Z^\textrm{II}_{\textrm{tot}}\left( -q-{\textrm{i}\over 2}, t, \sigma \right) \right) \\&= \textrm{i}{2^{11/12}\sqrt{\pi }{ t^{3/16} }\over \textrm{e}^{3\zeta '(-1)}\textrm{e}^{4 \sqrt{t}}(4\pi )^N}\int \limits _{\mathbb {R}} {\textrm{d}x} \, \textrm{e}^{-\textrm{i}{2} q x} \textrm{e}^{- 4 t^{1/4} \cosh x+ {x\over 2}} \Psi _N(\textrm{e}^{{x}}, t) \, . \end{aligned}}\quad \end{aligned}$$

This can equivalently be written as

6.6 $$\begin{aligned} \begin{aligned}&\sum _{k \in {{\mathbb {Z}}}} \left( Z^{\textrm{II}}_{\textrm{tot}}(q, t, \sigma + k )+Z^{\textrm{II}}_\textrm{tot}\left( -q-{\textrm{i}\over 2}, t, \sigma + k + {1\over 2} \right) \right) \\&= { 2^{11/12}\sqrt{\pi }{ t^{3/16} }\over \textrm{e}^{ 3\zeta '(-1)}\textrm{e}^{4 \sqrt{t}} }\int \limits _{\mathbb {R}} {\textrm{d}x} \, \textrm{e}^{-\textrm{i}{2} q x} \textrm{e}^{- 4 t^{1/4} \cosh x+ {x\over 2}} \left[ \sum _{N\geqslant 0} \Psi _N(\textrm{e}^{{x}}, t) \left( \frac{\cos (2\pi \sigma )}{2\pi }\right) ^N \right] \, .\end{aligned} \nonumber \\ \end{aligned}$$

One can use an argument based on the Fourier series to get from (6.5) to (6.6), while the other direction uses Cauchy’s residue theorem. See Appendix B for details. By inverting the Fourier transform (6.6), we have

6.7 $$\begin{aligned} \boxed {\begin{aligned}&\int \limits _{{{\mathbb {R}}}} \textrm{d}q \ \textrm{e}^{\textrm{i}{2} q x}\sum _{k \in {{\mathbb {Z}}}} \left( Z^{\textrm{II}}_{\textrm{tot}}\left( q, t, \sigma + k \right) +Z^{\textrm{II}}_{\textrm{tot}}\left( - q - {\textrm{i}\over 2} , t, \sigma + k + {1\over 2} \right) \right) \\&= { 2^{11/12} \pi ^{3/2} t^{3/16} \over \textrm{e}^{3\zeta '(-1)}\textrm{e}^{4 \sqrt{t}} } \textrm{e}^{ -4 t^{1/4} \cosh x+ {x\over 2}} \sum _{N\geqslant 0} \Psi _N(\textrm{e}^{{x}}, t) \left( {\cos (2\pi \sigma )\over 2\pi }\right) ^N \end{aligned} } \end{aligned}$$

Following Sect. 3.2, we get the square integrable eigenfunctions of (3.5) when we evaluate (6.7) at the values of $\sigma$ which satisfy the quantization condition (3.8). That is,

6.8 $$\begin{aligned} \boxed { \begin{aligned} \varphi _n(x,t)=&{ \textrm{e}^{3\zeta '(-1)}\textrm{e}^{4 \sqrt{t}} \over 2^{11/12} \pi ^{3/2} t^{3/16}}\\&\int \limits _{{{\mathbb {R}}}} \textrm{d}q \, \textrm{e}^{\textrm{i}{2} q x}\sum _{k \in {{\mathbb {Z}}}} \left( Z^\textrm{II}_{\textrm{tot}}\left( q, t, k + {1\over 2} + \textrm{i}\sigma _n \right) + Z^{\textrm{II}}_{\textrm{tot}}\left( - q - {\textrm{i}\over 2} , t, k + \textrm{i}\sigma _n \right) \right) \end{aligned}}\nonumber \\ \end{aligned}$$

where $\sigma _n$ are solutions of (3.8). In Fig. 1, we plot the right-hand side of (6.8) for the two smallest values of $\sigma _n$ that satisfy the quantization condition (3.8). As a cross-check, we also verified this result by a purely numerical analysis of the operator (3.5), see Sect. 6.4.

Let us make a few comments on the analytic properties of the gauge theoretic functions.

- The function $Z^{\textrm{Nek}}( t, \sigma )$ has poles when $2\sigma \in {{\mathbb {Z}}}$ and $Z^{\textrm{II}}_{\textrm{tot}}(q, t, \sigma )$ has additional poles when *q* and $\sigma$ satisfy $q = \frac{\textrm{i}}{2} \pm \textrm{i}\sigma + \textrm{i}\ell$ with $\ell \in {\mathbb {Z}}$.
- If we are strictly interested only in the spectral problem associated with the integral kernel (3.5), then $q \in {{\mathbb {R}}}$ and $\sigma \in \frac{1}{2} + {\textrm{i}} {{\mathbb {R}}}_{>0}$. So these poles are not realized.
- However, we can go beyond this special domain. For example, if we consider the Zak transform of $Z^{\textrm{Nek}}(t, \sigma )$ appearing on the left-hand side of (3.9), then this has no longer poles in $\sigma$: the summation over *k* in (3.9) removes the poles. Likewise, it seems that the summation over integers and the particular combination of defect partition functions appearing in the integrand on the left-hand side of (6.7) has also the effect of removing the poles.

In the forthcoming subsections, we test (6.5) and (6.8) in several ways.

### Testing $N=0$

As a first check of (6.5), we test the $N=0$ case. From (3.18), one can see that $\Psi _{0}(\textrm{e}^{{x}}, t) = 1$ so that the right-hand side of (6.5) is

6.9 $$\begin{aligned}{} & {} \left( \textrm{i}\frac{2^{11/12}\sqrt{\pi } t^{3/16}}{\textrm{e}^{3\zeta '(-1)}\textrm{e}^{4 \sqrt{t}}} \right) \int \limits _{{\mathbb {R}}} {\textrm{d}x} \ \textrm{e}^{-\textrm{i}{2} q x} \textrm{e}^{-4 t^{1/4} \cosh x + {x\over 2}} \nonumber \\{} & {} \qquad = \left( \textrm{i}\frac{2^{11/12}\sqrt{\pi } t^{1/16}}{\textrm{e}^{3\zeta '(-1)}\textrm{e}^{4 \sqrt{t}}} \right) 2 t^{1/8} K_{\textrm{i} 2 q - \frac{1}{2}}\left( 4 t^{1/4}\right) \, , \end{aligned}$$

where *K* is the modified Bessel function of the second kind. By expanding at small *t*, we find that the Bessel function has the following structure,

6.10 $$\begin{aligned} 2 t^{1/8} K_{ \textrm{i} 2 q-\frac{1}{2}}\left( 4 t^{1/4}\right) = F(q,t) + F\left( -q-{\textrm{i}\over 2},t\right) \, , \end{aligned}$$

for some function *F*(*q*, *t*). For instance, we have when $\textrm{i}2 q - \frac{1}{2} \notin {{\mathbb {Z}}}$

6.11 $$\begin{aligned} F(q,t)= \textrm{e}^{\textrm{i}\frac{q}{2} \log (t)} \left[ 2^{ \textrm{i} 2 q - \frac{1}{2}} \Gamma \left( - \textrm{i} 2 q + \frac{1}{2}\right) -2^{ \textrm{i} 2 q + \frac{3}{2}} \Gamma \left( - \textrm{i} 2 q -\frac{1}{2}\right) \sqrt{t} +{\mathcal {O}}\left( t\right) \right] \, ,\nonumber \\ \end{aligned}$$

Hence, we already see the structure of the left-hand side of (6.5) appearing. On the gauge theory side, we can perform the integral at small *t* by using Cauchy’s residue theorem,

6.12 $$\begin{aligned}{} & {} \int \limits _{ {{\mathbb {R}}}+\textrm{i}\sigma _*}{\textrm{d}\sigma } \tan \left( 2 \pi \sigma \right) Z_\textrm{tot}^{\textrm{II}}(q, t, \sigma ) \nonumber \\{} & {} \quad = \int \limits _{ {{\mathbb {R}}}+\textrm{i}\sigma _*}{\textrm{d}\sigma } \tan (2 \pi \sigma ) \textrm{e}^{\textrm{i}\frac{q}{2} \log (t)} t^{\sigma ^2}\frac{\Gamma \left( - \textrm{i}q-\sigma +\frac{1}{2}\right) \Gamma \left( - \textrm{i}q+\sigma +\frac{1}{2}\right) }{G(1-2 \sigma ) G( 1 + 2 \sigma )}\left( 1+{\mathcal {O}}(t)\right) \nonumber \\{} & {} \quad = \left( \textrm{i}\frac{2^{11/12} \sqrt{\pi } t^{1/16}}{\textrm{e}^{3 \zeta '(-1)} \textrm{e}^{4 \sqrt{t}}} \right) \textrm{e}^{\textrm{i}\frac{q}{2} \log (t)} \left[ 2^{ \textrm{i} 2 q - \frac{1}{2}} \Gamma \left( - \textrm{i} 2 q + \frac{1}{2}\right) -2^{ \textrm{i} 2 q + \frac{3}{2}} \Gamma \left( - \textrm{i} 2 q-\frac{1}{2}\right) \sqrt{t} \right. \nonumber \\{} & {} \qquad \left. + 2^{ \textrm{i} 2 q + \frac{5}{2}} \Gamma \left( - \textrm{i} 2 q - \frac{3}{2}\right) \, t + {\mathcal {O}}\left( t^{3/2}\right) \right] . \end{aligned}$$

To get the last line in (6.12), we have included the first instanton correction in $Z_\textrm{tot}^{\textrm{II}}$ (6.4), and higher instanton corrections can be treated similarly. The poles contributing to the integral in (6.12) are

6.13 $$\begin{aligned} \sigma = { \ell \over 2} \quad \text {and} \quad \sigma ={1\over 4} + { \ell \over 2} \quad \text {with} \quad \ell \in {{\mathbb {Z}}}~. \end{aligned}$$

By employing the series expansions (6.11) and (6.12), we can systematically verify (6.5) for $N=0$, order by order in *t*.

### Testing $N=1$

**Table 1:** Comparison between the two sides of (6.5) for $N=1$, $t= 1 / 55 \pi ^4$ with $q= 1/9 + \textrm{i} 2 / \sqrt{3}$ (upper left), $q= 1/ \pi$ (upper right), and $q= \textrm{i} / 3$ (lower). $I_1$ is the integral (6.15) appearing on the right-hand side of (6.5); $n^{\text {inst}}$ refers to the number of instantons we include in the defect partition function appearing on the left-hand side of (6.5)

$$n^{\text {inst}}$$	
0	2.8372834788 + 4.7204648771 i
1	2.8312289304 + 4.7137559136 i
2	2.8313227416 + 4.7137434937 i
3	2.8313226948 + 4.7137435320 i
$I_1$	2.8313226948 + 4.7137435320 i
0	0.050235280369 + 0.018141366757 i
1	0.050242014312 + 0.018111611547 i
2	0.050241915710 + 0.018111648299 i
3	0.050241915600 + 0.018111648316 i
$I_1$	0.050241915600 + 0.018111648316 i
0	0.1587865901507390 i
1	0.1587680111233355 i
2	0.1587680126408577 i
3	0.1587680126408951 i
$I_1$	0.1587680126408951 i

As a second consistency check of (6.5), we test the $N=1$ case. First, we note that by a change of variables, we can rewrite the double integral appearing on the right-hand side of (6.5) as a one-dimensional integral. Let us define

6.14 $$\begin{aligned} \begin{aligned}&I_1(q,t) = \left( \textrm{i}{2^{11/12} \sqrt{\pi } {t}^{3/16}\over \textrm{e}^{3 \zeta '(-1)} \textrm{e}^{4 \sqrt{{t}}} \left( 4 \pi \right) } \right) \int \limits _{{\mathbb {R}}} {\textrm{d}x} \ \textrm{e}^{-\textrm{i}{2} q x} \textrm{e}^{- 4 t^{1/4} \cosh x + \frac{x}{2}} \Psi _1(\textrm{e}^{{x}}, t). \end{aligned}\nonumber \\ \end{aligned}$$

After some algebra, we get

6.15 $$\begin{aligned}{} & {} I_1(q,t) = \left( \textrm{i}\frac{ 2^{11/12} t^{3/16}}{\sqrt{\pi } \textrm{e}^{3 \zeta '(-1)} \textrm{e}^{4 \sqrt{t}}} \right) \nonumber \\{} & {} \quad \times \int \limits _{3}^{+\infty } \textrm{d}U \frac{ \left( \left( \frac{U^2+\sqrt{U^4-10 U^2+9}-3}{2 U}\right) ^{- \textrm{i} 2 q + \frac{1}{2}}- \left( \frac{U^2+\sqrt{U^4-10 U^2+9}-3}{2 U}\right) ^{ \textrm{i} 2 q - \frac{1}{2}}\right) K_{\textrm{i} 2 q - \frac{1}{2}}\left( 4 \root 4 \of {t} U\right) }{U-U^{-1}}.\nonumber \\ \end{aligned}$$

One useful observation is that the above integral vanishes when $q = -\textrm{i}/ 4$, which is in perfect agreement with the left-hand side of (6.5). Unfortunately, we cannot compute the integral (6.15) analytically. Hence, for $N=1$, the test of (6.5) is done numerically and we find perfect agreement. One such test is given in Table 1.

### Testing Large *N* with a ’t Hooft Limit

Another analytical test of the identity (6.5) consists of comparing both sides in the ’t Hooft limit, whereas in (4.2)

6.16 $$\begin{aligned} N \rightarrow + \infty \, , \quad \epsilon \rightarrow 0 \, , \quad \lambda = N \epsilon > 0 \, , \end{aligned}$$

and with the ’t Hooft coupling $\lambda$ fixed. We will need to use that *q* and *t* scale as in (2.4),

6.17 $$\begin{aligned} q = \frac{y}{2 \epsilon } \, , \quad t = \left( \frac{\Lambda }{\epsilon }\right) ^4 \, , \end{aligned}$$

with both the position of the defect $y \in {\mathbb {C}}$ and the instanton counting parameter $\Lambda > 0$ kept fixed.

The computation of the ’t Hooft limit of (6.5) is simplified by using the corresponding statement for the theory without defects, which is given in (3.15) and was obtained in [48, 49]. In particular, one can divide both sides of (6.5) by (3.15) to get

6.18 $$\begin{aligned}{} & {} \frac{\int \limits _{{\mathbb {R}}+\textrm{i}\sigma _*} \textrm{d}\sigma \frac{\tan \left( 2 \pi \sigma \right) }{\left[ 2 \cos \left( 2 \pi \sigma \right) \right] ^N} Z^\text {Nek}\left( \left( \frac{\Lambda }{\epsilon }\right) ^4, \sigma \right) \left[ Z^\textrm{II}\left( \frac{y}{2 \epsilon }, \left( \frac{\Lambda }{\epsilon }\right) ^4, \sigma \right) + \left( -1\right) ^N Z^\textrm{II}\left( \frac{- y - \textrm{i} \epsilon }{2 \epsilon }, \left( \frac{\Lambda }{\epsilon }\right) ^4, \sigma \right) \right] }{ \left( 2 \pi \right) \int \limits _{{\mathbb {R}}+\textrm{i}\sigma _*} \textrm{d}\sigma \frac{\tan \left( 2 \pi \sigma \right) }{\left[ 2 \cos \left( 2 \pi \sigma \right) \right] ^N} Z^\text {Nek}\left( \left( \frac{\Lambda }{\epsilon }\right) ^4, \sigma \right) } \nonumber \\{} & {} \quad = \frac{\sqrt{ \Lambda }}{\sqrt{ \pi \epsilon }} \int \limits _{\mathbb {R}} \textrm{d}x \, \exp \left( - \frac{\textrm{i}}{\epsilon } x y \right) \frac{\exp \left( - 4 \left( \frac{\Lambda }{\epsilon }\right) \cosh \left( x\right) + \frac{x}{2}\right) \Psi _N \left( \textrm{e}^x , \left( \frac{\Lambda }{\epsilon }\right) ^4 \right) }{Z\left( \left( \frac{\Lambda }{\epsilon }\right) ^4, N \right) } \, . \end{aligned}$$

Note that (6.18) is by (3.15) equivalent to (6.5), but rewritten in a form suitable and convenient for the ’t Hooft limit (6.16).

#### The ’t Hooft Limit on the Gauge Theory Side

The general pattern of the ’t Hooft expansion of the left-hand side in (6.18) is the same as in Sect. 5.2. Using that the integration variable $\sigma$ can be related to the Coulomb branch parameter *a* by (3.11),

6.19 $$\begin{aligned} \sigma = \textrm{i} \frac{a}{2 \epsilon } \, , \end{aligned}$$

one expands the logarithm of the Nekrasov partition function $Z^\textrm{Nek}$ in even powers of $\epsilon$ with the leading order being $\epsilon ^{-2}$. On the other hand, the expansion of the logarithm of the defect partition function $Z^\textrm{II}$ contains all integer powers of $\epsilon$ starting from $\epsilon ^{-1}$,

6.20 $$\begin{aligned} (2\pi )^{-1}Z^{\textrm{II}}\left( \frac{y}{2 \epsilon }, \left( \frac{\Lambda }{\epsilon }\right) ^4, \textrm{i} \frac{a}{2 \epsilon } \right) \simeq \exp \left( \sum _{n\geqslant 0} {\mathcal {W}}^\textrm{II}_n(y) \epsilon ^{n-1} \right) \, . \end{aligned}$$

Hence, the saddles of both integrals on the left-hand side of (6.18) are determined by the same equation (5.15). This gives the functional relation $a \left( \Lambda , \lambda \right)$, but for us it will be convenient to rather invert this to $\lambda \left( \Lambda , a \right)$ and keep *a* explicitly. Keeping this in mind, the ’t Hooft limit of the left-hand side of (6.18) leads eventually to

6.21 $$\begin{aligned}{} & {} \exp \left\{ \frac{1}{\epsilon } {\mathcal {W}}_0^{\textrm{II}}\left( y\right) + \left[ - \frac{\left( \partial _a{\mathcal W}_0^{\textrm{II}}\left( y\right) \right) ^2}{2 \partial _a^2 {\mathcal {F}}_0} + {\mathcal {W}}_1^{\textrm{II}}\left( y\right) \right] + {\mathcal {O}}\left( \epsilon \right) \right\} \nonumber \\{} & {} \quad + \exp \left\{ \frac{1}{\epsilon } \left[ \textrm{i}\pi \lambda + {\mathcal {W}}_0^{\textrm{II}} \left( -y\right) \right] + \left[ \textrm{i}\partial _y {\mathcal {W}}_0^{\textrm{II}}\left( -y\right) - \frac{\left( \partial _a{\mathcal W}_0^{\textrm{II}}\left( -y\right) \right) ^2}{2 \partial _a^2 {\mathcal {F}}_0} + {\mathcal {W}}_1^{\textrm{II}}\left( -y\right) \right] + {\mathcal {O}}\left( \epsilon \right) \right\} , \nonumber \\ \end{aligned}$$

where we suppressed the functional dependence on $\Lambda$ and *a* in the notation.

#### The ’t Hooft Limit on the Matrix Model Side

Consider the Fourier transform on the right-hand side in (6.18),

6.22 $$\begin{aligned} \frac{\sqrt{2 \Lambda }}{\sqrt{2 \pi \epsilon }} \int \limits _{\mathbb {R}} \textrm{d}x \exp \left( - \frac{\textrm{i}}{\epsilon } x y \right) \frac{\exp \left( - 4 \left( \frac{\Lambda }{\epsilon }\right) \cosh \left( x\right) + \frac{x}{2}\right) \Psi _N \left( \textrm{e}^x, \left( \frac{\Lambda }{\epsilon }\right) ^4 \right) }{Z\left( t , N \right) }, \qquad \end{aligned}$$

where the ’t Hooft expansion of the integrand is

6.23 $$\begin{aligned}{} & {} \frac{\exp \left( - 4 \left( \frac{\Lambda }{\epsilon }\right) \cosh \left( x\right) + \frac{x}{2}\right) \Psi _N \left( \textrm{e}^x, \left( \frac{\Lambda }{\epsilon }\right) ^4 \right) }{Z\left( t , N \right) }\nonumber \\{} & {} \quad = \exp \left[ \frac{1}{\epsilon }{\mathcal {T}}_0\left( \textrm{e}^x\right) + \frac{x}{2} + {\mathcal {T}}_1\left( \textrm{e}^x\right) + {\mathcal {O}}\left( \epsilon \right) \right] \, , \end{aligned}$$

by using (4.9) and again suppressing the functional dependence on the other variables. In the limit $\epsilon \rightarrow 0$, the Fourier transform in (6.22) is dominated by its saddles and becomes

6.24 $$\begin{aligned}{} & {} \sum _{s} \exp \left\{ \frac{1}{\epsilon } \widehat{{\mathcal {T}}_{s, 0}} \left( y\right) + \widehat{{\mathcal {T}}_{s, 1}} \left( y\right) + {\mathcal {O}}\left( \epsilon \right) \right\} \nonumber \\{} & {} \quad = \sum _{s} \exp \left\{ \frac{1}{\epsilon } \left[ - \textrm{i} x_s y + {\mathcal {T}}_0 \left( \textrm{e}^{x_s} \right) \right] \right. \nonumber \\{} & {} \qquad \left. + \left[ \frac{\log \left( 2 \Lambda \right) }{2} - \frac{\log \left( - \partial _x^2 {\mathcal {T}}_0 \left( \textrm{e}^{x_s}\right) \right) }{2} + \frac{x_s}{2} + {\mathcal {T}}_1 \left( \textrm{e}^{x_s}\right) \right] + {\mathcal {O}}\left( \epsilon \right) \right\} . \qquad \end{aligned}$$

The sum over *s* is a sum over the saddles and $x_s = x_s\left( y\right)$ is determined by the saddle point equation,

6.25 $$\begin{aligned} y + \textrm{i} \partial _x {\mathcal {T}}_0 \left( \textrm{e}^x \right) = y - \textrm{i} 2\Lambda \frac{\sqrt{ \textrm{e}^{2x} -\texttt {g}^{2}}\sqrt{\textrm{e}^{2x}-\texttt {g}^{-2}}}{\textrm{e}^{x}} = 0 \, , \end{aligned}$$

where we used (4.34) and $z = \exp \left( x \right)$. Taking the square of this equation gives the Seiberg–Witten curve (4.35) if we take as before (4.37),

6.26 $$\begin{aligned} \texttt {g}^2+\texttt {g}^{-2}={u\over 4 \Lambda ^2} \, , \quad \texttt {g}^{\pm 2} = \left( \frac{u}{8 \Lambda ^2}\right) \mp \sqrt{\left( \frac{u}{8 \Lambda ^2}\right) ^2 - 1} \, . \end{aligned}$$

This leads to the following two solutions,

6.27 $$\begin{aligned} \textrm{e}^{2x_\pm \left( y \right) } = z_\pm ^2 \left( y\right) = {-\left[ \frac{\left( y^2 - u \right) \pm \sqrt{\left( y^2 - u \right) ^2 - 64 \Lambda ^4}}{8 \Lambda ^2}\right] }. \end{aligned}$$

Let us take a moment to consider the behavior of $z_\pm \left( y\right)$ as a function of *y*. One can check that $z_\pm \left( y\right)$ is real and outside the branch cut region of the matrix model if and only if $\textrm{i} y \in {\mathbb {R}} \setminus \left\{ 0 \right\}$. It is important to note that with this choice of $\textrm{i} y \in {\mathbb {R}} \setminus \left\{ 0 \right\}$ one has $z_-^2\left( y \right) > 1 / \texttt {g}^2$ and $0 \leqslant z_+^2\left( y \right) < \texttt {g}^2$. Moreover, there are no possible choices of $y \in {\mathbb {C}}$ such that $0 \leqslant z_-^2\left( y \right) < \texttt {g}^2$ or $z_+^2\left( y \right) > 1 / \texttt {g}^2$. One finds on the other hand that $z_\pm \left( y\right)$ is real and inside the branch cut region if and only if $0 \leqslant y^2 \leqslant u - 8 \Lambda ^2$, and also that $z_\pm \left( y\right)$ is purely imaginary if and only if $y^2 \geqslant u + 8 \Lambda ^2$. For all other choices of $y \in {\mathbb {C}}$, one will find generic complex $z_\pm \left( y\right)$.

#### Comparing the Gauge Theory and the Matrix Model

To analyze the leading order of the ’t Hooft expansion in (6.24) with the saddles (6.27), it is convenient to separately look at the case $y = 0$ and the derivative with respect to *y*. The reason is that the latter simplifies considerably as a consequence of the saddle point Eq. (6.25). Setting $y = 0$ serves then as a check of the constant term.

Let us first look at the *y* derivative of the leading-order part. At the matrix model side (6.24), one gets by making use of the saddle point Eq. (6.25) and its solutions

6.28 $$\begin{aligned} \frac{\textrm{d}}{\textrm{d}y} \widehat{{\mathcal {T}}_{\pm , 0}}\left( y\right) = - \textrm{i} x_\pm \left( y \right) \, . \end{aligned}$$

Comparing this to the leading order of the gauge theory (6.20), we can check that^25^

6.29 $$\begin{aligned} \begin{aligned} \frac{\textrm{d}}{\textrm{d}y} \left[ {\mathcal {W}}_0^{\textrm{II}}\left( \pm y\right) - \widehat{{\mathcal {T}}_{\pm S, 0}}\left( y\right) \right] =0 \, , \end{aligned} \end{aligned}$$

where $S = \textrm{sgn}\left[ \textrm{arg}\left( \textrm{i} \left( y^2 - a^2 \right) \right) \right]$ with the convention that $\textrm{sgn}\left( 0 \right) = -1 \,$.

Let us then look at the constant term for $y = 0$. At the gauge theory side, we have for the leading order (6.20)

6.30 $$\begin{aligned} {\mathcal {W}}_0^{\textrm{II}}\left( 0 \right) = - \frac{\pi }{2} \left| a\right| . \end{aligned}$$

From (6.27), one can see that $z_\pm \left( 0 \right) = \texttt {g}^{\pm } > 0$, with $\texttt {g}^{\pm }$ as in (6.26). Using (4.10) gives for the leading order of the matrix model (6.24)

6.31 $$\begin{aligned} \widehat{{\mathcal {T}}_{\pm , 0}} \left( 0 \right) = {\mathcal {T}}_0 \left( z_\pm \left( 0\right) \right) = - 2 \Lambda \left( \texttt {g}+ \texttt {g}^{-1} \right) + 2 \int \limits _{+ \infty }^{\texttt {g}^{\pm 1}} \textrm{d} z \, \lambda \omega _+^0\left( z\right) \, , \end{aligned}$$

where the even planar resolvent $\omega _+^0\left( z\right)$ is given in (4.30). Note that the difference between the leading terms of the two saddles is

6.32 $$\begin{aligned} \left[ \widehat{{\mathcal {T}}_{+, 0}} \left( 0\right) - \widehat{{\mathcal {T}}_{-, 0}} \left( 0\right) \right] = - 2 \int \limits _{\texttt {g}}^{\texttt {g}^{-1}} \textrm{d} z \, \lambda \omega _+^0\left( z\right) = \textrm{i} \pi \lambda \, . \end{aligned}$$

The last equality can be obtained in an $\Lambda \rightarrow 0$ expansion or exactly using [129, eq. 4.18], which shows that this relation does not depend on the particular form of the potential. We have furthermore that

6.33 $$\begin{aligned}{} & {} \widehat{{\mathcal {T}}_{-, 0}} \left( 0 \right) = - 2 \Lambda \left( \texttt {g}+ \texttt {g}^{-1} \right) + 2 \int \limits _{+ \infty }^{\texttt {g}^{- 1}} \textrm{d} z \ \lambda \omega _+^0\left( z\right) \nonumber \\{} & {} \quad = - 2 \Lambda \left[ \frac{2 \textrm{E}\left( \texttt {g}^4 \right) + \left( \texttt {g}^{-2} - \texttt {g}^2 \right) \left[ - 2 \texttt {g}^2 \textrm{K}\left( \texttt {g}^4 \right) + \textrm{K} \left( \texttt {g}^{-4} \right) + \textrm{i} \textrm{K}\left( 1 -\texttt {g}^{-4} \right) \right] }{\texttt {g}}\right] \nonumber \\{} & {} \quad = - \frac{\pi }{2} \left| a \right| \, , \end{aligned}$$

with $\textrm{K}$ and $\textrm{E}$ the complete elliptic integrals of the first (C.1) and second (C.2) kind, respectively. The last equality was found in an $\Lambda \rightarrow 0$ or equivalently $\texttt {g}\rightarrow 0$ expansion using (6.26) and the Matone relation (5.17). Hence, from (6.30), (6.32), and (6.33)

6.34 $$\begin{aligned} \begin{aligned} {\mathcal {W}}_0^{\textrm{II}}\left( 0\right) - \widehat{{\mathcal {T}}_{-, 0}}\left( 0\right)&= 0 \, , \\ \textrm{i} \pi \lambda + {\mathcal {W}}_0^{\textrm{II}}\left( 0 \right) - \widehat{{\mathcal {T}}_{+, 0}}\left( 0 \right)&= 0 \, . \end{aligned} \end{aligned}$$

So the constant parts of the leading-order terms agree, and together with (6.29) this proves the equality in (6.18) and hence (6.5) to leading order in the ’t Hooft limit (6.16).

The subleading order can be checked in analogy with Sect. 5. Matching at higher order in $\epsilon$ can then be inferred from topological recursion, as we discussed near the end of Sect. 5.

### Numerical Eigenfunctions

The numerical analysis of the spectrum and the eigenfunctions for the integral kernel $\rho$ (3.5) is done exactly as in [139, Sect. 2.2]. To make the presentation self-contained, let us review the strategy of [139, Sect. 2.2]. We are interested in studying numerically the eigenvalue equation

6.35 $$\begin{aligned} \int \limits _{{{\mathbb {R}}}}\textrm{d}y \, \rho (x, y)\varphi _n(y,t)=E_n \varphi _n(x,t) \, , \end{aligned}$$

where the kernel $\rho (x,y)$ is defined in (3.5). It is convenient to decompose $\rho (x,y)$ as

6.36 $$\begin{aligned} \rho (x,y)=\sum _{k \geqslant 0}\rho _k(x)\rho _k(y) \, , \quad \rho _k(x)=\frac{\tanh ^k\left( \frac{x}{2}\right) \exp \left( -4 t^{1/4} \cosh (x)\right) }{\cosh \left( \frac{x}{2}\right) }, \nonumber \\ \end{aligned}$$

and to define

6.37 $$\begin{aligned} v_k^{(n)}(t)=\int \limits _{{{\mathbb {R}}}}\textrm{d}y \, \rho _k(y) \varphi _n(y,t) \, . \end{aligned}$$

Then, (6.35) reads

6.38 $$\begin{aligned} \sum _{k\geqslant 0} \rho _k(x) v_k^{(n)}(t) =E_n \varphi _n(x,t) \, , \end{aligned}$$

which we can also write as

6.39 $$\begin{aligned} \sum _{k\geqslant 0}H_{\ell , k}v_k^{(n)}(t)=E_n v_{\ell }^{(n)}(t)~, \end{aligned}$$

with *H* the infinite-dimensional Hankel matrix defined by

6.40 $$\begin{aligned} H_{k, \ell }=\int \limits _{{\mathbb {R}}} \textrm{d}x \frac{\tanh ^{k + \ell }\left( \frac{x}{2}\right) \exp \left( -8 {t^{1/4}} \cosh (x)\right) }{\cosh ^2\left( \frac{x}{2}\right) } \, , \quad k , \ell \geqslant 0 \, . \end{aligned}$$

This means that the eigenvalues of *H* coincide with those of $\rho \left( x, y \right)$ and the eigenvectors of *H* give the eigenfunctions of $\rho \left( x, y \right)$ via (6.38). The advantage of working with *H* is that we can numerically compute its eigenvalues and eigenfunctions by truncating the matrix to a finite size while maintaining control over the numerical error due to the truncation. Let $v^{(n,M)}(t)$ be the $n^{\textrm{th}}$ eigenvector of the Hankel matrix *H* (6.40) truncated at size *M*. Defining $\varphi _n^{(M)}(x,t)$ by

6.41 $$\begin{aligned} \varphi _n^{(M)}(x,t) = \sum _{k = 0}^M \rho _k(x) v^{(n,M)}_k \, , \quad v^{(n,M)}=\left( \begin{array}{c} v^{(n,M)}_0 \\ \vdots \\ v^{(n,M)}_M \end{array}\right) \, , \end{aligned}$$

we recover the true eigenfunctions of the kernel $\rho$ in the $M \rightarrow + \infty$ limit,

6.42 $$\begin{aligned} \lim _{M \rightarrow + \infty }\varphi _n^{(M)}(x,t)\propto \varphi _n(x,t) \, , \end{aligned}$$

where the proportionality factor is a numerical constant and $\varphi _n$ is the $n^{\textrm{th}}$ eigenfunction of (6.35) in the normalization of (6.8). We computed the left-hand side of (6.42) numerically and checked that this numerical expression agrees with the eigenfunctions computed by using the defect expression on the right-hand side of (6.8) with high precision. For instance, for $t=1/(100 \pi ^8)$, by including 0 instantons in (6.8) we get a pointwise agreement of the order $10^{-6}$. Likewise, by including 1, 2 and 3 instantons we get a pointwise agreement of the order $10^{-11}$, $10^{-16}$ and $10^{-22}$, respectively^26^.

## Outlook

In this paper, we have shown that the eigenfunctions of the operator (1.1) are computed by surface defects in ${\mathcal {N}}=2$, ${{\,\mathrm{SU(2)}\,}}$ SYM in the self-dual phase of the four-dimensional $\Omega$-background ($\epsilon _1+\epsilon _2=0$). This result, together with [48, 49, 51], extends the correspondence between 4d ${\mathcal {N}}=2$ theories and spectral theory to a new class of operators. In addition, we have expressed the eigenfunctions of these operators in closed form via a matrix model average (2.2). This provides a representation for the surface defect partition function which resums both the instanton and the $\epsilon$ expansions. In this way, we have a manifest interpolation from the weak to the strong coupling region. In particular, the strong coupling expansion in $1/\Lambda$ (exact in $\epsilon$ and $a_D$) corresponds to the expansion of the matrix model around its Gaussian point, and hence, it is obtained straightforwardly.

Some further comments and generalizations:

- In this work, we focused on the specific example of 4d, ${\mathcal {N}}=2$, ${{\,\mathrm{SU(2)}\,}}$ SYM and the operator (1.1). It would be interesting to extend our results in a systematic way to all 4d ${\mathcal {N}}=2$ theories. For example, in the case of ${\mathcal {N}}=2$, ${{\,\mathrm{SU(N)}\,}}$ SYM we have $N-1$ non-commuting Fermi gas operators as discussed in [49, 51]. We expect their eigenfunctions to be computed by surface defects in ${{\,\mathrm{SU(N)}\,}}$ SYM in the self-dual phase of the $\Omega$ background.
- Our results should follow from the *open* version of the TS/ST correspondence [63, 83]. We will report on this somewhere else [140].
- The Fredholm determinant of (1.1) computes the tau function of the Painlevé $\mathrm III_3$ equation at specific initial conditions. It would be interesting to understand the role of the eigenfunctions of (1.1) in the context of Painlevé equations. In particular, the relation to the solution of the linear system associated with Painlevé Eqs. [18, 59] as well as with [141].
- The Fredholm determinant and the spectral traces of (1.1) can also be expressed via a pair of coupled TBA equations closely related to *two-dimensional* theories [46, 142]. It would be interesting to understand this better since this may reveal an interesting 4d-2d interplay characterizing directly the self-dual phase of the $\Omega$-background.
- The operator (1.1) is a particular example of a Painlevé kernel whose Fredholm determinant computes the tau function. A more general class of Fredholm determinants was constructed in [143–146]. It would be interesting to see if also in this case the corresponding (formal) eigenfunctions are related to surface defects.
- It is well known that the canonical quantization of the SW curve for ${{\,\mathrm{SU(2)}\,}}$ SYM leads to the Mathieu operator (3.1). We expect a different quantization scheme to produce the operator (1.1). It is important to understand what this other quantization scheme is. Since the spectral analysis of (1.1) is encoded in the self-dual phase of the $\Omega$-background, a natural quantization scheme to investigate would be the one arising in the context of the topological recursion [120–123].

## The Refined Open Topological Vertex and the Type II Defect

In this appendix, we explain how the type II defect partition function of 4d, ${\mathcal {N}} = 2$, $\textrm{SU}\left( 2\right)$ super Yang–Mills can be obtained from open topological strings on the toric Calabi–Yau manifold known as local ${\mathbb {F}}_0 = {\mathbb {P}}^1 \times {\mathbb {P}}^1$.

### Open Topological Strings on Local ${\mathbb {F}}_0$

To find the open string partition function on local ${\mathbb {F}}_0$, we use [147] and [148]. We will closely follow the notation and presentation of [148].

#### Notation and Conventions

The variables $Q_{B,F}$ are related to the Kähler parameters $T_{B,F}$ of the base and fiber ${\mathbb {P}}^1$’s, respectively,

A.1 $$\begin{aligned} Q_{B,F} =\exp \left( - T_{B,F} \right) \, , \quad T_{B,F} = - \log \left( Q_{B,F} \right) \, , \end{aligned}$$

and $Q_1$ is similarly the open Kähler parameter of the brane [148, pp. 9-14, 50]. The variables *t*, *q* are similarly connected to the $\Omega$-background parameters $\epsilon _{1,2}$ by

A.2 $$\begin{aligned} t =\exp \left( \textrm{i} \epsilon _1 \right) \, , \quad q =\exp \left( - \textrm{i} \epsilon _2 \right) \, . \end{aligned}$$

It is important to note that these variables are not related to the *t* and *q* appearing in the main text.

#### The Refined Open Topological Vertex

Important building blocks of the partition function are the so-called Nekrasov factors

A.3 $$\begin{aligned} N_{\mu , \nu } \left( Q; t, q \right)= & {} \prod _{k, \ell = 1}^{+ \infty } \frac{1 - Q \ q^{\nu _k - \ell } t^{\mu _\ell ^t-k+1}}{1 - Q \ q^{-\ell } t^{-k+1}} \nonumber \\= & {} \prod _{\left( k, \ell \right) \in \nu } \left( 1 - Q \ q^{\nu _k - \ell } t^{\mu _\ell ^t-k+1} \right) \prod _{\left( k, \ell \right) \in \mu } \left( 1 - Q \ q^{-\mu _k + \ell - 1} t^{-\nu _\ell ^t+k} \right) ,\nonumber \\ \end{aligned}$$

where $\mu$ and $\nu$ are integer partitions:

A.4 $$\begin{aligned} \mu = \left\{ \mu _1, \mu _2, \mu _3, \cdots \ | \ \forall \ k, \ell \in {\mathbb {N}} \setminus \left\{ 0 \right\} : \left( \mu _k \in {\mathbb {N}} \right) \wedge \left( k \leqslant \ell \Rightarrow \mu _k \geqslant \mu _\ell \right) \right\} . \nonumber \\ \end{aligned}$$

Hence, $\left( k, \ell \right) \in \mu$ is any pair $k, \ell \in {\mathbb {N}}\setminus \left\{ 0 \right\}$ such that $1 \leqslant \ell \leqslant \mu _k$. Integer partitions can be represented by a Young diagram, and the transposed partition $\mu ^t$ is obtained by transposing the Young diagram,

A.5 $$\begin{aligned} \mu ^t =\left\{ \mu _1^t, \mu _2^t, \mu _3^t, \cdots \ | \ \forall \ \ell \in {\mathbb {N}} \setminus \left\{ 0 \right\} : \mu _\ell ^t =\left| \left\{ k \in {\mathbb {N}} \setminus \left\{ 0 \right\} \ | \ \mu _k \geqslant \ell \right\} \right| \right\} . \nonumber \\ \end{aligned}$$

The key ingredient in the topological vertex computation is^27^,

A.6 $$\begin{aligned}{} & {} C_{\mu , \nu } \left( Q_1, Q_F, t, q \right) =\frac{N_{\emptyset , \mu ^t} \left( Q_1 \frac{t^2}{q}; t^{-1}, q^{-1} \right) N_{\emptyset , \nu } \left( Q_1 Q_F \frac{t^2}{q}; t^{-1}, q^{-1} \right) }{N_{\emptyset , \mu ^t} \left( Q_1 \frac{t}{q}; t^{-1}, q^{-1} \right) N_{\emptyset , \nu } \left( Q_1 Q_F \frac{t}{q}; t^{-1}, q^{-1} \right) } \nonumber \\{} & {} \quad \quad \frac{1}{N_{\mu ^t,\nu }\left( Q_F; t^{-1}, q^{-1} \right) N_{\mu ^t,\nu }\left( Q_F \frac{t}{q}; t^{-1}, q^{-1} \right) } \, , \end{aligned}$$

where $\emptyset$ is the empty partition $\{\}$. This gives for the “*open-closed t-brane partition function*” of local ${\mathbb {F}}_0$ [148, p. 50, eq. 5.4]

A.7 $$\begin{aligned}{} & {} Z^{\text {open-closed, t-brane}}_{{\mathbb {F}}_0} \left( Q_1 , Q_B, Q_F, t, q \right) \nonumber \\{} & {} \quad =\sum _{\mu , \nu } Q_B^{\left| \mu \right| + \left| \nu \right| } t^{||\nu ^t||^2} q^{||\mu ^t||^2} ||Z_\mu \left( t, q \right) ||^2 ||Z_\nu \left( q, t \right) ||^2 C_{\mu , \nu } \left( Q_1 , Q_F, t, q \right) \, ,\nonumber \\ \end{aligned}$$

where one defines

A.8 $$\begin{aligned}&\displaystyle |\mu | =\sum _{k=1}^{+\infty } \mu _k \, , \quad ||\mu ||^2 =\sum _{k=1}^{+\infty } \mu _k^2 \, , \end{aligned}$$

A.9 $$\begin{aligned}&\displaystyle \left| \left| Z_\mu \left( t, q \right) \right| \right| ^2 =Z_{\mu ^t} \left( t, q \right) Z_\mu \left( q, t \right) \, , \quad Z_\mu \left( t, q \right) =\prod _{\left( k , \ell \right) \in \mu } \left( 1 - q^{\mu _k - \ell } t^{\mu _\ell ^t - k + 1} \right) ^{-1} \, . \nonumber \\ \end{aligned}$$

The t-brane instanton partition function for local ${\mathbb {F}}_0$ is then [148, p. 50, eq. 5.7]

A.10 $$\begin{aligned} Z^{\text {t-brane, instanton}}_{{\mathbb {F}}_0} \left( Q_1 , Q_B, Q_F , t, q \right) =\frac{Z^{\text {open-closed, t-brane}}_{{\mathbb {F}}_0} \left( Q_1 , Q_B, Q_F, t, q \right) }{Z^{\text {closed}}_{{\mathbb {F}}_0} \left( Q_B, Q_F, t, q \right) } \, , \nonumber \\ \end{aligned}$$

where $Z^{\text {closed}}_{{\mathbb {F}}_0}$ is given by

A.11 $$\begin{aligned} Z^{\text {closed}}_{{\mathbb {F}}_0} \left( Q_B, Q_F, t, q \right) = Z^{\text {open-closed, t-brane}}_{{\mathbb {F}}_0} \left( 0 , Q_B, Q_F, t, q \right) \, . \end{aligned}$$

In addition, one has also the 1-loop part [148, pp. 50, 53, eqs. 5.6, A.15, A.22, A.25]

A.12 $$\begin{aligned} Z^\text {t-brane, 1-loop}_{{\mathbb {F}}_0}\left( Q_1 , Q_F, t, q \right)= & {} \left( \frac{Q_1 t}{q} ; \frac{1}{q} \right) _\infty \left( \frac{Q_1 Q_F t}{q} ; \frac{1}{q} \right) _\infty \nonumber \\= & {} \frac{1}{\left( Q_1 t ; q \right) _\infty \left( Q_1 Q_F t ; q \right) _\infty } \, , \end{aligned}$$

where $\left( a ; q \right) _\infty$ is the *q*-Pochhammer symbol. The complete partition function is then given by

A.13 $$\begin{aligned}{} & {} Z^{\text {t-brane}}_{{\mathbb {F}}_0} \left( Q_1 , Q_B, Q_F, t, q \right) \nonumber \\{} & {} \quad = Z^{\text {t-brane, 1-loop}}_{{\mathbb {F}}_0} \left( Q_1 , Q_F, t, q \right) Z^{\text {t-brane, instanton}}_{{\mathbb {F}}_0} \left( Q_1 , Q_B, Q_F, t, q \right) \, . \qquad \qquad \end{aligned}$$

Two particular phases of the refined topological vertex are of interest to us: the self-dual or Gopakumar–Vafa (GV) phase $\epsilon _1 + \epsilon _2 = 0$ or $t = q$, and the Nekrasov–Shatashvili (NS) phase $\epsilon _1 = 0$ or $t = 1$.

#### Non-Perturbative Topological Strings

We obtain the unrefined topological string amplitude if we set $g_s = - \epsilon _1 = \epsilon _2$. In addition, it was found in [52, 53, 149] that when one includes the NS phase of the refined topological vertex as well one obtains a non-perturbative completion of the topological string partition function on toric CYs. This was generalized to some extent from the closed to the open vertex in [63, 83], see also [17, 150]. In particular, for local ${{\mathbb {F}}}_0$ one finds [63, 83]

A.14 $$\begin{aligned} Z_{{\mathbb {F}}_0}^\text {open, np}\left( X, Q_B, Q_F, g_s \right){} & {} = Z_{{\mathbb {F}}_0}^\text {(p)}\left( X, g_s \right) Z_{{\mathbb {F}}_0}^\text {GV}\left( X, Q_B, Q_F, g_s \right) \nonumber \\{} & {} \quad Z_{{\mathbb {F}}_0}^\text {NS}\left( X^S, Q_B^S, Q_F^S, g_s \right) \, . \end{aligned}$$

We explain all functions and symbols involved below. The usual open topological string part $Z_{{\mathbb {F}}_0}^\text {GV}\left( X, Q_B, Q_F, g_s \right)$ is the self-dual phase of the open refined topological vertex (A.10), (A.12), (A.13),

A.15 $$\begin{aligned} Z_{{\mathbb {F}}_0}^\text {GV}\left( X, Q_B, Q_F, g_s \right) = Z^{\text {t-brane}}_{{\mathbb {F}}_0} \left( \frac{\sqrt{q}}{\sqrt{Q_F} X}, Q_B, Q_F, \frac{1}{q}, \frac{1}{q} \right) \, , \quad q = \textrm{e}^{\textrm{i} g_s} . \nonumber \\ \end{aligned}$$

On the other hand, one has $Z_{{\mathbb {F}}_0}^\text {NS}\left( X^S, Q_B^S, Q_F^S, g_s \right)$ which is the NS phase of the refined topological vertex, (A.10), (A.12), (A.13),

A.16 $$\begin{aligned}{} & {} Z_{{\mathbb {F}}_0}^\text {NS}\left( X^S, Q_B^S, Q_F^S, g_s \right) = \lim _{p^S \rightarrow 1} Z^{\text {t-brane}}_{{\mathbb {F}}_0} \left( - \frac{\sqrt{p^S} }{\sqrt{Q_F^S} X^S}, Q_B^S, Q_F^S, \frac{1}{p^S}, \frac{1}{q^S} \right) \, , \nonumber \\{} & {} \quad q^S = \textrm{e}^{\textrm{i} \frac{\left( 2 \pi \right) ^2}{g_s}} \, , \end{aligned}$$

which is perturbative in the inverse string coupling $g_s^{-1}$. The exponentiated Kähler parameters $X, Q_B, Q_F$ and their NS counterparts $X^S, Q_B^S, Q_F^S$ are related by powers of $2 \pi / g_s$,

A.17 $$\begin{aligned} A^S =A^{2 \pi / g_s} \, , \quad A = \left( A^S\right) ^{g_s / 2 \pi } \, , \end{aligned}$$

where *A* stands for either one of $X, Q_B, Q_F$. The “polynomial” part $Z^\text {(p)}\left( X, g_s \right)$ is not obtained from the topological vertex but is given by [83, eq. 4.10]^28^

A.18 $$\begin{aligned} Z_{{\mathbb {F}}_0}^\text {(p)} \left( X, g_s \right)= & {} \exp \left\{ - \frac{\textrm{i}}{2} \frac{1}{g_s} \left[ \log ^2 \left( X \right) - \textrm{i} 2 \pi \log \left( X \right) \right] + \frac{\log \left( X \right) }{2} \right\} \nonumber \\= & {} \exp \left\{ - \frac{\textrm{i}}{2} \frac{g_s}{\left( 2 \pi \right) ^2} \left[ \log ^2 \left( X^S \right) + \textrm{i} 2 \pi \log \left( X^S \right) \right] - \frac{\log \left( X^S \right) }{2} \right\} \, ,\nonumber \\ \end{aligned}$$

which explains all of the functions and symbols appearing in (A.14).

One can see that the GV and NS instanton parts are given as series expansions in $Q_B$ and $Q_B^S$, respectively,

A.19 $$\begin{aligned}{} & {} Z_{{\mathbb {F}}_0}^\text {GV, inst} \left( X, Q_B, Q_F, g_s \right) \nonumber \\{} & {} \quad = 1 - \left[ \frac{ Q_1 \left( 2 Q_F Q_1 - Q_F - 1 \right) }{\left( q - 1 \right) \left( Q_F - 1 \right) ^2 \left( Q_1 - 1 \right) \left( Q_F Q_1 - 1 \right) }\right] Q_B + {\mathcal {O}}\left( Q_B^2\right) \nonumber \\{} & {} Z_{{\mathbb {F}}_0}^\text {NS, inst}\left( X^S, Q_B^S, Q_F^S, g_s \right) \nonumber \\{} & {} \quad = 1 - \left[ \frac{\left( q^S\right) ^2 Q_1^S \left( q^S \left( q^S + 1 \right) Q_F^S Q_1^S - Q_F^S - 1\right) }{(q^S-1) (Q_F^S - q^S) (q^S Q_F^S-1) (q^S Q_1^S-1) (q^S Q_F^S Q_1^S-1)}\right] Q_B^S + {\mathcal {O}} \left( \left( Q_B^S\right) ^2\right) \nonumber \\ \end{aligned}$$

where $$Q_1 = \sqrt{q} / \sqrt{Q_F} X$$ and $$Q_1^S = - 1 / \sqrt{Q_F^S} X^S$$.

By looking at the full expression (A.14), we see that two distinct four-dimensional limits can be implemented: one in which $Z^{\textrm{GV}}_{{{\mathbb {F}}}_0}$ survives, and another in which $Z^\textrm{NS}_{{{\mathbb {F}}}_0}$ survives. Using the TS/ST correspondence [52] one can show that at the level of the operator theory the first limit makes contact with (3.28) and (3.29). The second limit instead gives (3.24) and (3.25). A more detailed explanation of this phenomenon can be found in [48, 49, 51]. In this paper, our primary focus is directed toward the first limit.

#### The 1-Loop Part in Terms of Faddeev’s Quantum Dilogarithm

To take the 4d limit of the partition function (A.14), we first need to rewrite the 1-loop part and combine $Z_{{\mathbb {F}}_0}^\text {GV, 1-loop}$ and $Z_{{\mathbb {F}}_0}^\text {NS, 1-loop}$ into a single function. From (A.12), (A.15) and (A.16) we have

A.20 $$\begin{aligned} \begin{aligned} Z^\text {GV, 1-loop}_{{\mathbb {F}}_0}\left( X, Q_F, q \right)&= \left( \frac{\sqrt{q}}{\sqrt{Q_F} X} ; q \right) _\infty \left( \frac{\sqrt{q} \sqrt{Q_F}}{X} ; q \right) _\infty \\&= \left[ \left( \frac{1}{\sqrt{q} \sqrt{Q_F} X} ; \frac{1}{q} \right) _\infty \left( \frac{\sqrt{Q_F}}{\sqrt{q} X} ; \frac{1}{q} \right) _\infty \right] ^{-1}, \\ Z^\text {NS, 1-loop}_{{\mathbb {F}}_0}\left( X^S, Q_F^S, q^S \right)&= \left( - \frac{q^S}{\sqrt{Q_F^S} X^S} ; q^S \right) _\infty \left( - \frac{q^S \sqrt{Q_F^S}}{X^S} ; q^S \right) _\infty \\&= \left[ \left( - \frac{1}{\sqrt{Q_F^S} X^S} ; \frac{1}{q^S} \right) _\infty \left( - \frac{\sqrt{Q_F^S}}{X^S} ; \frac{1}{q^S} \right) _\infty \right] ^{-1}, \end{aligned} \end{aligned}$$

where $\left( a ; q \right) _\infty$ is the *q*-Pochhammer symbol. The product of $Z^\text {GV, 1-loop}_{{\mathbb {F}}_0}$ and $Z^\text {NS, 1-loop}_{{\mathbb {F}}_0}$ can then be written as a product of quantum dilogarithms.

Faddeev’s quantum dilogarithm can be defined by [151] [152, eq. 2] [54, eqs. 157, 158]

A.21 $$\begin{aligned} \Phi _b \left( z\right) = \frac{\left( -\textrm{e}^{\textrm{i}\pi b^2 + 2\pi b z}; \textrm{e}^{\textrm{i}2 \pi b^2}\right) _\infty }{\left( -\textrm{e}^{- \textrm{i}\pi b^{-2} + 2 \pi b^{-1} z} ; \textrm{e}^{ - \textrm{i}2 \pi b^{-2} }\right) _\infty } \, , \quad \textrm{Im}\left( b^2\right) > 0 \, , \end{aligned}$$

and it can be extended to all $b^2 \in {\mathbb {C}}\setminus {\mathbb {R}}_{\leqslant 0}$. The quantum dilogarithm^29^ is a meromorphic function of *z* with zero’s along the negative imaginary axis and poles along the positive imaginary axis when $b > 0$ [54, eq. 160], and with an essential singularity at infinity [152, p. 2]. If one takes $b^2 = 2\pi / g_s > 0$, one has

A.22 $$\begin{aligned} q = \textrm{e}^{\textrm{i} g_s} = \textrm{e}^{\textrm{i} 2 \pi b^{-2}} \, , \quad q^S = \textrm{e}^{\textrm{i} \left( 2 \pi \right) ^2 / g_s} = \textrm{e}^{\textrm{i} 2 \pi b^2} \, , \end{aligned}$$

and defining $z_+$ and $z_-$ by

A.23 $$\begin{aligned} \frac{\left( \sqrt{Q_F}\right) ^{\mp 1}}{X} = \textrm{e}^{2 \pi b^{-1} z_\pm - \textrm{i}\pi } = \left( \frac{\left( \sqrt{Q_F^S}\right) ^{\mp 1}}{X^S} \right) ^{1/b^2} \, , \end{aligned}$$

one gets for the 1-loop part of the partition function (A.14)

A.24 $$\begin{aligned} Z_{{\mathbb {F}}_0}^\text {1-loop} = Z^\text {GV, 1-loop}_{{\mathbb {F}}_0} Z^\text {NS, 1-loop}_{{\mathbb {F}}_0} = \Phi _b \left( z_+\right) \Phi _b \left( z_-\right) \, . \end{aligned}$$

A useful property of Faddeev’s quantum dilogarithm $\Phi _b\left( z\right)$ is that it is invariant under inversion of *b* with *z* kept fixed,

A.25 $$\begin{aligned} \Phi _b \left( z \right) = \Phi _{1/b} \left( z \right) \, . \end{aligned}$$

In a physics language, this corresponds to a sort of S-duality^30^, since it inverts the string coupling $g_s$.

An integral representation of the quantum dilogarithm which will be useful for us is [156, eq. A.2]

A.26 $$\begin{aligned} \log \left( \Phi _b\left( z\right) \right)= & {} - \frac{\textrm{i}}{2 \pi b^2} \textrm{Li}_2 \left( -\textrm{e}^{2\pi b z}\right) - \textrm{i} \int \limits _0^{+\infty } \frac{\textrm{d}t}{1 + \textrm{e}^{2\pi t}} \log \left( \frac{1 + \textrm{e}^{2 \pi b z} \textrm{e}^{- 2 \pi b^2 t}}{1 + \textrm{e}^{2 \pi b z} \textrm{e}^{2 \pi b^2 t}}\right) \nonumber \\= & {} - \frac{\textrm{i}}{2 \pi } b^2 \textrm{Li}_2 \left( -\textrm{e}^{2\pi b^{-1} z }\right) - \textrm{i} \int \limits _0^{+\infty } \frac{\textrm{d}t}{1 + \textrm{e}^{2\pi t}} \log \left( \frac{1 + \textrm{e}^{2 \pi b^{-1} z} \textrm{e}^{- 2 \pi b^{-2} t}}{1 + \textrm{e}^{2 \pi b^{-1} z} \textrm{e}^{2 \pi b^{-2} t}}\right) ,\nonumber \\ \end{aligned}$$

where the second representation follows from the inversion symmetry of the quantum dilogarithm (A.25). It is this second integral representation which will be useful in taking the 4d limit.

### A Four-Dimensional Limit

Consider the following reparametrization of the Kähler parameters [48, p. 5]

A.27 $$\begin{aligned} X = \exp \left( g_s x \right) \, , \quad Q_B = g_s^4 t \, , \quad Q_F = \exp \left( 2 g_s \textrm{i} \sigma \right) \, , \end{aligned}$$

where as before $q = \exp \left( \textrm{i} g_s \right)$ and $q^S = \exp \left( \textrm{i} \left( 2 \pi \right) ^2 / g_s \right)$. The four-dimensional limit is then $g_s \rightarrow 0$ with *x*, *t* and $\sigma$ and kept fixed. In terms of more familiar gauge theoretic quantities, one has

A.28 $$\begin{aligned} x = \frac{y}{2 \epsilon } \, , \quad t = \left( \frac{\Lambda }{\epsilon }\right) ^4 \, , \quad \sigma = \textrm{i} \frac{a}{ 2 \epsilon } \, , \end{aligned}$$

where $\epsilon$ corresponds to the $\Omega$-background parameter in the self-dual phase, $\Lambda$ is the instanton counting parameter, and *a* is the Coulomb branch parameter or equivalently the A-period of the underlying Seiberg–Witten geometry. The parameter *y* corresponds to the position of the defect. Note also that the variable *x* is called *q* in the main text.

The instanton part of the type II defect is obtained as the $g_s \rightarrow 0$ limit of the GV instanton part (A.19) of the topological string partition function in the parametrization (A.27),

A.29 $$\begin{aligned}{} & {} Z_{\textrm{inst}}^\text {II}\left( x, t, \sigma \right) = \lim _{g_s \rightarrow 0} Z_{{\mathbb {F}}_0}^\text {GV, inst} \left( \exp \left( g_s x \right) , g_s^4 t, \exp \left( 2 g_s \textrm{i}\sigma \right) , g_s \right) = 1 - \left[ \frac{\widetilde{x}}{2 \sigma ^2 \left( \widetilde{x}^2 - \sigma ^2 \right) }\right] t \nonumber \\{} & {} \quad + \left[ \frac{\widetilde{x} \left( \widetilde{x} + 1 \right) ^2 - \widetilde{x} \left( 10 \widetilde{x}^2 + 19 \widetilde{x} + 10 \right) \sigma ^2 + \left( 8 \widetilde{x}^2 + 30 \widetilde{x} + 9 \right) \sigma ^4}{4 \sigma ^4 \left( 4 \sigma ^2 - 1 \right) ^2 \left( \widetilde{x}^2 - \sigma ^2\right) \left( \left( \widetilde{x} + 1 \right) ^2 - \sigma ^2\right) } \right] t^2 +{\mathcal {O}}\left( t^3\right) ~, \end{aligned}$$

where we used for the sake of readability

A.30 $$\begin{aligned} \widetilde{x} = \textrm{i} x + \frac{1}{2}~. \end{aligned}$$

We checked (A.29) against [17, eqs A.3-A.13] up to and including order $t^2$, and found perfect agreement^31^. On the other hand, one can see that the NS instanton part in (A.19) vanishes in the same four-dimensional limit.

In the 4d limit, the polynomial part (A.18) reduces to

A.31 $$\begin{aligned} Z^\text {II,(p)}\left( x\right) = \lim _{g_s \rightarrow 0} Z^\text {(p)} \left( \exp \left( g_s x \right) , g_s \right) = \exp \left( - \pi x \right) = \exp \left( - \pi \frac{y}{2 \epsilon } \right) . \qquad \end{aligned}$$

Looking at the 1-loop part (A.23) and (A.24) and the reparametrization (A.27), one can see that

A.32 $$\begin{aligned} - \textrm{e}^{2 \pi b^{-1} z_\pm } = \textrm{e}^{- g_s \left( x \pm \textrm{i}\sigma \right) }, \end{aligned}$$

and using the second integral representation of Faddeev’s quantum dilogarithm (A.26) gives

A.33 $$\begin{aligned} \log \left( \Phi _b\left( z_\pm \right) \right) = - \textrm{i} \frac{\textrm{Li}_2 \left( \textrm{e}^{- g_s \left( x \pm \textrm{i}\sigma \right) }\right) }{g_s} - \textrm{i} \int \limits _0^{+\infty } \frac{\textrm{d}t}{1 + \textrm{e}^{2\pi t}} \log \left( \frac{1 - \textrm{e}^{- g_s \left( x \pm \textrm{i}\sigma \right) } \textrm{e}^{- g_s t}}{1 - \textrm{e}^{- g_s \left( x \pm \textrm{i}\sigma \right) } \textrm{e}^{g_s t}}\right) . \nonumber \\ \end{aligned}$$

The expansion of for $g_s \rightarrow 0$ from above

A.34 $$\begin{aligned}{} & {} - \textrm{i} \frac{\textrm{Li}_2 \left( \textrm{e}^{- g_s \left( x \pm \textrm{i}\sigma \right) }\right) }{g_s} = - \textrm{i} \frac{\pi ^2}{6} \frac{1}{g_s} - \textrm{i} \left( x \pm \textrm{i}\sigma \right) \log \left( g_s\right) \nonumber \\{} & {} \qquad - \textrm{i} \left( x \pm \textrm{i}\sigma \right) \left[ \log \left( x \pm \textrm{i}\sigma \right) - 1 \right] + {\mathcal {O}}\left( g_s \right) , \end{aligned}$$

A.35 $$\begin{aligned}{} & {} \qquad - \textrm{i} \int \limits _0^{+\infty } \frac{\textrm{d}t}{1 + \textrm{e}^{2\pi t}} \log \left( \frac{1 - \textrm{e}^{- g_s \left( x \pm \textrm{i}\sigma \right) } \textrm{e}^{- g_s t}}{1 - \textrm{e}^{- g_s \left( x \pm \textrm{i}\sigma \right) } \textrm{e}^{g_s t}}\right) \nonumber \\{} & {} \quad = - \textrm{i} \int \limits _0^{+\infty } \frac{\textrm{d}t}{1 + \textrm{e}^{2\pi t}} \log \left( \frac{x \pm \textrm{i}\sigma + t}{x \pm \textrm{i}\sigma - t}\right) + {\mathcal {O}}\left( g_s\right) , \end{aligned}$$

The divergent terms in (A.34) can be dealt with properly in the context of the open TS/ST correspondence [140], but we will simply drop them. By using the integral representation of the Gamma function, we get [157, p. 8]

A.36 $$\begin{aligned}{} & {} - \textrm{i} \int \limits _0^{+\infty } \frac{\textrm{d}t}{1 + \textrm{e}^{2\pi t}} \log \left( \frac{x \pm \textrm{i}\sigma + t}{x \pm \textrm{i}\sigma - t}\right) = - 2 \int \limits _0^{+\infty } \frac{\textrm{d}t}{1 + \textrm{e}^{2\pi t}} \arctan \left( \frac{t}{- \textrm{i} \left( x \pm \textrm{i}\sigma \right) }\right) \nonumber \\{} & {} \quad \quad = \textrm{i} \left( x \pm \textrm{i}\sigma \right) \log \left( - \textrm{i} \left( x \pm \textrm{i}\sigma \right) \right) - \textrm{i} \left( x \pm \textrm{i}\sigma \right) - \frac{\log \left( 2\pi \right) }{2} + \log \Gamma \left( - \textrm{i} \left( x \pm \textrm{i}\sigma \right) + \frac{1}{2} \right) , \nonumber \\ \end{aligned}$$

where $\log \Gamma$ is the log Gamma function. Hence, we get

A.37 $$\begin{aligned} \lim _{g_s \rightarrow 0} \log \left( \Phi _b\left( z_\pm \right) \right) = \log \Gamma \left( - \textrm{i} \left( x \pm \textrm{i}\sigma \right) + \frac{1}{2} \right) - \frac{\log \left( 2\pi \right) }{2} + \frac{\pi }{2} \left( x \pm \textrm{i}\sigma \right) \, , \nonumber \\ \end{aligned}$$

and combining with (A.31) gives the perturbative part of the type II defect partition function

A.38 $$\begin{aligned} \begin{aligned} Z^\text {II}_{\textrm{pert}}\left( x, \sigma \right)&= \lim _{g_s \rightarrow 0} Z_{{\mathbb {F}}_0}^\text {(p)} \left( \exp \left( g_s x \right) , g_s \right) Z_{{\mathbb {F}}_0}^\text {1-loop} \left( \exp \left( g_s x \right) , \exp \left( 2 g_s \textrm{i}\sigma \right) , g_s \right) \\&= \frac{\Gamma \left( - \textrm{i} \left( x - \textrm{i}\sigma \right) + \frac{1}{2} \right) \Gamma \left( - \textrm{i} \left( x + \textrm{i}\sigma \right) + \frac{1}{2} \right) }{2 \pi } \, . \end{aligned} \end{aligned}$$

The product of the perturbative (A.38) and instanton (A.29) (A.30) parts gives us then the complete partition function for the type II defect in 4d, ${\mathcal {N}} = 2$, $\textrm{SU}\left( 2\right)$ super Yang–Mills^32^,

A.39 $$\begin{aligned} Z^\text {II}\left( x, t, \sigma \right){} & {} = \exp \left( \frac{\textrm{i}}{2} x \log \left( t \right) \right) \Gamma \left( - \textrm{i} x - \sigma + \frac{1}{2} \right) \nonumber \\{} & {} \quad \Gamma \left( - \textrm{i} x + \sigma + \frac{1}{2} \right) Z_{\textrm{inst}}^\text {II}\left( x, t, \sigma \right) ~. \end{aligned}$$

Note that we use a slightly different notation compared to the main text: what we call *x* here is called *q* elsewhere.

## From the Matrix Model Identity to the Eigenfunction Identity

In this appendix, we argue for the equivalence between the identities (6.5) and (6.6)^33^. Our strategy is similar to the one used in the context of ABJM theory, see, e.g., [158] and reference therein.

Let us start by writing (6.5) as

B.1 $$\begin{aligned}{} & {} \int \limits _{ {{\mathbb {R}}}+\textrm{i}\sigma _*}{\textrm{d}\sigma } {\tan \left( 2 \pi \sigma \right) \over \left( 2 \cos (2 \pi \sigma )\right) ^N} \left( Z^{\textrm{II}}_{\textrm{tot}}\left( q, t, \sigma \right) +Z^\textrm{II}_{\textrm{tot}}\left( -q-{\textrm{i}\over 2}, t, \sigma +{1\over 2} \right) \right) \nonumber \\{} & {} \quad = \textrm{i} {2^{11/12}\sqrt{\pi }{ t^{3/16} }\over \textrm{e}^{3\zeta '(-1)}\textrm{e}^{4 \sqrt{t}}(4\pi )^N}\int \limits _{{\mathbb {R}}} {\textrm{d}x} \ \textrm{e}^{-\textrm{i}{2} q x} \textrm{e}^{- 4 t^{1/4} \cosh x + {x\over 2}} \Psi _N(\textrm{e}^{{x}}, t), \end{aligned}$$

where we have absorbed the $(-1)^N$ into a shift of $\sigma$, and $\sigma _*$ is a strictly positive number which guarantees that the integration contour on the left-hand side of (B.1) does not hit the poles of $Z^{\textrm{II}}_{\textrm{tot}}$. This is the case if $0< \sigma _* < |\textrm{Re}(q)|\ne 0.$ If $\textrm{Re}(q)=0$, one can take $\sigma _*$ to be any strictly positive number as in footnote 12. For the sake of notation, let us define

B.2 $$\begin{aligned} f(N)&= \textrm{i}{2^{11/12}\sqrt{\pi }{ t^{3/16} }\over \textrm{e}^{3\zeta '(-1)}\textrm{e}^{4 \sqrt{t}}(4\pi )^N}\int \limits _{{\mathbb {R}}} {\textrm{d}x} \ \textrm{e}^{-\textrm{i}{2} q x} \textrm{e}^{- 4 t^{1/4} \cosh x+ {x\over 2}} \Psi _N(\textrm{e}^{{x}}, t) \, , \end{aligned}$$

B.3 $$\begin{aligned} g(\sigma )&=Z^{\textrm{II}}_{\textrm{tot}}\left( q, t, \sigma \right) + Z^\textrm{II}_{\textrm{tot}}\left( -q-{\textrm{i}\over 2}, t, \sigma + {1\over 2} \right) \, , \end{aligned}$$

so that (B.1) becomes

B.4 $$\begin{aligned} f\left( N\right)= & {} \int \limits _{\mathbb {R}} \textrm{d}\sigma \frac{\tan \left( 2 \pi \left( \sigma + \textrm{i} \sigma _* \right) \right) }{\left[ 2 \cos \left( 2 \pi \left( \sigma + \textrm{i} \sigma _* \right) \right) \right] ^N} g\left( \sigma + \textrm{i} \sigma _* \right) \nonumber \\= & {} \int \limits _{-1/2}^{1/2} \textrm{d} \sigma \frac{\tan \left( 2 \pi \left( \sigma + \textrm{i} \sigma _* \right) \right) }{\left[ 2 \cos \left( 2 \pi \left( \sigma + \textrm{i} \sigma _* \right) \right) \right] ^N} \sum _{k \in {\mathbb {Z}}} g\left( \sigma + \textrm{i} \sigma _* + k \right) ~. \end{aligned}$$

Note that the second equality in (B.4) assumes some good analytic properties of *g*, for instance, *g* is such that the sum over *n* on the right-hand side of (B.4) is convergent. This is the case for (B.2). Furthermore, it is part of our conjecture that the function $\sum _{k \in {\mathbb {Z}}} g\left( \sigma + \textrm{i} \sigma _* + k \right)$ is not only well defined but also an entire function of $\sigma$. Hence, we are free to deform the integration path in (B.4) to any path ${\mathcal {C}}_{ \left\{ -1/2, 1/2\right\} }$, beginning at $\sigma = -1/2$ and ending at $\sigma = 1/2$, as long as we don’t cross the poles coming from the tangent when $\sigma + \textrm{i} \sigma _* \in {\mathbb {Z}} / 2 + 1 / 2$. Consider then the change of variables given by

B.5 $$\begin{aligned} \mu : {\mathcal {C}}_{ \left\{ -\frac{1}{2}, \frac{1}{2} \right\} } \rightarrow \left] - \pi , \pi \right[: \sigma \mapsto \mu \left( \sigma \right) = - \textrm{i} \log \left[ \frac{\cos \left( 2 \pi \left( \sigma + \textrm{i} \sigma _* \right) \right) }{2 \pi }\right] , \end{aligned}$$

which is well defined for a suitable choice of ${\mathcal {C}}_{ \left\{ -1/2, 1/2\right\} }$. Then, we have for the integral that

B.6 $$\begin{aligned} \frac{1}{2 \pi }\int \limits _{- \pi }^{\pi } \textrm{d} \mu \exp \left( - \textrm{i} \mu N \right) \cdots = - \textrm{i} \left( 2 \pi \right) ^N \int \limits _{-1/2}^{1/2} \textrm{d} \sigma \frac{\tan \left( 2 \pi \left( \sigma + \textrm{i} \sigma _* \right) \right) }{\left[ \cos \left( 2 \pi \left( \sigma + \textrm{i} \sigma _* \right) \right) \right] ^N} \cdots ~,\qquad \end{aligned}$$

and hence, we can rewrite (B.4) as

B.7 $$\begin{aligned} - \textrm{i} \left( 4 \pi \right) ^N f \left( N \right) = \frac{1}{2 \pi } \int \limits _{- \pi }^{\pi } \textrm{d} \mu \exp \left( - \textrm{i} \mu N \right) \sum _{k \in {\mathbb {Z}}}g\left( \sigma \left( \mu \right) + \textrm{i} \sigma _* + k \right) , \end{aligned}$$

where $\sigma \left( \mu \right)$ is the inverse of (B.5). Note that the integral on the right-hand side gives the *N*-th Fourier coefficient of the function $\sum _{k \in {\mathbb {Z}}} g\left( \sigma \left( \mu \right) + \textrm{i} \sigma _* + k \right)$. The full Fourier series leads then to

B.8 $$\begin{aligned}{} & {} - \textrm{i} \sum _{N \in {\mathbb {N}}} \left( 4 \pi \right) ^N \exp \left( \textrm{i} \nu N\right) f \left( N\right) = \sum _{N \in {\mathbb {Z}}} \frac{\exp \left( \textrm{i} \nu N \right) }{2 \pi } \nonumber \\{} & {} \qquad \int \limits _{- \pi }^{\pi } \textrm{d} \mu \exp \left( - \textrm{i} \mu N \right) \sum _{k \in {\mathbb {Z}}} g\left( \sigma \left( \mu \right) + \textrm{i} \sigma _* + k \right) \nonumber \\{} & {} \quad = \sum _{k \in {\mathbb {Z}}} g\left( \sigma \left( \nu \right) + \textrm{i} \sigma _* + k \right) . \end{aligned}$$

where the last equality holds whenever the Fourier series on the previous line is convergent. We expect this to be true in our case even though we do not have a rigorous proof. We also used that $f(-N)=0$ for $N \in {{\mathbb {N}}}\setminus \left\{ 0 \right\}$.

To go in the opposite direction from (B.8) to (B.4), one can apply Cauchy’s residue theorem. Using the notation

B.9 $$\begin{aligned} \kappa = \exp \left( \textrm{i} \nu \right) = \frac{\cos \left( 2 \pi \left( \sigma + \textrm{i} \sigma _* \right) \right) }{2 \pi } \end{aligned}$$

one can multiply both sides in (B.8) by $1/ \kappa ^{ N + 1}$ and integrate over $\kappa$ along an anticlockwise contour of radius 1 centered at the origin to arrive at

B.10 $$\begin{aligned} \begin{aligned} \left( 2 \pi \right) \left( 4 \pi \right) ^N f \left( N \right)&= \oint \frac{\textrm{d} \kappa }{\kappa ^{N+1}} \sum _{k \in {\mathbb {Z}}} g\left( \sigma \left( - \textrm{i} \log \left( \kappa \right) \right) + \textrm{i} \sigma _* + k \right) \\&= \textrm{i} \int \limits _{- \pi }^{\pi } \textrm{d} \nu \exp \left( - \textrm{i} \nu N \right) \sum _{k \in {\mathbb {Z}}} g\left( \sigma \left( \nu \right) + \textrm{i} \sigma _* + k \right) \end{aligned}\qquad \end{aligned}$$

which is equivalent to (B.7) and hence by (B.6) to (B.4). We can conclude that (B.4) and (B.8) are indeed equivalent, provided *f* and *g* have good analytic properties which we assume to be the case.

## Elliptic Integrals

For the elliptic integrals, we follow the conventions of *Wolfram Mathematica* [159–162]. The notation in [131, pp. 8-10] is slightly different and we denote their elliptic integrals with a tilde. In particular, we have the normal or incomplete elliptic integral of the first kind for $k^2 \in {\mathbb {R}}$, $-\pi / 2< \phi < \pi / 2$ and $k^2 \sin ^2\left( \phi \right) < 1$, [160, 131, eq. 110.02],

C.1 $$\begin{aligned} \textrm{F}\left( \phi | k^2 \right) = \int \limits _0^\phi \frac{\textrm{d} \theta }{\sqrt{1 - k^2 \sin ^2\left( \theta \right) }} = \widetilde{F}\left( \phi , k \right) , \end{aligned}$$

the normal or incomplete elliptic integral of the second kind for $k^2 < 1$ and $-\pi / 2< \phi < \pi / 2$, [161, 131, eq. 110.03],

C.2 $$\begin{aligned} \textrm{E}\left( \phi | k^2 \right) = \int \limits _0^\phi \textrm{d} \theta \sqrt{1 - k^2 \sin ^2\left( \theta \right) } = \widetilde{E}\left( \phi , k \right) , \end{aligned}$$

and the normal or incomplete elliptic integral of the third kind for $k^2, \alpha ^2 \in {\mathbb {R}}$, $-\pi / 2< \phi < \pi / 2$ and $k^2 \sin ^2\left( \phi \right) < 1$, [162, 131, eq. 110.04],

C.3 $$\begin{aligned} \Pi \left( \alpha ^2; \phi | k^2\right) = \int \limits _0^\phi \frac{\textrm{d} \theta }{\left( 1-\alpha ^2 \sin ^2\left( \theta \right) \right) \sqrt{1 - k^2 \sin ^2\left( \theta \right) }} = \widetilde{\Pi }\left( \phi , \alpha ^2, k\right) . \end{aligned}$$

The complete elliptic integrals are obtained by taking $\phi = \pi / 2$,

C.4 $$\begin{aligned} \textrm{K}\left( k^2\right) =\textrm{F}\left( \frac{\pi }{2} | k^2 \right) , \quad \textrm{E}\left( k^2\right) =\textrm{E}\left( \frac{\pi }{2} | k^2 \right) , \quad \Pi \left( \alpha ^2 | k^2\right) =\Pi \left( \alpha ^2; \frac{\pi }{2} | k^2\right) . \nonumber \\ \end{aligned}$$

The complete elliptic integrals of the first and second kind are analytic on ${\mathbb {C}}$ apart from a branch cut along the positive real line for $k^2 \geqslant 1$, and the complete elliptic integral of the third kind is analytic on ${\mathbb {C}}^2$ apart from similar branch cuts for $k^2, \alpha ^2 \geqslant 1$ [159, 161, 162].

We also need a Jacobi elliptic function $\textrm{sn}$ which is an inverse of the incomplete elliptic integral of the first kind [131, p. 18],

C.5 $$\begin{aligned} \textrm{sn} \left( v | k^2 \right) \, , \quad \textrm{sn} \left( \textrm{F}\left( \phi | k^2 \right) | k^2 \right) = \sin \left( \phi \right) \, , \end{aligned}$$

which is sometimes called the sine amplitude.

## Footnotes

1 A rigorous derivation of some of these results can be found in [28]. A q-perturbative approach to connection coefficients is also discussed in [29, 30], see also [20].

2 We refer to (1.1) as a Fermi gas operator, since it corresponds to the density matrix of an ideal Fermi gas in the external potential $8 t^{1/4} \cosh \left( x\right)$.

3 This is in line with the generic expectation that matrix model averages are related to partitions functions in the presence of D-branes [41, 64–69].

4 Following [17, sec. 2.3.3], the two-dimensional ${\mathcal {N}}=(2,2)$ theory here consists of 2 free chiral multiplets living on a disk. See also [70–72].

5 The parameter *y* corresponds to the insertion of the defect on the Riemann sphere *C* in the 6d, ${\mathcal {N}} = (2,0)$ theory on ${\mathbb {R}}^4 \times C$, and it is a position in that sense. From the perspective of the two-dimensional ${\mathcal {N}} = (2, 2)$ chiral multiplets, it corresponds to the twisted mass for the *U*(1) flavor symmetry of the chirals, see, e.g., [69].

6 Both surface defects are defined by coupling the 4d theory to the same 2d gauged linear sigma model, but in different phases of the 2d theory. The transition between these phases explains the Fourier transform relation between their vacuum expectation values as explained in [19]. We thank Saebyeok Jeong for pointing that out.

7 We would like to thank A. Neitzke for useful discussions on this point.

8 Since we consider gauge theories of rank 1 this is equivalent to a type III defect as defined in [17, Sect. 2.3.3].

9 Beside numerical and physical evidence, we are not aware of a rigorous mathematical proof of this fact, see [90, 91] for related discussions and proofs in other phases of the $\Omega$-background.

10 As before, we are following the terminology of [17, Sect. 2.3.3]. We also note that, since we are considering only rank 1 theories, type I and type III defects as in [17, Sect. 2.3.3] are identified.

11 The magnetic frame can be obtained from the usual electric frame by an S-duality transformation. It allows us to study the behavior of the gauge theory near the magnetic monopole point. See Sect. 5 and [89] for more details.

12 One can take $\sigma _* = \textrm{arccosh} (2 \pi ) / 2 \pi$ as in [49] or any other $\sigma ^* > 0$ as long as the integral over $\sigma$ in (3.15) does not hit the poles of the integrand.

13 It is possible to analytically continue the results to non-integer values of *N* [49, 95].

14 It is also possible to take $\hbar$ and $m_{{{\mathbb {F}}}_0}$ imaginary within some range. In this case, one still has the trace class property, but the spectrum may no longer be real [117, 118].

15 We would like to thank M. Mariño and N. Orantin for useful discussions on this point.

16 The potential of the one-dimensional ideal Fermi gas is $- \log \left( v\left( x \right) \right) = V\left( \textrm{e}^x \right) / \epsilon$.

17 See Appendix C for our conventions on elliptic integrals.

18 The planar resolvent is defined and computed explicitly further on in Sect. 4.1.

19 The statement in [131, eq. 117.02] is for $\alpha ^2 \in {\mathbb {R}} \setminus {\mathcal {H}}_{k^2}$, but when $0< k < 1$, this can be extended to $\alpha ^2 \in {\mathbb {C}} \setminus {\mathcal {H}}_{k^2}$ by making use of the identity theorem.

20 Here we take $z> \texttt {g}^{-1} > 1$ to simplify the discussion and to make contact with the standard formulas in the literature [68, 78]. Hence, we are outside the branch cut on the matrix model side.

21 For sake of notation we omitted the proportionality factor $2^{1/12} \textrm{e}^{ 3\zeta '(-1)} \left( \Lambda / \epsilon \right) ^{-1/4} \textrm{e}^{4 \left( \Lambda /\epsilon \right) ^2}$

22 Hence, we have $a_D = \lambda$.

23 For the part involving only the free energies the all order results follows from [48, 49].

24 This also suggests that the defect partition function $Z^{\textrm{II}}\left( q, t,\sigma \right)$ for this theory has an infinite radius of convergence in *t* for fixed $\sigma$ and *q* which are away from the poles. For the self-dual partition function without defect, this was rigorously proved in [90], see also [91] for more recent developments.

25 Upon using the Matone relation (5.17) and careful treatment of the branches.

26 One can reach this precision with Hankel matrices of size $M = 2^{9} = 512$.

## References

[1] Seiberg, N., Witten, E.: Monopoles, duality and chiral symmetry breaking in N=2 supersymmetric QCD. Nucl. Phys. B **431**, 484–550 (1994). 10.1016/0550-3213(94)90214-3. [arXiv:hep-th/9408099]

[2] Seiberg, N., Witten, E.: Electric: magnetic duality, monopole condensation, and confinement in N=2 supersymmetric Yang-Mills theory. Nucl. Phys. B **426**, 19–52 (1994). 10.1016/0550-3213(94)90124-4. [arXiv:hep-th/9407087]

[3] Moore, G.W., Nekrasov, N., Shatashvili, S.: Integrating over Higgs branches. Commun. Math. Phys. **209**, 97–121 (2000). 10.1007/PL00005525. [arXiv:hep-th/9712241]

[4] Losev, A., Nekrasov, N., Shatashvili, S.L.: Issues in topological gauge theory. Nucl. Phys. B **534**, 549–611 (1998). 10.1016/S0550-3213(98)00628-2. [arXiv:hep-th/9711108]

[5] Nekrasov, N.A.: Seiberg-Witten prepotential from instanton counting. Adv. Theor. Math. Phys. **7**, 831–864 (2004). 10.4310/ATMP.2003.v7.n5.a4. [arXiv:hep-th/0206161]

[6] Nekrasov, N., Okounkov, A.: Seiberg-Witten theory and random partitions. Prog. Math. **244**, 525–596 (2006). 10.1007/0-8176-4467-9_15. [arXiv:hep-th/0306238]

[7] Nekrasov, N. A., Shatashvili, S. L.: Quantization of Integrable Systems and Four Dimensional Gauge Theories, in *XVIth International Congress On Mathematical Physics*, pp. 265–289, World Scientific, (2010). 10.1142/9789814304634_0015. arXiv:0908.4052

[8] Mironov, A., Morozov, A.: Nekrasov Functions and Exact Bohr-Sommerfeld Integrals. JHEP **1004**, 040 (2010). 10.1007/JHEP04(2010)040. [arXiv:0910.5670]

[9] Mironov, A., Morozov, A.: Nekrasov functions from exact BS periods: the case of SU(N). J. Phys. A **43**, 195401 (2010). 10.1088/1751-8113/43/19/195401. [arXiv:0911.2396]

[10] Zenkevich, Y.: Nekrasov prepotential with fundamental matter from the quantum spin chain. Phys. Lett. B **701**, 630–639 (2011). 10.1016/j.physletb.2011.06.030. [arXiv:1103.4843]

[11] Nekrasov, N., Rosly, A., Shatashvili, S.: Darboux coordinates, Yang-Yang functional, and gauge theory. Nucl. Phys. B, Proc. Suppl. **216**, 69–93 (2011). 10.1016/j.nuclphysbps.2011.04.150. [arXiv:1103.3919]

[12] Kozlowski, K. K., Teschner, J.: TBA for the Toda chain. In New Trends in Quantum Integrable Systems, pp. 195–219, World Scientific, (2010). 10.1142/9789814324373_0011. arXiv:1006.2906

[13] Alday, L.F., Tachikawa, Y.: Affine SL(2) conformal blocks from 4d gauge theories. Lett. Math. Phys. **94**, 87–114 (2010). 10.1007/s11005-010-0422-4. [arXiv:1005.4469]

[14] Gaiotto, D., Kim, H.-C.: Surface defects and instanton partition functions. JHEP **10**, 012 (2016). 10.1007/JHEP10(2016)012. [arXiv:1412.2781]

[15] Kanno, H., Tachikawa, Y.: Instanton counting with a surface operator and the chain-saw quiver. JHEP **06**, 119 (2011). 10.1007/JHEP06(2011)119. [arXiv:1105.0357]

[16] Gaiotto, D., Moore, G.W., Neitzke, A.: Wall-crossing, Hitchin systems, and the WKB approximation. Adv. Math. **234**, 239–403 (2013). 10.1016/j.aim.2012.09.027. [arXiv:0907.3987]

[17] Sciarappa, A.: Exact relativistic Toda chain eigenfunctions from Separation of Variables and gauge theory. JHEP **10**, 116 (2017). 10.1007/JHEP10(2017)116. [arXiv:1706.05142]

[18] Jeong, S., Lee, N., Nekrasov, N.: Intersecting defects in gauge theory, quantum spin chains, and Knizhnik-Zamolodchikov equations. JHEP **10**, 120 (2021). 10.1007/JHEP10(2021)120. [arXiv:2103.17186]

[19] Jeong, S., Lee, N., Nekrasov, N.: Parallel surface defects, Hecke operators, and quantum Hitchin system, *arXiv e-prints: High Energy Physics - Theory* (4, 2023) , 10.48550/arXiv.2304.04656 [arXiv:2304.04656]

[20] Jeong, S., Nekrasov, N.: Opers, surface defects, and Yang-Yang functional. Adv. Theor. Math. Phys. **24**, 1789–1916 (2020). 10.4310/ATMP.2020.v24.n7.a4. [arXiv:1806.08270]

[21] Jeong, S.: Splitting of surface defect partition functions and integrable systems. Nucl. Phys. B **938**, 775–806 (2019). 10.1016/j.nuclphysb.2018.12.007. [arXiv:1709.04926]

[22] Bonelli, G., Iossa, C., Panea Lichtig, D., Tanzini, A.: Irregular liouville correlators and connection formulae for heun functions. Commun. Math. Phys. **397**, 635–727 (2023). 10.1007/s00220-022-04497-5. arxiv:2201.04491

[23] Piatek, M., Pietrykowski, A.R.: Solving Heun’s equation using conformal blocks. Nucl. Phys. B **938**, 543–570 (2019). 10.1016/j.nuclphysb.2018.11.021. [arXiv:1708.06135]

[24] Alday, L.F., Gaiotto, D., Gukov, S., Tachikawa, Y., Verlinde, H.: Loop and surface operators in N=2 gauge theory and Liouville modular geometry. JHEP **1001**, 113 (2010). 10.1007/JHEP01(2010)113. [arXiv:0909.0945]

[25] Drukker, N., Gomis, J., Okuda, T., Teschner, J.: Gauge theory loop operators and liouville theory. JHEP **1002**, 057 (2010). 10.1007/JHEP02(2010)057. [arXiv:0909.1105]

[26] Grassi, A., Gu, J., Mariño, M.: Non-perturbative approaches to the quantum Seiberg-Witten curve. JHEP **07**, 106 (2020). 10.1007/JHEP07(2020)106. [arXiv:1908.07065]

[27] Grassi, A., Hao, Q., Neitzke, A.: Exact WKB methods in SU(2) N = 1. JHEP **01**, 046 (2022). 10.1007/JHEP01(2022)046. [arXiv:2105.03777]

[28] Lisovyy, O., Naidiuk, A.: Perturbative connection formulas for Heun equations. J. Phys. A **55**, 434005 (2022). 10.1088/1751-8121/ac9ba7. [arXiv:2208.01604]

[29] Hollands, L., Rüter, P., Szabo, R.J.: A geometric recipe for twisted superpotentials. JHEP **12**, 164 (2021). 10.1007/JHEP12(2021)164. [arXiv:2109.14699]

[30] Hollands, L., Kidwai, O.: Higher length-twist coordinates, generalized Heun’s opers, and twisted superpotentials. Adv. Theor. Math. Phys. **22**, 1713–1822 (2018). 10.4310/ATMP.2018.v22.n7.a2. [arXiv:1710.04438]

[31] Ito, K., Kanno, S., Okubo, T.: Quantum periods and prepotential in SU(2) SQCD. JHEP **08**, 065 (2017). 10.1007/JHEP08(2017)065. [arXiv:1705.09120]

[32] Ito, K., Kanno, S., Okubo, T.: Quantum periods and prepotential in SU(2) SQCD. JHEP **08**, 065 (2017). 10.1007/JHEP08(2017)065. [arXiv:1705.09120]

[33] Yan, F.: Exact WKB and the quantum Seiberg-Witten curve for 4d N = 2 pure SU(3) Yang-Mills. Abelianization, JHEP **03**, 164 (2022). 10.1007/JHEP03(2022)164. arXiv:2012.15658

[34] Imaizumi, K.: Quantum periods and TBA equations for SQCD with flavor symmetry. Phys. Lett. B **816**, 136270 (2021). 10.1016/j.physletb.2021.136270. arXiv:2103.02248

[35] Hollands, L., Neitzke, A.: Spectral networks and Fenchel-Nielsen coordinates. Lett. Math. Phys. **106**, 811–877 (2016). 10.1007/s11005-016-0842-x. arXiv:1312.2979

[36] Kashani-Poor, A.-K., Troost, J.: Pure super Yang-Mills and exact WKB. JHEP **08**, 160 (2015). 10.1007/JHEP08(2015)160. arXiv:1504.08324

[37] Fioravanti, D., Gregori, D.: Integrability and cycles of deformed gauge theory. Phys. Lett. B **804**, 135376 (2020). 10.1016/j.physletb.2020.135376. arXiv:1908.08030

[38] Hollands, L., Neitzke, A.: Exact WKB and abelianization for the equation. Commun. Math. Phys. **380**, 131–186 (2020). 10.1007/s00220-020-03875-1. arXiv:1906.04271

[39] Ito, K., Kondo, T., Shu, H.: Wall-crossing of TBA equations and WKB periods for the third order ODE. Nucl. Phys. B **979**, 115788 (2022). 10.1016/j.nuclphysb.2022.115788. arXiv:2111.11047

[40] Grassi, A., Mariño, M.: A solvable deformation of quantum mechanics. SIGMA **15**, 025 (2019). 10.3842/SIGMA.2019.025. arXiv:1806.01407

[41] Aganagic, M., Dijkgraaf, R., Klemm, A., Marino, M., Vafa, C.: Topological strings and integrable hierarchies. Commun. Math. Phys. **261**, 451–516 (2006). 10.1007/s00220-005-1448-9. arXiv:hep-th/0312085

[42] Belavin, A.A., Polyakov, A.M., Zamolodchikov, A.B.: Infinite conformal symmetry in two-dimensional quantum field theory. Nucl. Phys. B **241**, 333–380 (1984). 10.1016/0550-3213(84)90052-X

[43] McCoy, B.M., Tracy, C.A., Wu, T.T.: Painleve functions of the third kind. J. Math. Phys. **18**, 1058 (1977). 10.1063/1.523367

[44] Widom, H.: Some classes of solutions to the Toda lattice hierarchy. Commun. Math. Phys. **184**, 653–667 (1997). 10.1007/s002200050078. arXiv:solv-int/9602001

[45] Tracy, C.A., Widom, H.: Asymptotics of a class of solutions to the cylindrical Toda equations. Commun. Math. Phys. **190**, 697–721 (1998). 10.1007/s002200050257. arXiv:solv-int/9701003

[46] Zamolodchikov, A.B.: Painleve III and 2-d polymers. Nucl. Phys. B **432**, 427–456 (1994). 10.1016/0550-3213(94)90029-9. arXiv:hep-th/9409108

[47] Tracy, C.A., Widom, H.: Proofs of two conjectures related to the thermodynamic Bethe ansatz. Commun. Math. Phys. **179**, 667–680 (1996). 10.1007/BF02100102. arXiv:solv-int/9509003

[48] Bonelli, G., Grassi, A., Tanzini, A.: Seiberg-Witten theory as a Fermi gas. Lett. Math. Phys. **107**, 1–30 (2017). 10.1007/s11005-016-0893-z. arXiv:1603.01174

[49] Bonelli, G., Grassi, A., Tanzini, A.: New results in theories from non-perturbative string. Ann. Henri Poincaré **19**, 743–774 (2018). 10.1007/s00023-017-0643-5. arXiv:1704.01517

[50] Bonelli, G., Grassi, A., Tanzini, A.: Quantum curves and -deformed Painlevé equations. Lett. Math. Phys. **109**, 1961–2001 (2019). 10.1007/s11005-019-01174-y. arXiv:1710.11603

[51] Gavrylenko, P., Grassi, A., Hao, Q.: Connecting topological strings and spectral theory via non-autonomous Toda equations, *arXiv e-prints: High Energy Physics - Theory* (4, 2023) , 10.48550/arXiv.2304.11027 [arXiv:2304.11027]

[52] Grassi, A., Hatsuda, Y., Marino, M.: Topological strings from quantum mechanics. Ann. Henri Poincaré **17**, 3177–3235 (2016). 10.1007/s00023-016-0479-4. arXiv:1410.3382

[53] Codesido, S., Grassi, A., Marino, M.: Spectral theory and mirror curves of higher genus. Annales Henri Poincare **18**, 559–622 (2017). 10.1007/s00023-016-0525-2. arXiv:1507.02096

[54] Kashaev, R., Marino, M.: Operators from mirror curves and the quantum dilogarithm. Commun. Math. Phys. **346**, 967–994 (2016). 10.1007/s00220-015-2499-1. arXiv:1501.01014

[55] Kashaev, R., Marino, M., Zakany, S.: Matrix models from operators and topological strings, 2. Annales Henri Poincare **17**, 2741–2781 (2016). 10.1007/s00023-016-0471-z. arXiv:1505.02243

[56] Gamayun, O., Iorgov, N., Lisovyy, O.: Conformal field theory of Painlevé VI. JHEP **10**, 038 (2012) 10.1007/JHEP10(2012)183, 10.1007/JHEP10(2012)038 [arXiv:1207.0787]

[57] Gamayun, O., Iorgov, N., Lisovyy, O.: How instanton combinatorics solves Painlevé VI, V and IIIs. J. Phys. A **46**, 335203 (2013). 10.1088/1751-8113/46/33/335203. arXiv:1302.1832

[58] Bershtein, M.A., Shchechkin, A.I.: q-deformed Painlevé function and q-deformed conformal blocks. J. Phys. A **50**, 085202 (2017). 10.1088/1751-8121/aa5572. arXiv:1608.02566

[59] Iorgov, N., Lisovyy, O., Teschner, J.: Isomonodromic tau-functions from Liouville conformal blocks. Commun. Math. Phys. **336**, 671–694 (2015). 10.1007/s00220-014-2245-0. arXiv:1401.6104

[60] Bershtein, M., Gavrylenko, P., Marshakov, A.: Cluster Toda chains and Nekrasov functions. Theor. Math. Phys. **198**, 157–188 (2019). 10.1134/S0040577919020016. arXiv:1804.10145

[61] Bershtein, M.A., Shchechkin, A.I.: Bilinear equations on Painlevé functions from CFT. Commun. Math. Phys. **339**, 1021–1061 (2015). 10.1007/s00220-015-2427-4. arXiv:1406.3008

[62] Grassi, A., Marino, M.: M-theoretic matrix models. JHEP **1502**, 115 (2015). 10.1007/JHEP02(2015)115. arXiv:1403.4276

[63] Marino, M., Zakany, S.: Exact eigenfunctions and the open topological string. J. Phys. A **50**, 325401 (2017). 10.1088/1751-8121/aa791e. arXiv:1606.05297

[64] Eynard, B., Marino, M.: A Holomorphic and background independent partition function for matrix models and topological strings. J. Geom. Phys. **61**, 1181–1202 (2011). 10.1016/j.geomphys.2010.11.012. arXiv:0810.4273

[65] Gukov, S., Sulkowski, P.: A-polynomial B-model, and quantization. JHEP **02**, 070 (2012). 10.1007/JHEP02(2012)070. arXiv:1108.0002

[66] Maldacena, J.M., Moore, G.W., Seiberg, N., Shih, D.: Exact vs semiclassical target space of the minimal string. JHEP **10**, 020 (2004). 10.1088/1126-6708/2004/10/020. arXiv:hep-th/0408039

[67] Borot, G., Eynard, B.: Geometry of spectral curves and all order dispersive integrable system. SIGMA **8**, 100 (2012). 10.3842/sigma.2012.100. arXiv:1110.4936

[68] Kozcaz, C., Pasquetti, S., Wyllard, N.: A & B model approaches to surface operators and Toda theories. JHEP **08**, 042 (2010). 10.1007/JHEP08(2010)042. arXiv:1004.2025

[69] Gaiotto, D., Gukov, S., Seiberg, N.: Surface defects and resolvents. JHEP **09**, 070 (2013). 10.1007/JHEP09(2013)070. arXiv:1307.2578

[70] Bullimore, M., Kim, H.-C., Koroteev, P.: Defects and quantum Seiberg-Witten geometry. JHEP **05**, 095 (2015). 10.1007/JHEP05(2015)095. arXiv:1412.6081

[71] Pan, Y., Peelaers, W.: Intersecting surface defects and instanton partition functions. JHEP **07**, 073 (2017). 10.1007/JHEP07(2017)073. arXiv:1612.04839

[72] Gorsky, A., Le Floch, B., Milekhin, A., Sopenko, N.: Surface defects and instanton-vortex interaction. Nucl. Phys. B **920**, 122–156 (2017). 10.1016/j.nuclphysb.2017.04.010. arXiv:1702.03330

[73] Aganagic, M., Klemm, A., Vafa, C.: Disk instantons, mirror symmetry and the duality web. Z. Naturforsch. A **57**, 1–28 (2002). 10.1515/zna-2002-1-201. arXiv:hep-th/0105045

[74] Aganagic, M., Vafa, C.: Mirror symmetry, D-branes and counting holomorphic discs, arXiv:hep-th/0012041

[75] Kashani-Poor, A.-K.: The wave function behavior of the open topological string partition function on the conifold. JHEP **04**, 004 (2007). 10.1088/1126-6708/2007/04/004. arXiv:hep-th/0606112

[76] Alday, L.F., Gaiotto, D., Tachikawa, Y.: Liouville correlation functions from four-dimensional gauge theories. Lett. Math. Phys. **91**, 167–197 (2010). 10.1007/s11005-010-0369-5. arXiv:0906.3219

[77] Dimofte, T., Gukov, S., Hollands, L.: Vortex counting and Lagrangian 3-manifolds. Lett. Math. Phys. **98**, 225–287 (2011). 10.1007/s11005-011-0531-8. arXiv:1006.0977

[78] Awata, H., Fuji, H., Kanno, H., Manabe, M., Yamada, Y.: Localization with a surface operator, irregular conformal blocks and open topological string. Adv. Theor. Math. Phys. **16**, 725–804 (2012). 10.4310/ATMP.2012.v16.n3.a1. [arXiv:1008.0574]

[79] Taki, M.: Surface operator Bubbling Calabi-Yau and AGT relation. JHEP **07**, 047 (2011). 10.1007/JHEP07(2011)047. arXiv:1007.2524

[80] Ashok, S.K., Billo, M., Dell’Aquila, E., Frau, M., John, R.R., Lerda, A.: Modular and duality properties of surface operators in N=2* gauge theories. JHEP **07**, 068 (2017). 10.1007/JHEP07(2017)068. arXiv:1702.02833

[81] Ashok, S.K., Billo, M., Dell’Aquila, E., Frau, M., Gupta, V., John, R.R., et al.: Surface operators, chiral rings and localization in =2 gauge theories. JHEP **11**, 137 (2017). 10.1007/JHEP11(2017)137. arXiv:1707.08922

[82] Gukov, S.: *Surface operators*, in *New Dualities of Supersymmetric Gauge Theories* (J. Teschner, ed.), Mathematical Physics Studies, pp. 223–259. Springer, Cham, 2016. 10.1007/978-3-319-18769-3_8. arXiv:1412.7127

[83] Marino, M., Zakany, S.: Wavefunctions, integrability, and open strings. JHEP **05**, 014 (2019). 10.1007/JHEP05(2019)014. arXiv:1706.07402

[84] Eynard, B., Orantin, N.: Invariants of algebraic curves and topological expansion. Commun. Num. Theor. Phys. **1**, 347–452 (2007). 10.4310/CNTP.2007.v1.n2.a4. arXiv:math-ph/0702045

[85] Grassi, A., Kallen, J., Marino, M.: The topological open string wavefunction. Commun. Math. Phys. **338**, 533–561 (2015). 10.1007/s00220-015-2387-8. arXiv:1304.6097

[86] Nekrasov, N.A.: Seiberg-Witten prepotential from instanton counting. Adv. Theor. Math. Phys. **7**, 831–864 (2004). 10.4310/ATMP.2003.v7.n5.a4. arXiv:hep-th/0206161

[87] Bruzzo, U., Fucito, F., Morales, J.F., Tanzini, A.: Multiinstanton calculus and equivariant cohomology. JHEP **0305**, 054 (2003). 10.1088/1126-6708/2003/05/054. arXiv:hep-th/0211108

[88] Flume, R., Poghossian, R.: An Algorithm for the microscopic evaluation of the coefficients of the Seiberg-Witten prepotential. Int. J. Mod. Phys. A **18**, 2541 (2003). 10.1142/S0217751X03013685. arXiv:hep-th/0208176

[89] Tachikawa, Y.: Supersymmetric Dynamics for Pedestrians, vol. 890 of *Lecture Notes in Physics*. Springer, Cham, (2014), 10.1007/978-3-319-08822-8

[90] Its, A., Lisovyy, O., Tykhyy, Y.: Connection problem for the Sine-Gordon/Painlevé III Tau function and irregular conformal blocks. Int. Math. Res. Notices **2014**, 8903–8924 (2015). 10.1093/imrn/rnu209. arXiv:1403.1235

[91] Arnaudo, P., Bonelli, G., Tanzini, A.: On the convergence of Nekrasov functions. Annales Henri Poincaré, 1–37 (2023) 10.1007/s00023-023-01349-3arXiv:2212.06741

[92] Matone, M.: Instantons and recursion relations in SUSY gauge theory. Phys. Lett. B **357**, 342–348 (1995). 10.1016/0370-2693(95)00920-G. arXiv:hep-th/9506102

[93] Flume, R., Fucito, F., Morales, J.F., Poghossian, R.: Matone’s relation in the presence of gravitational couplings. JHEP **04**, 008 (2004). 10.1088/1126-6708/2004/04/008. arXiv:hep-th/0403057

[94] O. Lisovyy and et al., “Unpublished notes.”

[95] Codesido, S., Grassi, A., Marino, M.: Exact results in N=8 Chern-Simons-matter theories and quantum geometry. JHEP **1507**, 011 (2015). 10.1007/JHEP07(2015)011. arXiv:1409.1799

[96] Bonelli, G., Lisovyy, O., Maruyoshi, K., Sciarappa, A., Tanzini, A.: On Painlevé/gauge theory correspondence. Lett. Math. Phys. **107**, 2359–2413 (2017). 10.1007/s11005-017-0983-6. arXiv:1612.06235

[97] Nagoya, H.: Irregular conformal blocks, with an application to the fifth and fourth Painlevé equations. J. Math. Phys. **56**, 123505 (2015). 10.1063/1.4937760. arXiv:1505.02398

[98] Nagoya, H.: *Remarks on irregular conformal blocks and Painlevé III and II tau functions*, *arXiv e-prints: Mathematical Physics* (4, 2018) , 10.48550/arXiv.1804.04782arXiv:1804.04782

[99] Lisovyy, O., Roussillon, J.: On the connection problem for Painlevé I. J. Phys. A **50**, 255202 (2017). 10.1088/1751-8121/aa6e12. arXiv:1612.08382

[100] Gavrylenko, P., Marshakov, A., Stoyan, A.: Irregular conformal blocks. Painlevé III and the blow-up equations, JHEP **12**, 125 (2020). 10.1007/JHEP12(2020)125. arXiv:2006.15652

[101] Bershadsky, fsd M., Cecotti, S., Ooguri, H., Vafa, C,: Holomorphic anomalies in topological field theories. Nucl. Phys. B **405**, 279–304 (1993). 10.1016/0550-3213(93)90548-4. arXiv:hep-th/9302103

[102] Huang, M.-X., Klemm, A.: Holomorphic anomaly in gauge theories and matrix models. JHEP **09**, 054 (2007). 10.1088/1126-6708/2007/09/054. arXiv:hep-th/0605195

[103] Sun, K., Wang, X., Huang, M.-X.: Exact quantization conditions toric Calabi-Yau and nonperturbative topological string. JHEP **01**, 061 (2017). 10.1007/JHEP01(2017)061. arXiv:1606.07330

[104] Grassi, A., Gu, J.: BPS relations from spectral problems and blowup equations. Lett. Math. Phys. **109**, 1271–1302 (2019). 10.1007/s11005-019-01163-1. arXiv:1609.05914

[105] Jeong, S., Nekrasov, N.: Riemann-Hilbert correspondence and blown up surface defects. JHEP **12**, 006 (2020). 10.1007/JHEP12(2020)006. arXiv:2007.03660

[106] Nekrasov, N.: *Blowups in BPS/CFT correspondence, and Painlevé VI*, *Annales Henri Poincaré* (2023) 1–91, 10.1007/s00023-023-01301-5arXiv:2007.03646

[107] Lencsés, M., Novaes, F.: Classical conformal blocks and accessory parameters from isomonodromic deformations. JHEP **04**, 096 (2018). 10.1007/JHEP04(2018)096. arXiv:1709.03476

[108] Bershtein, M., Gavrylenko, P., Grassi, A.: Quantum spectral problems and isomonodromic deformations. Commun. Math. Phys. **393**, 347–418 (2022). 10.1007/s00220-022-04369-y. arXiv:2105.00985

[109] da Cunha, B. C., Cavalcante, J. a. P.: *Expansions for semiclassical conformal blocks*, *arXiv e-prints: High Energy Physics - Theory* (11, 2022) , 10.48550/arXiv.2211.03551arXiv:2211.03551

[110] Gu, J.: *Relations between Stokes constants of unrefined and Nekrasov-Shatashvili topological strings*, *arXiv e-prints: High Energy Physics - Theory* (7, 2023) , 10.48550/arXiv.2307.02079arXiv:2307.02079

[111] Katz, S.H., Klemm, A., Vafa, C.: Geometric engineering of quantum field theories. Nucl. Phys. B **497**, 173–195 (1997). 10.1016/S0550-3213(97)00282-4. arXiv:hep-th/9609239

[112] Iqbal, A., Kashani-Poor, A.-K.: SU(N) geometries and topological string amplitudes. Adv. Theor. Math. Phys. **10**, 1–32 (2006). 10.4310/ATMP.2006.v10.n1.a1. arXiv:hep-th/0306032

[113] Klemm, A., Lerche, W., Mayr, P., Vafa, C., Warner, N.P.: Selfdual strings and N=2 supersymmetric field theory. Nucl. Phys. B **477**, 746–766 (1996). 10.1016/0550-3213(96)00353-7. arXiv:hep-th/9604034

[114] Iqbal, A., Kozcaz, C., Vafa, C.: The Refined topological vertex. JHEP **0910**, 069 (2009). 10.1088/1126-6708/2009/10/069. arXiv:hep-th/0701156

[115] Taki, M.: Refined topological vertex and instanton counting. JHEP **03**, 048 (2008). 10.1088/1126-6708/2008/03/048. arXiv:0710.1776

[116] Aganagic, M., Cheng, M.C., Dijkgraaf, R., Krefl, D., Vafa, C.: Quantum geometry of refined topological strings. JHEP **1211**, 019 (2012). 10.1007/JHEP11(2012)019. arXiv:1105.0630

[117] Codesido, S., Gu, J., Marino, M.: Operators and higher genus mirror curves. JHEP **02**, 092 (2017). 10.1007/JHEP02(2017)092. arXiv:1609.00708

[118] Grassi, A., Marino, M.: The complex side of the TS/ST correspondence. J. Phys. A **52**, 055402 (2019). 10.1088/1751-8121/aaec4b. arXiv:1708.08642

[119] Laptev, A., Schimmer, L., Takhtajan, L.A.: Weyl type asymptotics and bounds for the eigenvalues of functional-difference operators for mirror curves. Geom. Funct. Anal. **26**, 288–305 (2016). 10.1007/s00039-016-0357-8. arXiv:1510.00045

[120] Iwaki, K., Saenz, A.: Quantum curve and the first Painleve equation. SIGMA **12**, 011 (2016). 10.3842/SIGMA.2016.011. arXiv:1507.06557

[121] Iwaki, K.: 2-Parameter -Function for the First Painlevé equation: topological recursion and direct monodromy problem via exact WKB analysis. Commun. Math. Phys. **377**, 1047–1098 (2020). 10.1007/s00220-020-03769-2. arXiv:1902.06439

[122] Marchal, O., Orantin, N.: Isomonodromic deformations of a rational differential system and reconstruction with the topological recursion: the case. J. Math. Phys. **61**, 061506 (2020). 10.1063/5.0002260. arXiv:1901.04344

[123] Marchal, O., Orantin, N.: Quantization of hyper-elliptic curves from isomonodromic systems and topological recursion. J. Geom. Phys. **171**, 104407 (2022). 10.1016/j.geomphys.2021.104407. arXiv:1911.07739

[124] Kostov, I.K.: Solvable statistical models on a random lattice. Nucl. Phys. Proc. Suppl. **45A**, 13–28 (1996). 10.1016/0920-5632(95)00611-7. arXiv:hep-th/9509124

[125] Kostov, I.K.: O() vector model on a planar random lattice: spectrum of anomalous dimensions. Mod. Phys. Lett. A **4**, 217 (1989). 10.1142/S0217732389000289

[126] Kostov, I.K., Staudacher, M.: Multicritical phases of the O(n) model on a random lattice. Nucl. Phys. B **384**, 459–483 (1992). 10.1016/0550-3213(92)90576-W. arXiv:hep-th/9203030

[127] Kostov, I.K.: Exact solution of the six vertex model on a random lattice. Nucl. Phys. B **575**, 513–534 (2000). 10.1016/S0550-3213(00)00060-2. arXiv:hep-th/9911023

[128] Eynard, B., Kristjansen, C.: More on the exact solution of the O(n) model on a random lattice and an investigation of the case . Nucl. Phys. B **466**, 463–487 (1996). 10.1016/0550-3213(96)00104-6. arXiv:hep-th/9512052

[129] Eynard, B., Kristjansen, C.: Exact solution of the O(n) model on a random lattice. Nucl. Phys. B **455**, 577–618 (1995). 10.1016/0550-3213(95)00469-9. arXiv:hep-th/9506193

[130] Suyama, T.: On large N solution of N=3 Chern-Simons-adjoint theories. Nucl. Phys. B **867**, 887–912 (2013). 10.1016/j.nuclphysb.2012.10.017. arXiv:1208.2096

[131] Byrd, P. F., Friedman, M. D.: *Handbook of Elliptic Integrals for Engineers and Scientists*. Die Grundlehren der mathematischen Wissenschaften. Springer, Berlin, Heidelberg, 2 ed., 1971, 10.1007/978-3-642-65138-0

[132] Witten, E.: *Quantum background independence in string theory*, in *Conference on Highlights of Particle and Condensed Matter Physics (SALAMFEST)*, 6, 1993. arXiv:hep-th/9306122

[133] Aganagic, M., Bouchard, V., Klemm, A.: Topological strings and (almost) modular forms. Commun. Math. Phys. **277**, 771–819 (2008). 10.1007/s00220-007-0383-3. arXiv:hep-th/0607100

[134] Bouchard, V., Klemm, A., Marino, M., Pasquetti, S.: Remodeling the B-model. Commun. Math. Phys. **287**, 117–178 (2009). 10.1007/s00220-008-0620-4. arXiv:0709.1453

[135] Marino, M.: Open string amplitudes and large order behavior in topological string theory. JHEP **03**, 060 (2008). 10.1088/1126-6708/2008/03/060. arXiv:hep-th/0612127

[136] Eynard, B., Orantin, N.: Computation of Open Gromov-Witten Invariants for Toric Calabi-Yau 3-folds by topological recursion, a proof of the BKMP conjecture. Commun. Math. Phys. **337**, 483–567 (2015). 10.1007/s00220-015-2361-5. arXiv:1205.1103

[137] Borot, G., Eynard, B.: Enumeration of maps with self avoiding loops and the O(n) model on random lattices of all topologies. J. Stat. Mech. **1101**, P01010 (2011). 10.1088/1742-5468/2011/01/P01010. [arXiv:0910.5896]

[138] Borot, G., Eynard, B., Orantin, N.: Abstract loop equations, topological recursion and new applications. Commun. Num. Theor. Phys. **09**, 51–187 (2015). 10.4310/CNTP.2015.v9.n1.a2. [arXiv:1303.5808]

[139] Hatsuda, Y., Moriyama, S., Okuyama, K.: Exact results on the ABJM fermi gas. JHEP **10**, 020 (2012). 10.1007/JHEP10(2012)020. [arXiv:1207.4283]

[140] François, M., Grassi, A.: “Work in progress.”

[141] Bonelli, G., Globlek, F., Tanzini, A.: Toda equations for surface defects in SYM and instanton counting for classical Lie groups. J. Phys. A **55**, 454004 (2022). 10.1088/1751-8121/ac9e2a. arXiv:2206.13212

[142] Cecotti, S., Fendley, P., Intriligator, K.A., Vafa, C.: A New supersymmetric index. Nucl. Phys. B **386**, 405–452 (1992). 10.1016/0550-3213(92)90572-S. arXiv:hep-th/9204102

[143] Gavrylenko, P., Lisovyy, O.: Fredholm determinant and Nekrasov sum representations of isomonodromic tau functions. Commun. Math. Phys. **363**, 1–58 (2018). 10.1007/s00220-018-3224-7. arXiv:1608.00958

[144] Gavrylenko, P., Lisovyy, O.: Pure gauge theory partition function and generalized Bessel kernel, in *Proc. Symp. Pure Math.* (A.-K. Kashani-Poor, R. Minasian, N. Nekrasov and B. Pioline, eds.), vol. 18, pp. 181–208, (2018). arXiv:1705.01869

[145] Desiraju, H.: Fredholm determinant representation of the homogeneous painlevé ii -function. Nonlinearity **34**, 6507–6538 (2020)

[146] Del Monte, F., Desiraju, H., Gavrylenko, P.: Isomonodromic tau functions on a torus as Fredholm determinants, and charged partitions. Commun. Math. Phys. **398**, 1029–1084 (2023). 10.1007/s00220-022-04458-y. arXiv:2011.06292

[147] Iqbal, A., Kozcaz, C., Vafa, C.: The refined topological vertex. JHEP **10**, 069 (2009). 10.1088/1126-6708/2009/10/069. arXiv:hep-th/0701156

[148] Cheng, S., Sułkowski, P.: Refined open topological strings revisited. Phys. Rev. D **104**, 106012 (2021). 10.1103/PhysRevD.104.106012. arXiv:2104.00713

[149] Hatsuda, Y., Marino, M., Moriyama, S., Okuyama, K.: Non-perturbative effects and the refined topological string. JHEP **1409**, 168 (2014). 10.1007/JHEP09(2014)168. arXiv:1306.1734

[150] Kashani-Poor, A.-K.: Quantization condition from exact WKB for difference equations. JHEP **06**, 180 (2016). 10.1007/JHEP06(2016)180. arXiv:1604.01690

[151] Faddeev, L.: Discrete Heisenberg-Weyl group and modular group. Lett. Math. Phys. **34**, 249–254 (1995). 10.1007/BF01872779. arXiv:hep-th/9504111

[152] Kashaev, R.: *On the spectrum of Dehn twists in quantum Teichmüller theory*, in *Physics and combinatorics*, pp. 63–81, World Scientific, 2001. 10.1142/9789812810007_0004arXiv:math/0008148

[153] Takhtajan, L. A., Faddeev, L. D.: On the spectral theory of a functional-difference operator in conformal field theory, *Izvestiya: Mathematics* **79** (4, 2015) 388, 10.1070/IM2015v079n02ABEH002747arXiv:1408.0307

[154] Wang, X., Zhang, G., Huang, M.-X.: New exact quantization condition for Toric Calabi-Yau geometries. Phys. Rev. Lett. **115**, 121601 (2015). 10.1103/PhysRevLett.115.121601. arXiv:1505.05360 26430981 10.1103/PhysRevLett.115.121601

[155] Hatsuda, Y., Marino, M.: Exact quantization conditions for the relativistic Toda lattice. JHEP **05**, 133 (2016). 10.1007/JHEP05(2016)133. arXiv:1511.02860

[156] Hatsuda, Y.: ABJM on ellipsoid and topological strings. JHEP **07**, 026 (2016). 10.1007/JHEP07(2016)026. arXiv:1601.02728

[157] Adamchik, V. S.: *Contributions to the Theory of the Barnes Function*, *arXiv e-prints: Classical Analysis and ODEs* (3, 2003) , [arXiv:math/0308086]

[158] Hatsuda, Y., Moriyama, S., Okuyama, K.: Instanton effects in ABJM theory from fermi gas approach. JHEP **01**, 158 (2013). 10.1007/JHEP01(2013)158. arXiv:1211.1251

[159] Wolfram Research, “EllipticK.” https://reference.wolfram.com/language/ref/EllipticK.html (2022)

[160] Wolfram Research, “EllipticF.” https://reference.wolfram.com/language/ref/EllipticF.html (2022)

[161] Wolfram Research, “EllipticE.” https://reference.wolfram.com/language/ref/EllipticE.html (2022)

[162] Wolfram Research, “EllipticPi.” https://reference.wolfram.com/language/ref/EllipticPi.html (2022)

